# Identification of drought-tolerant ideotype in wheat using multi-trait genotype-ideotype distance index (MGIDI) and comprehensive evaluation value

**DOI:** 10.1038/s41598-026-46140-6

**Published:** 2026-04-17

**Authors:** Mohammad-bagher Zahedi, Sirus Tahmasebi, Maryam Salami, Sivakumar Sukumaran, Bahram Heidari

**Affiliations:** 1 Department of Plant Production and Genetics, School of Agriculture, Shiraz University, Shiraz, 7144165186 Iran; 2 Department of Seed and Plant Improvement Research, Fars Agriculture and Natural Resources Research and Education Center, Agricultural Research, Education and Extension Organization (AREEO), Shiraz, Iran; 3 Department of Plant & Environmental Sciences & Advanced Plant Technology Program, Clemson University, Clemson, SC 29634 USA

**Keywords:** Drought tolerance, Ideotype, Membership function, Multi-environment trials (METs), Multivariate analysis, Selection gains (SG), Genetics, Plant sciences

## Abstract

Due to the growing impact of climate change, particularly in arid regions, there is a pressing need for high-yield and drought-tolerant wheat (*Triticum aestivum* L.). Recombinant inbred lines (RILs) from a cross between high-yielding ‘SeriM82’ and drought-tolerant 'Babax' offer a valuable genetic resource. This study evaluated 167 RILs along with their two parents under two irrigation regimes over 2 years to identify optimal genotypes using morpho-physiological traits, principal component analysis (PCA), drought indices, comprehensive evaluation value (*D*), drought resistance index (DRI), and multi-trait genotype-ideotype distance index (MGIDI). Drought stress significantly reduced all studied traits, with chlorophyll concentration (SPAD), relative water content (RWC), and grain yield (GY) most affected. The population showed high genetic diversity. RWC was strongly positively correlated with biological yield (BY) and GY. GY under drought exhibited a strong positive relationship with the stress tolerance index (STI;* r* = 0.96). Based on PCA and *D* values, RILs 76, 104, and 144 demonstrated superior performance under drought. Cluster analysis categorized RILs into five groups; one cluster showed high relative stress index and STI, indicating high drought tolerance. In MGIDI analysis, STI had a higher selection differentials percentage. Based on combined MGIDI rankings and DRI, RILs 21, 23, 25, 48, 54, 64, 70, 77, 90, 139, 149, and 162 were identified as the most drought-tolerant. The results demonstrate that integrating DRI and MGIDI provides a rapid, cost-effective method for selecting high-yielding wheat genotypes with resilience across varying water conditions, facilitating accelerated breeding for climate resilience.

**Supplementary Information:**

The online version contains supplementary material available at 10.1038/s41598-026-46140-6.

## Introduction

Wheat, scientifically known as *Triticum aestivum* L., stands as the second most crucial cereal crop globally, serving as a fundamental food source for a significant portion of the world's population. According to the Food and Agriculture Organization (FAO)^1^, global wheat production for the 2024/25 period is estimated to reach 801 million metric tons (MMT), showing an increase from the previous year's 793 MMT. It is worth noting that between 24 and 50% of the world's population relies on wheat for their daily intake of essential calories and proteins^2^. However, the challenge lies in the steady increase of the world's population, with projections indicating a rise from 8.2 billion people in 2024 to nearly 10.3 billion individuals within the next 50–60 years^3^. This data underscores the critical role that wheat plays in meeting the dietary needs of millions worldwide. The ongoing climate changes, characterized by an increase in the frequency and severity of climatic extreme events and adverse weather conditions, pose a threat to global wheat production. This threat extends to countries like Iran, where rainfall is erratic, and access to irrigation water is not always guaranteed^4–7^. Drought is considered as one of the most severe abiotic stresses that can impact crop growth, productivity, and survival, especially when compared to other adverse weather conditions and climatic extreme events^8–10^. In Iran, the frequency and severity of drought stresses are expected to rise due to future climate change^11,12^. Consequently, the risk of yield losses and potential crop failure is likely to increase under these future climatic conditions^13^.

Drought exerts a significant influence on a variety of physiological processes in wheat, including photosynthesis, transpiration, and stomatal conductance^14–16^. This disruption ultimately impedes the movement of photosynthates from the source to the sink, leading to an imbalance between assimilation sources and sinks that can result in a reduction in grain yield, sometimes as high as 90%^17,18^. The impact of drought on wheat organs, such as leaves, tillers, and spikes, as well as growth stages like tillering, jointing, booting, heading, anthesis, and grain filling, can vary depending on factors such as the severity and duration of drought stress, crop development stage, and genetic variability^19,20^. Particularly, vulnerable to drought are the booting and grain filling stages in wheat, as these are crucial periods when nutrients are transferred from the stem and leaves to the seeds^21,22^. Drought stress during the booting stage of wheat production can significantly reduce photosynthetic capacity and impede the accumulation of dry matter in the shoots, ultimately leading to decreased grain weight, fewer grains, and lower crop yields. This highlights the critical importance of managing water resources effectively in wheat production. Furthermore, the impacts of climate change are increasingly evident in wheat-growing regions, underscoring the urgent need to optimize yield through efficient water utilization. To tackle these challenges, it is essential to identify wheat genotypes that demonstrate enhanced drought tolerance. Research has shown the detrimental effects of drought stress on wheat production and grain yield^23–25^. In light of these findings, it is crucial to prioritize the development of drought-resistant wheat varieties. Identifying wheat genotypes with improved drought tolerance is essential for enhancing agricultural sustainability and ensuring food security in the face of changing environmental conditions^26–28^.

One of the main challenges in developing new drought-resistant wheat genotypes is the need to screen a wide range of germplasms to identify those with the desired traits^29,30^. Wheat displays varying degrees of sensitivity to drought, with certain varieties showing greater tolerance than others^31,32^. In breeding programs, researchers have employed various eco-physiological parameters to assess wheat performance under water stress conditions^33–39^. While grain yield is typically the primary selection criterion for improving grain production in different environments, the significant interactions between genotype and environment require repeated evaluations of genotypes in various locations and over multiple years to accurately identify superior genotypes with high grain yield^40^. Several statistical indices have been developed to evaluate the performance of various genotypes under different water conditions. The stress susceptibility index (SSI), drought resistance index (DRI), stress tolerance index (STI), yield stability index (YSI), relative stress index (RSI), and tolerance index (TOL) are established based on mathematical relationships between the response of plants under well-watered and stress conditions^34,41,42^. When assessing genotypes under well-watered and limited water irrigation conditions, the RSI, YSI, and STI have been identified as effective indices^42–45^. A strong methodological foundation for selecting superior genotypes requires a clear distinction between two key breeding objectives: stress tolerance and stability. Stress tolerance is a genotype's capacity to maintain productivity under adverse versus optimal conditions, measured by indices like STI or YSI^42^. Stability, in contrast, reflects consistent performance across variable environments (e.g., different years or locations), indicating minimal genotype-by-environment (G × E) interaction. For arid and semi-arid zones, the ideal cultivar integrates both high stress tolerance and high stability to ensure resilient and reliable yields under unpredictable stress. While univariate indices effectively assess tolerance, evaluating stability demands multivariate analyses and multi-environment trials, both essential for a comprehensive selection strategy^45^. While most drought indices focus on analyzing individual components like grain yield in different environments, it is essential to choose a method that considers multiple traits to provide a comprehensive evaluation of drought tolerance in genotypes. Multivariate techniques, such as hierarchical cluster analysis (HCA), principal component analysis (PCA), and linear discriminant analysis play a crucial role in examining and quantifying the relationship between dependent and response variables^26,46,47^. Rather than focusing on a single variable, these methods allow for a more comprehensive and specific understanding of the connections between different variables^48,49^. While these techniques have proven effective in assessing genetic variation in drought resistance^50,51^and revealing correlations between drought-tolerant indices and grain yield^41,52^, determining the ranking of genotypes based on various traits remains a challenge. The innovative Multi-Trait Genotype-Ideotype Distance Index (MGIDI) approach offers a powerful solution to this issue, enabling the ranking of genotypes based on multiple traits with significant genetic differentials^53,54^. The MGIDI method is both robust and adaptable, making it well-suited for selecting superior genotypes across a range of crops, considering factors such as productivity^55^, quality^56^, physiology^57^, and phenology^58^. By addressing multicollinearity and identifying key variables for genetic gains, the MGIDI index simplifies the selection process by consolidating various characteristics into a single value^59^. This feature enables breeders to utilize flexible selection criteria by assigning weights to various traits and assessing the strengths and weaknesses of each genotype^60^.

Previous studies have utilized various stress indices to identify wheat genotypes that are resilient to drought^34,41–45^. Additionally, a team of researchers utilized the MGIDI has been used to select high-performing wheat genotypes for further evaluation of plants to stresses based on different traits^56,61–63^. Despite these efforts, there has not been a comprehensive study that integrates multiple stress indicators and multivariate analyses to define a wheat ideotype, which is a hypothetical genotype with the best combination of traits across various environmental conditions. In this study, our objectives were to: (1) assess the agronomic potential and genetic diversity of 167 wheat recombinant inbred lines (RILs) along with their two parents under different irrigation conditions over two years in Fars, an arid region of Iran, (2) identify drought-tolerant lines for future research using multivariate analysis and drought susceptibility indices, and (3) develop MGIDI for screening wheat genotypes grown under water stress conditions and compare the selection outcomes with those obtained using traditional drought indices. By employing a combination of multivariate techniques, we aim to enhance the screening, clustering, and characterization of wheat genotypes. This advancement will significantly contribute to the refinement of crop improvement strategies and drought tolerance studies in field crops.

## Results

### Evaluation of wheat RILs under drought stress condition

**Fig. 1:**
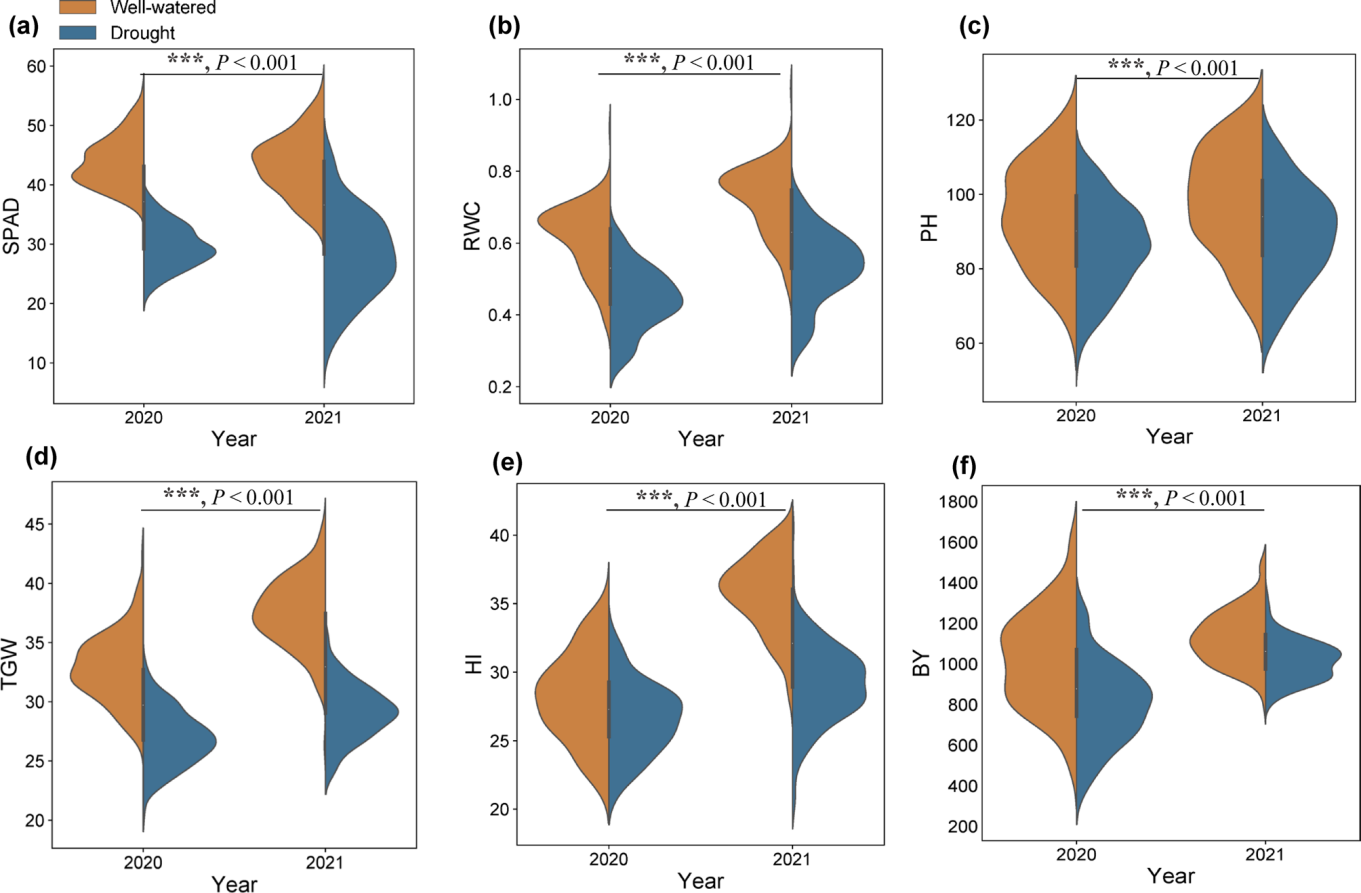

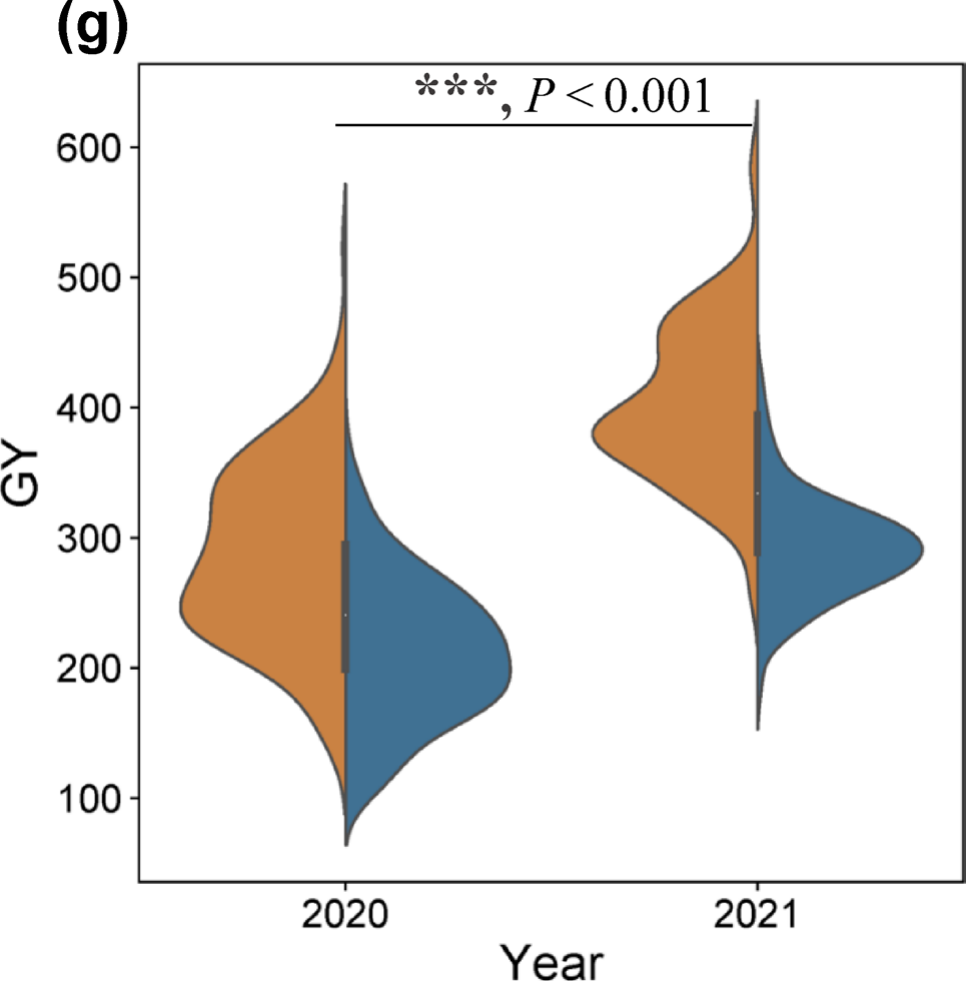
The violin plot shows the phenotypic distribution of (**a**) SPAD, (**b**) RWC, (**c**) PH, (**d**) TGW, (**e**) HI, (**f**) BY, and (**g**) GY in the 167 wheat recombinant inbred lines (RILs) along with their two parents under two irrigation regimes, well-watered and drought stress conditions. SPAD, RWC, PH, TGW, HI, BY, and GY were the abbreviations estimation of chlorophyll using SPAD, relative water content, plant height, thousand grain weight, harvest index, biological yield, and grain yield. *** Indicate significant difference between years (t-test: ***P < 0.001).

The results of the c-ANOVA indicated that both irrigation and RIL had a significant impact on relative water content (RWC), plant height (PH), biomass yield (BY), and grain yield (GY) (Table 1). The interaction between irrigation regime and RIL was significant for most physiological and morphological traits, though not for final yield components (GY, HI, BY) and SPAD. Interestingly, the effect of year was not significance for the traits (Table 2). The results of the skewness and kurtosis tests indicated that no significant deviation from normal distribution (Table 2). Violin plots clearly illustrate the differentiation between drought stress and well-watered conditions (Fig. 1). The distribution shape of all traits, characterized by narrow ends and a wider middle, suggests that the trait distribution is centered around the median. There was a wide variation in all measured traits among wheat RILs under drought stress, with the largest and smallest variations observed in BY and RWC, respectively (Table 2).

**Table 1:** Analysis of variance (ANOVA) for different traits in RILs of wheat using alpha-lattice design under two irrigation regimes, well-watered (WW) and drought stress (DS) conditions across two years (2020–2021 and 2021–2022).

Source of variation	d. f	Mean sum of squares													
		RWC		SPAD		PH		TGW		HI		BY		GY	
		WW	DS	WW	DS	WW	DS	WW	DS	WW	DS	WW	DS	WW	DS
*Year 2020–2021*															
Replications	2	0.04**	0.04**	416.7**	416.6**	1367.3^ns^	1267.4^ns^	39.4**	1.5	59.1**	8.6*	77,206*	76,843**	1372^ns^	7318**
Treatment (unadjusted)	168	1.14**	0.6**	5794.1**	2674.2**	30,732.5**	24,132.5**	3256.6**	2190.3**	2412.2**	2135.7**	3,295,720**	2,046,508**	258,708**	143,744**
Block in rep (adjusted)	36	0.004	0.009	38.3	21.12	2012.3	1212.35	1.9	1.3	3.3	2.04	24,768	9298	2117	788
Intrablock error	300	0.006	0.006	38.3	15.71	1969.9	1403.2	2.3	1.3	4.1	2.7	17,889	9485	1581	696
*Year 2021–2022*															
Replications	2	0.008	0.04*	22.2	263.45**	18.5	221.7**	132.3**	9.06	3850**	197.10**	113,420**	468,454**	3440	18,304**
Treatment (unadjusted)	168	1.58**	0.87**	5724.6**	2550.2**	29,535.4**	24,299.8**	4233.5**	2555.7**	184.1**	2575.8**	3,873,509**	3,123,176**	494,254**	260,843**
Block in rep (adjusted)	36	0.008	0.01	37.6	39.43	36.9	11.6	9	4.99	20.1	41.67	46,750	54,504	2836	1710
Intrablock error	300	0.007	0.009	34.3	48.29	24.3	16.3	6	3.45	11.7	23.86	19,482	14,965	1993	1109
*Pooled analysis*															
Year	1	4.94**		378.33**		2912.60 **		5866.36**		13,089.33**		13,042,476.94**		4,864,818.99**	
Block (year)	4	0.05**		163.70**		309.56^ns^		54.37**		215.98**		262,466.65**		10,008.08**	
Irrigation	1	16.23**		108,356.56**		48,947.24**		25,767.46**		8473.70**		13,989,324.08**		4,242,900.94**	
Year × Irrigation	1	0.04**		130.10*		239.43^ns^		760.75**		3092.21**		1,412,685.22**		161,918.46**	
Irrigation* Block (year)	4	0.01^ns^		395.79**		510.12^ns^		36.74**		8.45^ns^		105,494.86**		5209.25**	
RIL	168	0.08**		78.45**		2639.66**		43.96**		35.95**		177,977.73**		19,314.76**	
Year × RIL	168	0.0008^ns^		66.80 **		552.18^ns^		14.93**		32.97**		133,293.35 **		12,063.07**	
RIL × Irrigation	168	0.01**		78.84**		573.09^ns^		16.89**		13.94*		24,579.26**		2524.64**	
Year × RIL × Irrigation	168	0.0007^ns^		91.29 **		490.31^ns^		4.41**		12.72^ns^		25,268.81 **		3117.10**	
Error	1344	0.007		34.13		506.93		3.38		11.26		17,423.9		1400.25	
CV		15.24		16.10		24.34		5.82		11.22		13.30		12.55	

*Significant at the 0.05 probability level, **significant at the 0.01 probability level. ns: not significant, S.O.V: source of variation, RIL: recombinant inbred line, d. f.: degree of freedom, CV: coefficient of variation. SPAD, RWC, PH, TGW, HI, BY, and GY were the abbreviations estimation of chlorophyll using SPAD, relative water content, plant height, thousand grain weight, harvest index, biological yield, and grain yield.

**Table 2:** Descriptive statistics and phenotypic variation of agronomic and yield related traits in 167 wheat recombinant inbred lines (RILs) along with their two parents grown at two irrigation regimes, well-watered (WW) and drought stress (DS) in two field trials (2020–2021 and 2021–2022).

Trait	Environment	Range	Mean ± SD	Skew	Kurt	ECV (%)	PCV (%)	GCV (%)	H^2^ (%)	GA	GAM (%)
SPAD	WW20	28.10–73	43.75 ± 6.61	0.88	1.18	0.10	9.35	9.35	23.78	842.93	1926.25
	DS20	16.70–48.30	29.64 ± 5	0.29	0.12	7.86	12	9.07	57.13	418.87	1413.18
	WW21	19.70–62.80	43.40 ± 6.91	− 0.20	0.17	7.82	11.46	8.38	53.40	547.62	1261.81
	DS21	9.40–60.10	28.27 ± 9.12	0.45	0.12	14.05	25.24	20.97	69.02	1014.93	3590.15
									18.79		
RWC (%)	WW20	0.29–1.07	0.61 ± 0.11	− 0.51	0.32	7.71	15	12.86	73.58	13.87	2274.02
	DS20	0.23–0.70	0.44 ± 0.11	0.03	− 0.99	10.99	19.66	16.29	68.71	12.25	2783.37
	WW21	0.34–1.16	0.72 ± 0.11	− 0.67	0.49	6.92	12.78	10.74	70.69	13.40	1861.55
	DS21	0.26–0.77	0.53 ± 0.12	− 0.14	− 1.10	10.80	18.49	15	65.83	13.29	2507.65
									91.19		
PH (cm)	WW20	59.80–123.40	94.56 ± 13.90	− 0.09	− 0.77	26.57	31.59	17.09	29.26	1838.92	1904.83
	DS20	53.40–113.20	86.00 ± 11.81	− 0.11	− 0.52	2.16	13.40	13.22	97.38	2312.51	2688.66
	WW21	60.20–126.80	98.24 ± 14.49	− 0.20	− 0.78	2.97	14.17	13.85	95.59	2742.22	2791.07
	DS21	58.80–119.80	89.10 ± 13.11	− 0.04	− 0.52	2.57	14.25	14.01	96.73	2530.35	2839.90
									79.59		
TGW (g)	WW20	25–44.51	32.82 ± 3.09	0.17	0.14	2.64	8.57	8.15	90.49	524.79	1598.50
	DS20	19.63–33.64	26.92 ± 2.48	0.12	− 0.25	2.48	8.53	8.16	91.51	433.07	1608.74
	WW21	3.46–48.85	37.45 ± 3.61	− 1.60	− 1.90	3.87	7.73	6.69	74.92	447.26	1194.28
	DS21	20.71–37.63	29.10 ± 2.74	− 0.14	− 0.11	3.77	7.79	6.81	76.53	357.41	1228.20
									72.52		
HI (%)	WW20	19.14–38.96	28.17 ± 3.62	− 0.03	− 0.37	4.13	11.37	10.60	86.82	573.55	2035.32
	DS20	17.12–34.88	26.55 ± 2.90	0.04	− 0.14	3.52	9.73	9.07	86.88	462.64	1741.87
	WW21	21.31–44.89	35.72 ± 4	− 0.21	− 0.05	5.73	7.41	4.70	40.28	219.89	615.42
	DS21	13.37–44.35	29.16 ± 5.07	0.12	0.10	9.52	10.35	4.07	15.47	96.29	330.11
									49.76		
BY (g/m^2^)	WW20	170–1810	1021.37 ± 261.06	0.07	− 0.17	7.71	23.10	21.78	88.85	43,198.92	4229.51
	DS20	289–1409	802.47 ± 211.81	0.19	− 0.20	6.99	24.42	23.4	91.78	37,066.37	4618.98
	WW21	626–1658	1128.98 ± 178.71	0.36	− 0.07	7.65	11.43	8.50	55.23	14,694.51	1301.57
	DS21	612–1500	1015.65 ± 155.33	0.25	− 0.09	7.87	9.62	5.53	33.08	6664.79	656.21
									65.69		
GY (g/m^2^)	WW20	50–590	285.86 ± 74.97	0.88	1.18	8.17	23.58	22.12	87.98	12,220.51	4275
	DS20	90–388	212.25 ± 58.02	0.28	− 0.23	7.22	25.28	24.23	91.83	10,153.14	4783.58
	WW21	220–668	401.68 ± 69.29	0.36	0.17	6.56	14.55	12.99	79.68	9596.62	2389.06
	DS21	144–446	292.33 ± 48.38	0.12	0.20	6.76	13.22	11.36	73.84	5882.63	2012.26
									69.44		

SPAD, RWC, PH, TGW, HI, BY, and GY were the abbreviations estimation of chlorophyll using SPAD, relative water content, plant height, thousand grain weight, harvest index, biological yield, and grain yield. WW20, DS20, WW21, and DS21 were the codes of the two irrigation regimes during two years: well-watered in 2020–2021, drought stress in 2020–2021, well-watered in 2021–2022, and drought stress in 2021–2022. SD, Skew, Kurt, ECV, PCV, GCV, *H*^2^, GA, and GAM were the abbreviations of standard deviation, Skewness, which reflects the asymmetry of the probability distribution of a real-valued random variable about its mean, Kurtosis, which reflects the ‘tailedness’ of the probability distribution of a real-valued random variable environmental coefficient of variation, phenotypic coefficient of variation, genotypic coefficient of variation, heritability in broad sense, genetic advance, and genetic advance as the percentage of the mean of the studied traits at two irrigation regimes under two years. The estimated broad-sense heritability for the combined environments was displayed in italic bold font at the end of the row of traits.

**Fig. 2:**
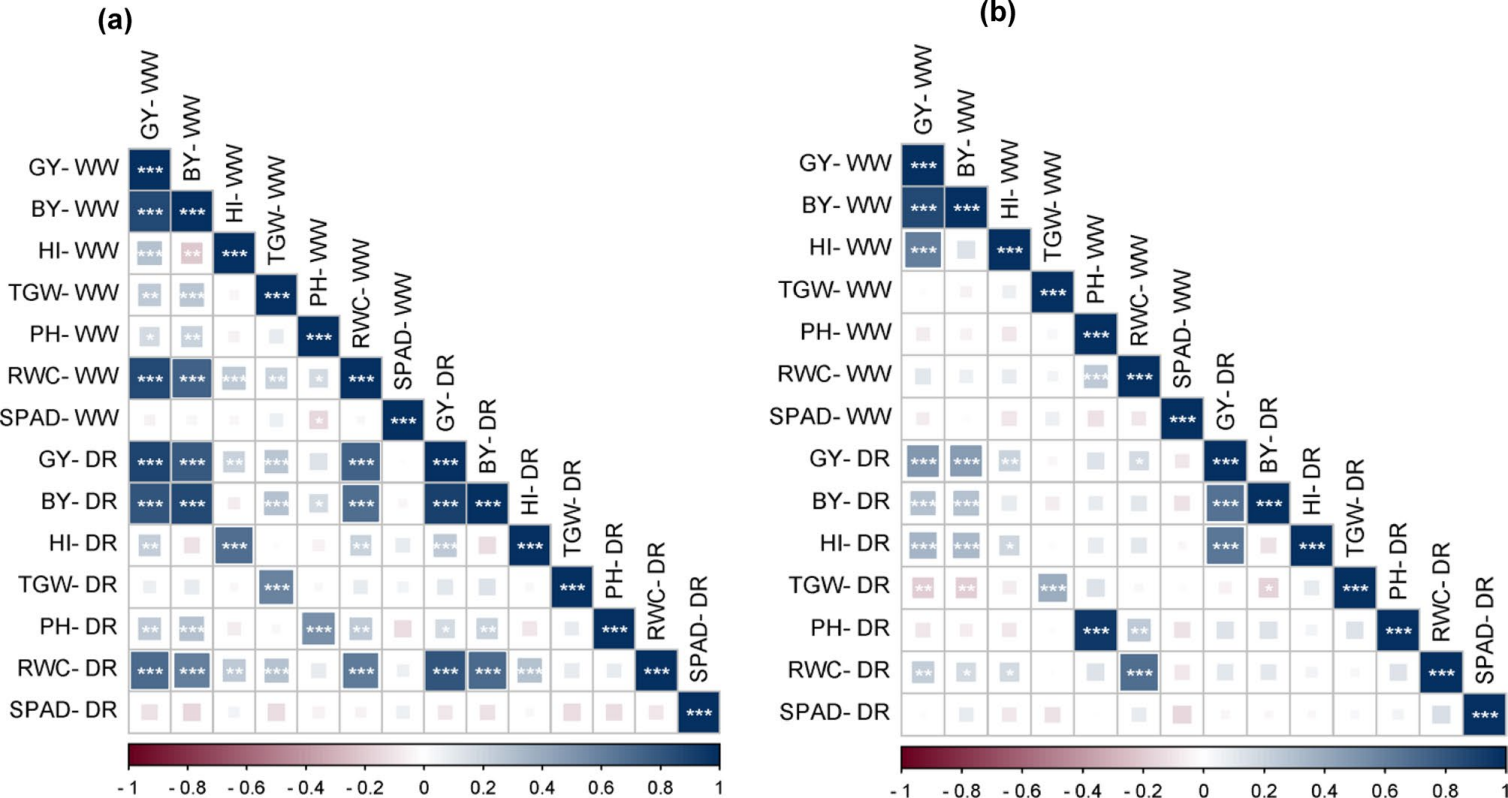
Heat map showing the correlation between the morpho-physiological and yield related traits under two irrigation regimes, well-watered (WW) and drought stress (DR) conditions in two growing seasons. (**a**) Correlation between the traits under WW and DR conditions in 2020–2021. (**b**) Correlation between the traits under WW and DS conditions in 2021–2022. Traits represent as grain yield (GY), biological yield (BY), harvest index (HI), thousand grain weight (TGW), plant height (PH), relative water content (RWC), estimation of chlorophyll using SPAD (SPAD). A color scale showing the correlation values ranging from dark red, − 1, to white, 0, to 1, dark blue is shown below the heat map.

Drought stress induced a consistent and significant reduction in the mean performance of all traits (Fig. 1). The severity of the reduction, however, varied clearly among traits, defining a ranking of drought susceptibility. Physiological traits related to photosynthetic capacity were most sensitive: SPAD and RWC exhibited the strongest reduces under stress, averaging reductions of approximately 34% and 28%, respectively, across both years (*P* < 0.001; Fig. 2a, g). These were followed by reproductive and yield-component traits, with GY itself declining by an average of 27%. In contrast, traits such as PH, TGW, and HI showed more moderate but significant reductions, with HI being the least affected (Fig. 2).

In the 2020–2021 season, average yield reduction from well-watered to drought conditions was 41.7%, ranging from − 8.8 to 82.7%, with superior lines showing lower yield losses (38.2%). In contrast, the 2021–2022 season showed a significantly lower average reduction (28.8%) with a range of − 8.7 to 77.7%, where superior lines again demonstrated better tolerance (26.5% reduction). In both seasons, superior lines generally withstand drought better than other lines, and RILs 33 and 41 showed low yield declines across years (Supp. Table 2).

### Variability, heritability and genetic advance in RILs

The estimates for coefficients of variability, heritability, and genetic advance are shown in Table 2. The results of the variability analysis indicate that the observed PCV values were generally higher than their corresponding GCV values for all traits considered. Traits were categorized by genetic variability into high (> 20%), moderate (10–20%), and low (< 10%) groups. Under well-watered conditions, PH, BY, and GY consistently showed high PCV and GCV values (above 20%) across both years, highlighting a strong genetic basis for yield improvement. Conversely, SPAD, TGW, and HI exhibited low variation (below 10%) (Table 2). Under drought stress, the pattern of variability was trait-dependent. GY maintained high genetic variability, with GCV reaching 24.23%, indicating good potential for direct selection. BY also showed high variation in 2020. However, HI demonstrated very low genetic variability (GCV of 4.07%), while TGW showed consistently low variation (PCV < 10%) across drought seasons (Table 2).

The heritability estimates for SPAD, PH, and TGW were found to be higher under drought conditions compared to well-watered conditions in both years. This trend was also observed for HI, BY, and GY in 2020. The heritability estimates for these traits ranged from 23.78% for SPAD to 95.59% for PH under well-watered conditions. However, under drought stress, the heritability estimates ranged from 15.47% for HI to 97.38% for PH. This suggests that genetic factors play a significant role in determining the performance of these traits under different environmental conditions (Table 2). The highest heritability over all environments was found for RWC and PH with values of 91.19% and 79.59%, respectively. On the other hand, SPAD exhibited the lowest heritability at 18.79% (Table 2).

The potential for genetic improvement, measured as genetic advance (GA), varied significantly among traits and between environmental conditions, showing higher importance for selection. Under well-watered conditions, BY showed the highest GA (43,198.92). In contrast, physiological traits such as RWC exhibited very low GA (13.40). This pattern held under drought stress, where BY again showed the highest GA (37,066.37), though moderately reduced, while RWC GA remained very low (12.25) (Table 2). When expressed as a percentage of the mean (GAM) to standardize comparison, GY itself appeared as the most responsive trait. Its GAM was very high under well-watered conditions (4,275) and increased further under drought stress (4,783.58). Conversely, HI showed the lowest relative advance in both conditions. These high GAM values for GY are supported by high heritability estimates for GY and TGW under optimal conditions (Table 2).

### Genotype × environment interaction for grain yield

The significant G × E interaction observed for grain yield was further dissected by examining the ranking consistency of top-performing RILs across environments (Supp. Table 3). The top 10 RILs based on overall mean yield showed a continuum of stability patterns. RILs 41 and 101 demonstrated high rank consistency with SD values of 2.9 and 5.0, respectively, indicating non-crossover interaction and broad adaptability across both well-watered and drought conditions. In contrast, RILs 82 and 30 exhibited low rank consistency (SD = 34.7 and 32.8), showing strong crossover interaction where performance was highly environment-specific. RIL 12, while having the highest overall mean yield (445.1), showed moderate rank consistency (SD = 15.2), excelling under well-watered conditions but showing reduced performance under drought stress in 2021.

The analysis of variance components revealed a clear hierarchy in the genetic consistency of key agronomic traits across years. PH and RWC demonstrated the highest levels of genetic predictability, with consistency ratios of 0.958 and 1.000, respectively (Supp. Table 4). TGW exhibited moderate genetic consistency with a ratio of 0.557. In contrast, the yield-related traits including GY and BY showed considerably lower ratios of 0.254 and 0.162. The traits most susceptible to annual environmental variation were the SPAD and HI, with the lowest stability ratios of 0.151 and 0.064, respectively (Supp. Table 4).

### Association between traits under well-watered and drought stress conditions

A correlation matrix was created to investigate the relationship between morpho-physiological and yield-related traits, as depicted in.

The analysis revealed positive relationship between BY and GY, which was highly significant across both growing seasons and water regimes. This correlation was especially strong under drought stress in the 2020–2021 season (*r* = 0.92, *P* < 0.0001). Furthermore, RWC appeared as a key physiological trait, showing a strong positive correlation with both BY and GY in the first season under well-watered (*r* = 0.75 and *r* = 0.86, respectively) (Fig. 2a) and drought (*r* = 0.81 and *r* = 0.71, respectively) conditions (*P* < 0.0001) (Fig. 2b). In the 2021–2022 season, HI also showed a significant positive correlation with GY under both water regimes (*r* = 0.66, *P* < 0.0001).

**Fig. 3:**
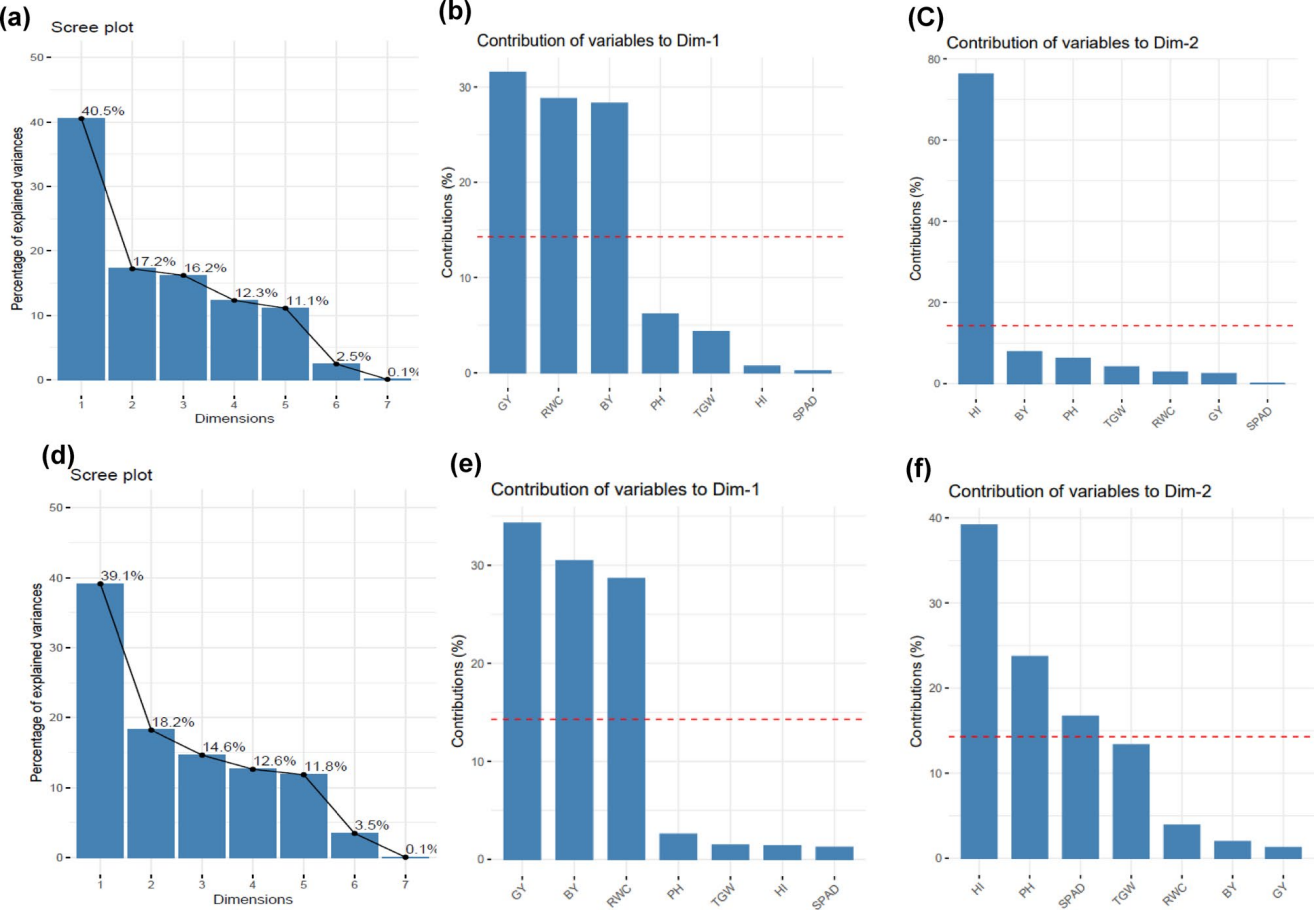
(**a**–**c**) Principal component analysis of the morpho-physiological and yield related traits under well-watered (WW) condition across two years (2020–2021 and 2021). (**a**) A scree plot for explained variance for the seven Principal Components (PCs) from the PCA analysis. The X-axis displays the PC and the Y-axis on the left shows percentage of variance explained. (**b**) Contribution of variables to first PC and (**c**) second PC under WW. (**d**–**f**) Principal component analysis of the morpho-physiological and yield related traits under drought stress (DS) condition. (**d**) A scree plot for explained variance for the seven PCs from the PCA analysis. (**e**) Contribution of variables to first PC and (**f**) second PC under DS.

**Fig. 4:**
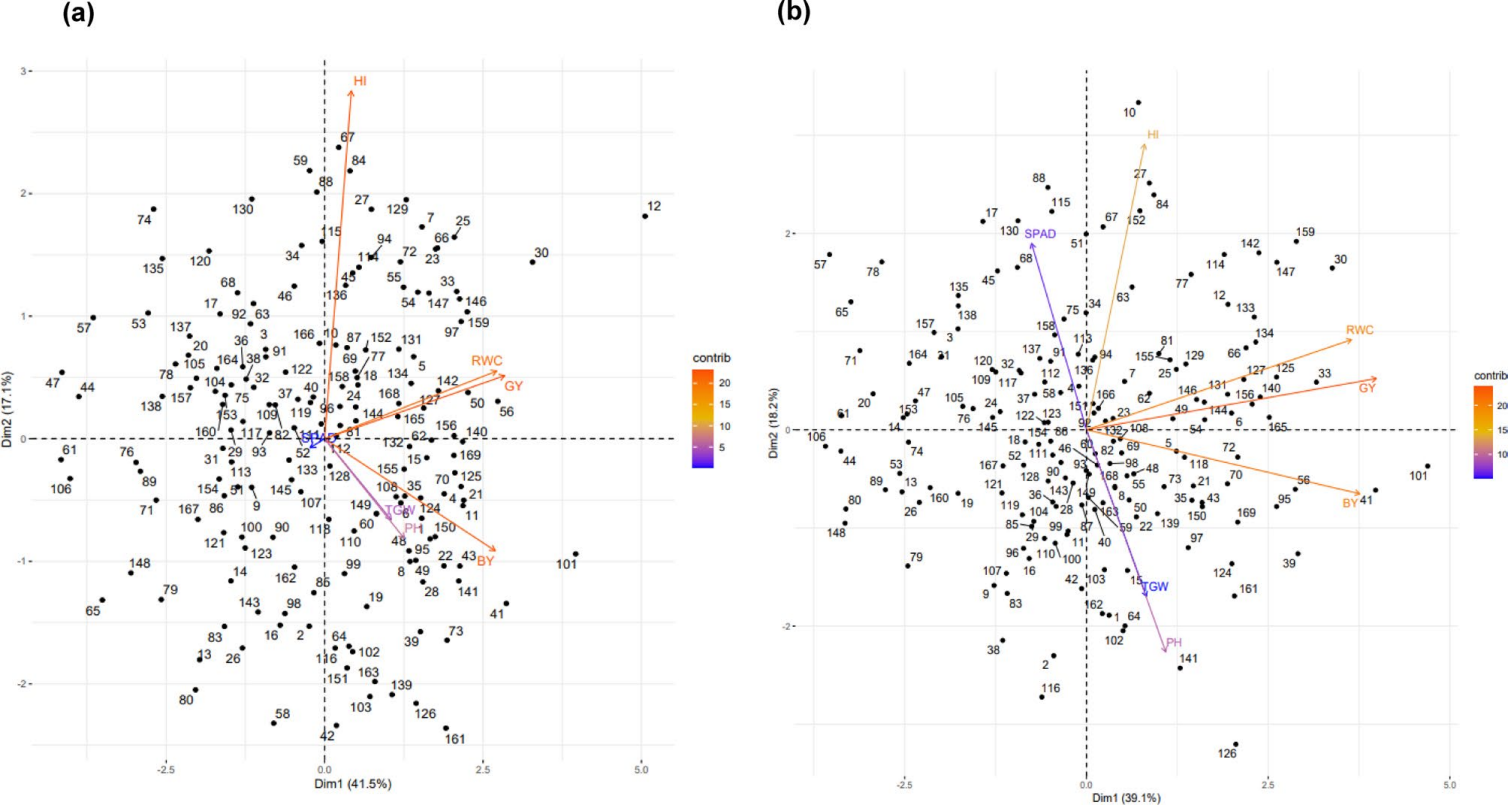
Principal component analysis (PCA) plot of the morpho-physiological and yield related traits in 167 wheat recombinant inbred lines (RILs) along with their two parents under two irrigation regimes, well-watered (WW) and drought stress (DS) conditions in two growing seasons. (**a**) PCA biplot of traits in the wheat accessions under WW condition. (**b**) PCA biplot of traits in the wheat accessions under DS condition.

The PCA was conducted to investigate the relationship patterns between morpho-physiological and yield-related traits. Theoretically, seven principal components (PCs) could be derived, but only those with significant contributions to the total variation were selected for further analysis. The scree plot illustrated the explained variance for the seven PCs under well-watered conditions (Fig. 3a). In the well-watered condition, five PCs with eigenvalues greater than 1 accounted for 97.3% of the variability in the dataset (Supp. Table 5). The first two PCs, PC1 and PC2, which collectively explained 57.7% of the total trait variation, were chosen for biplot analysis (Fig. 4a). PC1, associated with GY, RWC, and BY parameters, explained 40.5% of the total variance (Fig. 3b), while PC2 (HI) contributed approximately 17.2% to the total variance (Fig. 3c). The PCA results confirmed the positive correlations between RWC and GY, as initially identified through simple correlation analysis (Fig. 4a). Additionally, a strong association was observed between PH, RWC, and BY under well-watered conditions.

During periods of drought stress, five PCs with eigenvalues greater than 1 accounted for 96.3% of the variability in the dataset (Fig. 3d, Supp. Table 5). The first two PCs, which explain 57.3% of the total variation in traits, were utilized to analyze the relationship between traits in a biplot (Fig. 4b). Specifically, GY, BY, and RWC were identified as the most significant contributors to PC1 (Fig. 3e), while HI, PH, and SPAD values, with positive coefficients, were the most representative variables for PC2 (Fig. 3f). In the PCA biplot, a strong association was observed between RWC, GY, and BY under drought stress conditions (Fig. 3b). Furthermore, a significant correlation was found between TGW and PH, while TGW and PH showed a less pronounced correlation with HI.

After conducting PCA with the DTC of all traits, a comprehensive index was created using the first three PCs. This index was then applied in membership function analysis to evaluate 167 RILs along with their two parents over a span of two years, as detailed in Tables 3 and 4. The RILs were clearly differentiated based on the magnitude of their evaluation value (*D*). RIL 133 exhibited the highest *D* value (*D* = 0.751), followed by RILs 47 (*D* = 0.706) and 58 (*D* = 0.684). Conversely, RIL 3 (*D* = 0.207) and RIL 11 (*D* = 0.207) displayed the lowest *D* values in 2020. Among the RILs, 59 showed a *D* value greater than 0.5, indicating moderate drought tolerance within our wheat germplasm. Additionally, 100 RILs with *D* values ranging from 0.3 to 0.5 were considered moderately sensitive to drought. Ten specific RILs, including RILs 3, 11, 12, 18–20, 24, 45, 80, and 153, exhibited a *D* value below 0.3, indicating a high sensitivity to drought stress (Table 3). In 2021, RIL 33 demonstrated the highest *D* value (0.860), indicating a high level of drought tolerance. Following closely behind were RILs 13, 38, 28, 52, 65, 74, and 128, all with *D* values of ≥ 0.7 (Table 4). Conversely, RILs 58, 157, 153, 149, 156, and 87 were identified as the most sensitive to drought, with *D* values of ≤ 0.3.

**Table 3:** Comprehensive performance analysis of the weight, degree of membership (*U*_*i*_), and comprehensive evaluation value (*D*) for all investigated traits of the 167 wheat recombinant inbred lines (RILs) along with their two parents based on drought tolerance coefficient (DC) in 2020–2021.

Code	*Ui* value			*D* value	Code	*Ui* value			*D* value	Code	*Ui* value			*D* value
	U1	U2	U3			U1	U2	U3			U1	U2	U3	
G01	0.281	0.413	0.309	0.327	G58	0.403	0.933	0.897	0.684	G115	0.373	0.440	0.434	0.408
G02	0.369	0.490	0.601	0.463	G59	0.784	0.000	0.753	0.546	G116	0.353	0.233	0.398	0.329
G03	0.367	0.142	0.000	0.207	G60	0.276	0.502	0.572	0.418	G117	0.572	0.395	0.167	0.416
G04	0.082	0.460	0.650	0.338	G61	0.304	0.678	0.389	0.435	G118	0.682	0.487	0.637	0.613
G05	0.482	0.451	0.472	0.470	G62	0.366	0.192	0.587	0.372	G119	0.439	0.207	0.326	0.342
G06	0.546	0.563	1.000	0.667	G63	0.899	0.308	0.592	0.646	G120	0.549	0.378	0.143	0.394
G07	0.322	0.096	0.553	0.314	G64	0.563	0.362	0.754	0.552	G121	0.576	0.368	0.371	0.462
G08	0.294	0.550	0.287	0.367	G65	0.195	1.000	0.455	0.497	G122	0.596	0.132	0.595	0.459
G09	0.474	0.292	0.394	0.400	G66	0.553	0.367	0.390	0.457	G123	0.686	0.403	0.252	0.491
G10	0.530	0.740	0.167	0.498	G67	0.495	0.190	0.400	0.381	G124	0.646	0.443	0.446	0.535
G11	0.136	0.349	0.172	0.207	G68	0.429	0.549	0.678	0.528	G125	0.629	0.404	0.250	0.465
G12	0.170	0.385	0.244	0.252	G69	0.536	0.222	0.575	0.453	G126	0.651	0.302	0.494	0.508
G13	0.139	0.852	0.562	0.456	G70	0.465	0.509	0.517	0.491	G127	0.615	0.366	0.451	0.500
G14	0.098	0.551	0.846	0.422	G71	0.253	0.562	0.402	0.382	G128	0.429	0.348	0.084	0.316
G15	0.327	0.165	0.566	0.340	G72	0.775	0.324	0.162	0.485	G129	0.547	0.351	0.331	0.434
G16	0.423	0.374	0.510	0.430	G73	0.332	0.500	0.303	0.374	G130	0.520	0.294	0.355	0.411
G17	0.629	0.532	0.042	0.450	G74	0.503	0.180	0.378	0.376	G131	0.681	0.429	0.443	0.546
G18	0.293	0.528	0.046	0.298	G75	0.645	0.499	0.439	0.549	G132	0.393	0.367	0.462	0.403
G19	0.000	0.527	0.381	0.252	G76	0.791	0.481	0.532	0.633	G133	1.000	0.795	0.267	0.751
G20	0.222	0.287	0.281	0.256	G77	0.657	0.505	0.748	0.635	G134	0.650	0.673	0.170	0.533
G21	0.352	0.591	0.742	0.522	G78	0.228	0.422	0.655	0.394	G135	0.608	0.352	0.610	0.533
G22	0.212	0.306	0.952	0.429	G79	0.403	0.447	0.399	0.414	G136	0.464	0.343	0.324	0.392
G23	0.366	0.184	0.416	0.325	G80	0.128	0.356	0.392	0.262	G137	0.874	0.267	0.587	0.622
G24	0.158	0.454	0.288	0.278	G81	0.656	0.236	0.465	0.483	G138	0.468	0.480	0.667	0.522
G25	0.313	0.504	0.295	0.364	G82	0.729	0.548	0.357	0.580	G139	0.495	0.613	0.542	0.541
G26	0.284	0.551	0.212	0.344	G83	0.507	0.541	0.368	0.481	G140	0.551	0.505	0.481	0.519
G27	0.522	0.358	0.287	0.413	G84	0.726	0.300	0.177	0.460	G141	0.447	0.328	0.321	0.379
G28	0.249	0.475	0.351	0.341	G85	0.327	0.515	0.209	0.352	G142	0.627	0.545	0.249	0.506
G29	0.577	0.349	0.799	0.566	G86	0.750	0.565	0.303	0.581	G143	0.675	0.550	0.309	0.544
G30	0.482	0.538	0.523	0.508	G87	0.404	0.057	0.795	0.402	G144	0.620	0.368	0.941	0.628
G31	0.295	0.324	0.423	0.336	G88	0.395	0.384	0.233	0.350	G145	0.271	0.613	0.436	0.413
G32	0.525	0.489	0.503	0.508	G89	0.342	0.377	0.290	0.338	G146	0.488	0.552	0.165	0.423
G33	0.691	0.396	0.628	0.588	G90	0.771	0.197	0.830	0.617	G147	0.711	0.552	0.181	0.528
G34	0.593	0.406	0.218	0.442	G91	0.668	0.232	0.115	0.398	G148	0.364	0.323	0.194	0.308
G35	0.487	0.538	0.494	0.503	G92	0.746	0.423	0.570	0.606	G149	0.469	0.168	0.369	0.355
G36	0.645	0.464	0.531	0.562	G93	0.733	0.498	0.078	0.496	G150	0.479	0.424	0.382	0.438
G37	0.411	0.448	0.441	0.429	G94	0.540	0.121	0.250	0.342	G151	0.348	0.769	0.554	0.524
G38	0.440	0.222	0.606	0.418	G95	0.724	0.441	0.310	0.534	G152	0.559	0.519	0.153	0.443
G39	0.733	0.659	0.503	0.651	G96	0.285	0.239	0.421	0.306	G153	0.239	0.380	0.305	0.297
G40	0.438	0.476	0.682	0.511	G97	0.392	0.316	0.376	0.366	G154	0.634	0.483	0.550	0.568
G41	0.600	0.577	0.848	0.656	G98	0.701	0.697	0.340	0.607	G155	0.541	0.543	0.199	0.453
G42	0.402	0.755	0.662	0.571	G99	0.530	0.155	0.343	0.372	G156	0.556	0.517	0.421	0.510
G43	0.491	0.456	0.213	0.409	G100	0.707	0.497	0.284	0.537	G157	0.493	0.306	0.245	0.374
G44	0.492	0.386	0.363	0.427	G101	0.618	0.753	0.301	0.576	G158	0.354	0.203	0.724	0.404
G45	0.144	0.317	0.468	0.278	G102	0.610	0.506	0.544	0.562	G159	0.614	0.419	0.367	0.493
G46	0.607	0.385	0.601	0.540	G103	0.404	0.630	0.588	0.517	G160	0.242	0.314	0.531	0.337
G47	0.910	0.513	0.572	0.706	G104	0.892	0.277	0.732	0.670	G161	0.520	0.592	0.427	0.517
G48	0.406	0.370	0.630	0.452	G105	0.429	0.283	0.239	0.337	G162	0.662	0.335	0.505	0.525
G49	0.421	0.605	0.698	0.545	G106	0.491	0.207	0.455	0.398	G163	0.546	0.499	0.180	0.438
G50	0.277	0.280	0.409	0.312	G107	0.285	0.388	0.395	0.343	G164	0.393	0.197	0.271	0.304
G51	0.682	0.725	0.471	0.640	G108	0.402	0.493	0.434	0.437	G165	0.739	0.460	0.498	0.595
G52	0.381	0.446	0.393	0.403	G109	0.434	0.267	0.317	0.354	G166	0.511	0.364	0.427	0.446
G53	0.441	0.194	0.537	0.393	G110	0.367	0.250	0.259	0.305	G167	0.623	0.380	0.268	0.460
G54	0.528	0.231	0.624	0.465	G111	0.403	0.394	0.380	0.394	G168	0.392	0.388	0.507	0.420
G55	0.447	0.276	0.432	0.393	G112	0.391	0.269	0.512	0.386	G169	0.543	0.535	0.404	0.505
G56	0.515	0.548	0.419	0.499	G113	0.790	0.345	0.407	0.561					
G57	0.297	0.561	0.542	0.437	G114	0.664	0.598	0.383	0.572					
Weight	0.450					0.293					0.256

**Table 4:** Comprehensive performance analysis of the weight, degree of membership (*U*_*i*_), and comprehensive evaluation value (*D*) for all investigated traits of the 167 wheat recombinant inbred lines (RILs) along with their two parents based on drought tolerance coefficient (DC) in 2021–2022.

Code	*Ui* value			*D* value	Code	*Ui* value			*D* value	Code	*Ui* value			*D* value
	U1	U2	U3			U1	U2	U3			U1	U2	U3	
G01	0.696	0.539	0.474	0.585	G58	0.090	0.309	0.394	0.242	G115	0.362	0.491	0.155	0.345
G02	0.893	0.407	0.434	0.613	G59	0.730	0.562	0.404	0.587	G116	0.665	0.747	0.477	0.639
G03	0.853	0.660	0.108	0.587	G60	0.560	0.404	0.438	0.476	G117	0.348	0.515	0.473	0.434
G04	0.663	0.321	0.283	0.450	G61	0.467	0.491	0.568	0.501	G118	0.402	0.401	0.691	0.480
G05	0.500	0.373	0.073	0.343	G62	0.717	0.346	0.851	0.635	G119	0.686	0.918	0.402	0.680
G06	0.611	0.539	0.345	0.514	G63	0.339	0.438	0.355	0.374	G120	0.169	0.855	0.237	0.403
G07	0.685	0.451	0.577	0.581	G64	0.219	0.621	0.193	0.338	G121	0.210	0.636	0.401	0.396
G08	0.567	0.571	0.420	0.527	G65	0.852	0.834	0.407	0.723	G122	0.632	0.812	0.217	0.574
G09	0.856	0.551	0.581	0.683	G66	0.617	0.850	0.576	0.678	G123	0.594	0.610	0.606	0.601
G10	0.187	0.750	0.416	0.427	G67	0.252	0.717	0.376	0.432	G124	0.677	0.600	0.554	0.618
G11	0.854	0.541	0.499	0.657	G68	0.539	0.528	0.405	0.498	G125	0.350	0.585	0.338	0.420
G12	0.499	0.578	0.370	0.488	G69	0.555	0.716	0.622	0.623	G126	0.636	0.652	0.446	0.588
G13	0.965	0.751	0.259	0.703	G70	0.526	0.381	0.309	0.420	G127	0.462	0.640	0.379	0.494
G14	0.741	0.197	0.000	0.366	G71	0.315	0.383	0.329	0.340	G128	0.758	0.908	0.452	0.720
G15	0.930	0.085	0.445	0.529	G72	0.429	0.679	0.571	0.545	G129	0.413	0.846	0.451	0.559
G16	0.534	0.538	0.438	0.508	G73	0.417	0.726	0.502	0.537	G130	0.552	0.431	0.481	0.493
G17	0.000	0.489	0.566	0.309	G74	0.760	0.691	0.652	0.707	G131	0.526	0.485	0.702	0.560
G18	0.423	0.528	0.176	0.388	G75	0.625	0.811	0.418	0.626	G132	0.405	0.638	0.136	0.404
G19	0.812	0.581	0.109	0.546	G76	0.599	1.000	0.231	0.624	G133	0.394	0.697	0.530	0.526
G20	0.811	0.487	0.313	0.572	G77	0.486	0.622	0.730	0.594	G134	0.273	0.506	0.550	0.422
G21	0.515	0.311	0.335	0.401	G78	0.607	0.369	0.116	0.397	G135	0.691	0.553	0.425	0.573
G22	0.360	0.239	0.484	0.355	G79	0.506	0.859	0.163	0.522	G136	0.680	0.546	0.304	0.534
G23	0.501	0.234	0.146	0.319	G80	0.393	0.226	0.295	0.313	G137	0.143	0.571	0.411	0.351
G24	0.642	0.515	0.062	0.442	G81	0.544	0.714	0.543	0.596	G138	0.568	0.671	0.562	0.598
G25	0.517	0.746	0.568	0.602	G82	0.392	0.632	0.190	0.411	G139	0.441	0.688	0.332	0.488
G26	0.321	0.502	0.288	0.368	G83	0.601	0.820	0.281	0.582	G140	0.337	0.671	0.656	0.529
G27	0.379	0.608	0.234	0.411	G84	0.618	0.702	0.341	0.568	G141	0.517	0.604	0.319	0.489
G28	1.000	0.572	0.443	0.711	G85	0.300	0.555	0.385	0.403	G142	0.354	0.513	0.320	0.394
G29	0.677	0.440	0.609	0.583	G86	0.398	0.576	0.523	0.487	G143	0.541	0.778	0.493	0.601
G30	0.724	0.544	0.292	0.548	G87	0.318	0.217	0.370	0.300	G144	0.615	0.573	0.722	0.629
G31	0.581	0.466	0.284	0.462	G88	0.682	0.831	0.236	0.606	G145	0.836	0.437	0.218	0.540
G32	0.647	0.710	0.335	0.580	G89	0.571	0.728	0.514	0.604	G146	0.683	0.698	0.180	0.549
G33	0.961	0.612	1.000	0.860	G90	0.376	0.460	0.335	0.390	G147	0.046	0.636	0.434	0.338
G34	0.711	0.635	0.290	0.571	G91	0.322	0.677	0.367	0.445	G148	0.592	0.640	0.071	0.464
G35	0.820	0.487	0.519	0.631	G92	0.057	0.794	0.740	0.475	G149	0.419	0.000	0.379	0.275
G36	0.594	0.266	0.376	0.430	G93	0.539	0.746	0.435	0.575	G150	0.347	0.554	0.295	0.397
G37	0.827	0.474	0.353	0.585	G94	0.669	0.687	0.163	0.536	G151	0.745	0.731	0.257	0.606
G38	0.935	0.430	0.794	0.736	G95	0.379	0.392	0.484	0.411	G152	0.489	0.557	0.327	0.465
G39	0.207	0.631	0.470	0.412	G96	0.658	0.848	0.367	0.637	G153	0.136	0.584	0.048	0.253
G40	0.869	0.413	0.284	0.564	G97	0.400	0.928	0.599	0.620	G154	0.345	0.434	0.539	0.425
G41	0.655	0.402	0.501	0.532	G98	0.248	0.565	0.457	0.405	G155	0.711	0.867	0.068	0.583
G42	0.607	0.465	0.449	0.518	G99	0.378	0.601	0.397	0.453	G156	0.050	0.547	0.321	0.280
G43	0.705	0.340	0.123	0.430	G100	0.353	0.911	0.492	0.566	G157	0.199	0.297	0.251	0.244
G44	0.616	0.506	0.273	0.487	G101	0.357	0.487	0.706	0.493	G158	0.607	0.263	0.539	0.479
G45	0.817	0.749	0.212	0.629	G102	0.729	0.591	0.637	0.659	G159	0.537	0.682	0.148	0.475
G46	0.756	0.554	0.421	0.599	G103	0.629	0.694	0.243	0.543	G160	0.256	0.767	0.108	0.376
G47	0.781	0.218	0.260	0.460	G104	0.612	0.615	0.739	0.647	G161	0.369	0.624	0.567	0.503
G48	0.565	0.557	0.282	0.484	G105	0.489	0.431	0.404	0.446	G162	0.634	0.740	0.199	0.547
G49	0.703	0.339	0.257	0.465	G106	0.810	0.785	0.389	0.686	G163	0.384	0.760	0.262	0.468
G50	0.616	0.556	0.156	0.470	G107	0.653	0.541	0.746	0.642	G164	0.684	0.499	0.103	0.466
G51	0.439	0.271	0.549	0.415	G108	0.677	0.628	0.385	0.581	G165	0.327	0.450	0.452	0.399
G52	0.927	0.704	0.360	0.700	G109	0.360	0.589	0.418	0.447	G166	0.260	0.607	0.406	0.409
G53	0.665	0.450	0.349	0.510	G110	0.490	0.326	0.224	0.365	G167	0.353	0.551	0.537	0.465
G54	0.573	0.677	0.302	0.530	G111	0.501	0.588	0.203	0.446	G168	0.370	0.719	0.057	0.394
G55	0.729	0.486	0.599	0.616	G112	0.536	0.346	0.356	0.426	G169	0.403	0.479	0.439	0.436
G56	0.251	0.593	0.365	0.389	G113	0.381	0.592	0.448	0.465					
G57	0.599	0.631	0.190	0.496	G114	0.477	0.600	0.882	0.626					
Weight	0.410					0.315					0.273			

### Screening RILs for drought tolerance/susceptibility indices

Five stress-tolerant indices were calculated for 167 RILs along with their two parents under both well-watered and drought stress conditions over two years. This analysis utilized data on morpho-physiological and yield-related traits from two years (Supp. Tables 6–19). Based on the STI indices for morpho-physiological and related traits over the two-year period, RILs 101, 147, 56, 41, 70, and 159 exhibited the highest values for RWC and BY in 2020 (Supp. Figure 2). Notably, RIL 101 achieved the highest grain yield under stress conditions (Ys) with a value of 370 kg m^−2^, followed by RIL 159 (338.33 kg m^−2^) and RIL 41 (337 kg m^−2^). Conversely, under well-watered conditions, RILs 12 (523 kg m^−2^), 30 (448.67 kg m^−2^), 101 (436.33 kg m^−2^), and 159 (421 kg m^−2^) demonstrated the highest grain yield. By utilizing stress-tolerant indices, a total of seven RILs (101, 159, 41, 33, 142, 66, and 95) showed excellence under stress conditions (Supp. Tables 6–19). These entries displayed significantly higher values for grain yield under drought stress conditions (Ys), STI, YSI, and RSI, while showing lower values for TOL and SSI. RILs such as 101, 12, 30, 159, 41, 33, and 142, which exhibited the highest STI values, were classified as tolerant RILs. In terms of TOL, RILs 90, 133, 104, 76, 47, 98, and 63 demonstrated the smallest yield difference (> 20) between well-watered and drought conditions. On the contrary, RILs 4, 7, 11, 12, 97, and 50 displayed the most significant differences, boasting the highest SSI values. These results indicate that RILs with elevated SSI values tend to demonstrate a substantial variance in yield under stressful versus non-stressful conditions. Additionally, after analyzing the YSI, RSI, and STI values for grain and biological yields, as well as the harvest index of wheat RILs in 2021, it was determined that RILs 2, 3, 15, 19, 22, 35–38, 40, 47, 49, and 51 exhibited superior performance in both scenarios (Supp. Tables 6–19).

### Performance of RILs relative to parental lines

Within the drought stress environment, the elite RILs demonstrated a consistent and significant superiority over their parental lines. The parents exhibited the expected contrasting patterns: Seri (RIL 168) showed moderate yield potential with considerable stress sensitivity (yield reductions of 27.8% and 38.5%), while Babax (RIL 169) displayed higher yield potential with better drought tolerance (reductions of 26.2% and 33.6%). In contrast, the elite RILs demonstrated clear transgressive segregation, outperforming both parents. In the first season, RILs like 101, 159, and 41 achieved higher yields under both conditions with lower percent reductions (e.g., 15.2% for RIL 101). This advantage was confirmed and even strengthened in the second season, which had a higher drought severity (DII = 0.27), where RILs 41 and 33 exhibited remarkable stability with reductions of only 15.2% and 10.9%, respectively, while RIL 95 recorded the highest overall yield potential (Supp. Tables 6–19).

### Identification of the best indices based on the correlation among indices and grain yield

Supp. Figure 1a illustrates the correlation coefficients between drought indices and grain yield over two years, both in well-watered and drought stress conditions. In the 2020–2021 period, statistically significant correlation coefficients (*P* < 0.0001) were identified for SSI, STI, YSI, RSI, and TOL. Particularly noteworthy was the positive and significant correlation between grain yield under drought stress (YS) and STI (*r* = 0.96, *P* < 0.0001). Additionally, YP exhibited a significantly positive correlation (*P* < 0.0001) with SSI (*r* = 0.62), STI (*r* = 0.96), and TOL (*r* = 0.62). YS also displayed a significantly positive relationship (*P* < 0.0001) with STI (*r* = 0.96), indicating that selecting based on STI could enhance yield under both well-watered and drought stress conditions. Conversely, negative and significant correlations were observed between SSI and YSI (*r* = -0.8) as well as RSI (*r* = − 0.8). Furthermore, SSI and TOL exhibited a positive and significant correlation with grain yield under non-stress conditions (YP) in 2020–2021. A similar pattern was noted in 2021–2022 (Supp. Figure 1b).

### Clustering wheat RILs based on drought tolerant indices regarding yield components

**Fig. 5:**
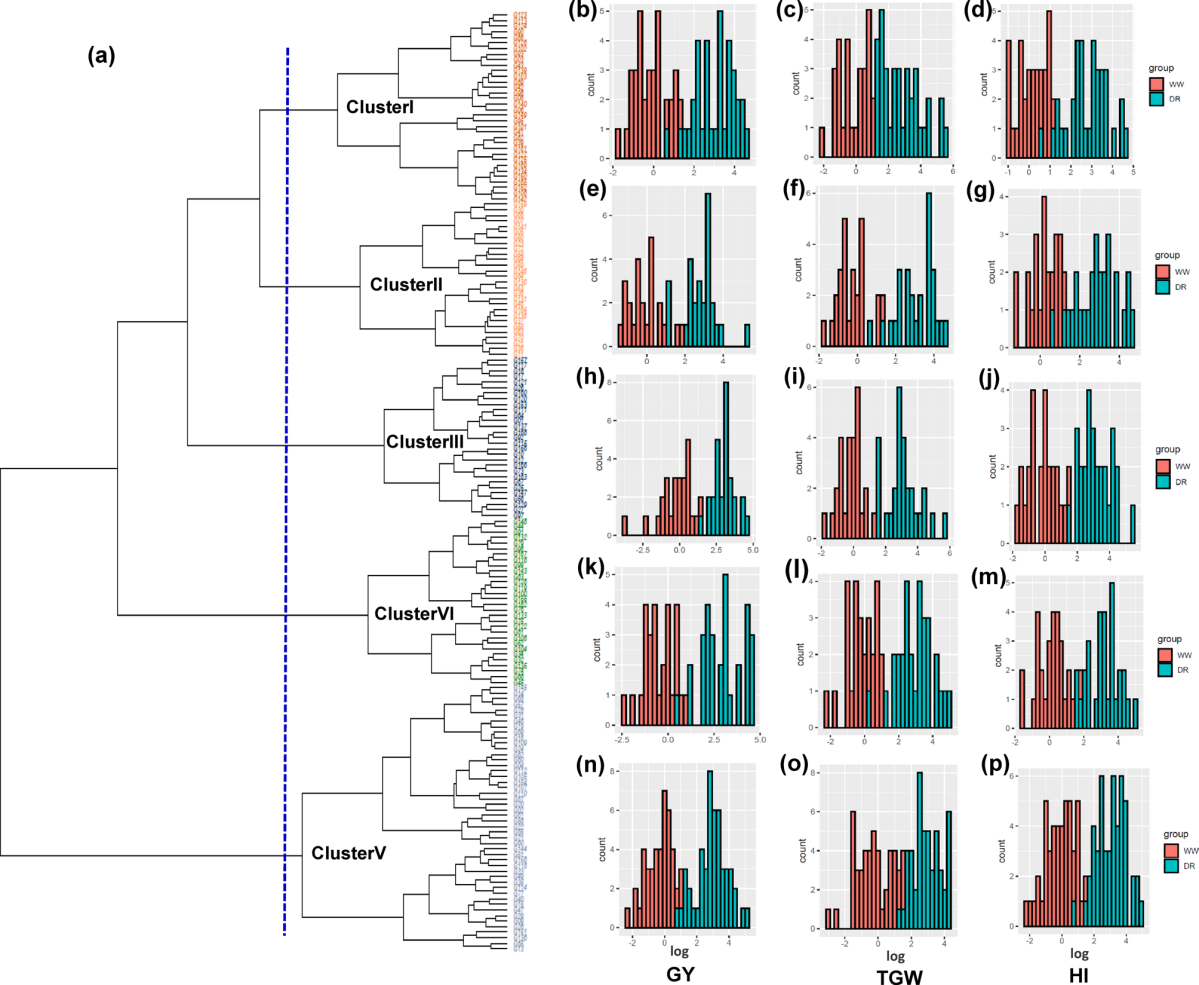
(**a**) Dendrogram from Ward's method analysis of 167 wheat recombinant inbred lines (RILs) along with their two parents based on drought tolerance indices, stress susceptibility index (SSI), stress tolerance index (STI), yield stability index (YSI), relative stress index (RSI), tolerance index (TOL) and yield components in the overall mean under non-stress and drought stress. (**b**–**f**) Histogram of yield component of wheat at well-watered (WW) VS drought stress (DR) condition. (**b**–**f**) Distribution of grain yield (GY) (**b**), thousand grain weight (TGW) (**c**), and harvest index (HI) (**d**) displayed for the cluster I of 35 wheat lines. Distribution of GY (**e**), TGW (**f**), and HI (**g**) displayed for the cluster II of 27 wheat lines. Distribution of GY (**h**), TGW (**i**), and HI (**j**) displayed for the cluster III of 28 wheat lines. Distribution of GY (**k**), TGW (**l**), and HI (**m**) displayed for the cluster VI of 30 wheat lines. Distribution of GY (**n**), TGW (**o**), and HI (**p**) displayed for the cluster V of 49 wheat lines.

The analysis of clusters based on five drought tolerance indices and morpho-physiological traits under both drought and well-watered conditions resulted in the creation of a dendrogram that classified the 167 RILs along with their two parents into five main clusters (Fig. 5). The largest cluster, cluster V, consisted of 49 RILs (28.99%), followed by cluster I with 35 RILs (20.71%), and the smallest group, cluster II, which included 27 RILs (15.97%). RILs grouped in cluster I for both years exhibited high RSI, STI, and moderate TOL and SSI indices (Supp. Tables 10–19). Additionally, members of this cluster demonstrated the highest grain yields compared to other clusters, ranging from 296 to 474 kg m^−2^ under well-watered conditions and 233–367 kg m^−2^ under drought stress. These RILs were identified as highly tolerant to drought stress.

In cluster II, grain yields ranged from 281 to 412.33 kg m^−2^ under well-watered conditions and 212.17–320.84 kg m^−2^ under drought conditions (Fig. 5b–g). Subgroups within this cluster were identified, including RILs with moderate to high grain yields and high consistent performance under drought stress conditions, such as RILs 37, 38, 46, 59, and 84 from cluster II. Cluster III consisted of 28 RILs that exhibited moderate to high performance under non-stress conditions, as well as high values of SSI and TOL indices (Supp. Tables 10–19). These RILs were identified as susceptible to drought but suitable for non-stress conditions exclusively. In terms of grain yield, they ranged from 289 to 463.165 kg m^−2^ in well-watered conditions and from 174.67 kg m^−2^ to 296 in drought stress conditions within cluster III (Fig. 5h–j). Following Cluster I, Cluster III had the highest grain yield under well-watered conditions. Interestingly, RILs 12 and 156 within this cluster showed poor yield performance under drought stress conditions. On the other hand, RILs grouped in Cluster IV demonstrated moderate to high levels of drought tolerance indices, including STI, RSI, and YSI (Supp. Tables 10–19). The grain yield of RILs in Cluster IV ranged from 233.66 to 373.5 kg m^−2^ in well-watered conditions and 181–290.83 kg m^−2^ in drought stress (Fig. 5k–m). Furthermore, RILs in Cluster IV exhibited moderate performance under stressed environments in this study (Fig. 5n–p). This pattern was also evident in Cluster V (Fig. 5n–p). RILs in Cluster V showed high drought indices such as RSI and TOL, and were categorized as semi-sensitive to drought with low consistent performance (Supp. Tables 10–19). Cluster III outperformed other clusters in terms of grain yield under optimal conditions, while Clusters IV and V showed varying levels of drought tolerance. RILs in Cluster IV displayed a wide range of grain yield values under different conditions, indicating their adaptability to stress. Conversely, RILs in Cluster V exhibited high sensitivity to drought, highlighting the importance of genetic variability in breeding programs.

### The MGIDI index speed up the screening of drought-tolerant RILs based on traits and drought indices

**Fig. 6:**
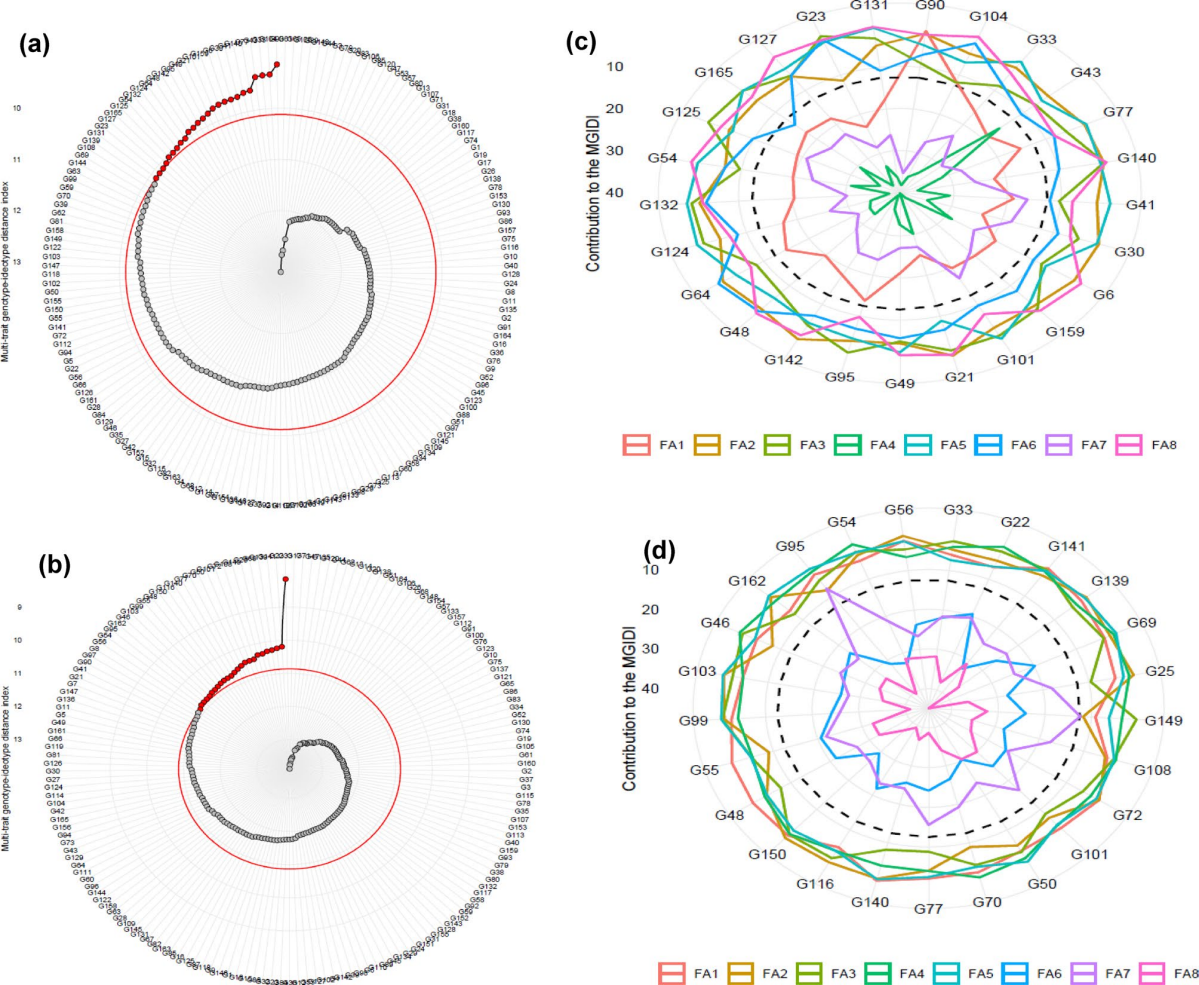
(**a** and **b**) Wheat recombinant inbred lines (RILs) ranking in ascending order for the MGIDI index across two years (**a**) in 2020–2021 and (**b**) in 2021–2022. The selected lines based on this index are shown in red. The central red circle represents the cutpoint according to the selection pressure. (**c** and **d**) Strengths and weaknesses view of the selected wheat RILs across two years (**c**) in 2020–2021 and (**d**) in 2021–2022 is shown as the proportion of each factor (FA) on the computed MGIDI index. The smallest the proportion explained by a factor (closer to the external edge), the closer the traits within that factor are to the ideotype. The dashed line indicates the theoretical value if all factors had contributed equally.

In order to determine the top-performing RILs, we utilized MGIDI values to rank the accessions based on various indices. With a selection pressure of 10% and MGIDI values from the first year, a total of 25 RILs were selected under drought stress conditions in the initial year of the experiment (Fig. 6a). In the subsequent year, an additional 25 RILs were chosen under drought stress. Notably, RILs 23, 127, 131, and 165 were found to be very close to the cut-off point in the 2020–2021 season, while RILs 8, 46, 54, 56, 95, 103, and 16 were near the cut-off point in the 2021–2022 growing season (Fig. 6b), suggesting potential characteristics worthy of further investigation. The selected RILs under drought stress conditions exhibited desirable selection gains (SGs) for the mean performance of all traits over the two years. Specifically, in 2020–2021, there were higher positive SG percentages for the drought indices (RSI and YSI) for GY, as well as the STI index based on RWC and TGW, and TOL for TGW (Table 5). Negative gain percentages were observed for the YSI and RSI for PH, and SSI for RWC in 2020–2021. In 2021–2022, high SG percentages were identified for the STI for PH (33.2%), GY (10.8%), RWC (9.13%), as well as indices based on GY including TOL (Table 6). However, higher negative gains were observed for indices based on SPAD, including TOL at -4.25% and SSI at -3.98% (Table 6).

**Table 5:** Linkage of factors to traits, mean of the original population (*Xo*), mean of the selected genotype (*Xs*), selection differential (SD), selection differential percentage (SD%), selection gains (SG), selection gain percentage (SG%), objectives, and goal based on the multi-trait genotype–ideotype distance index (MGIDI) in 2020–2021.

Trait	Factor	Mean performance						Objective	Goal
		*Xo*	*Xs*	SD	SD%	SG	SG%		
SSI-GY	FA1	0.99	0.97	− 2.03	− 2.03	− 0.00617	− 0.618	Increase	0
YSI-GY	FA1	0.76	0.76	2.92E− 14	2.92E− 14	5.81E− 31	7.64E− 29	Increase	100
RSI-GY	FA1	1.02	1.02	2.17E− 14	2.17E− 14	5.67E− 31	5.53E− 29	Increase	100
TOL-GY	FA1	73.5	72	− 2.06	− 2.06	− 0.46	− 0.626	Increase	0
SSI-BY	FA1	1	0.97	− 2.17	− 2.17	− 0.00878	− 0.878	Increase	0
YSI-BY	FA1	0.80	0.82	2	2	0.00327	0.406	Increase	100
RSI-BY	FA1	1.02	1.05	2	2	0.00416	0.406	Increase	100
TOL-BY	FA1	219	214	− 2.18	− 2.18	− 1.93	− 0.882	Increase	0
SSI-HI	FA2	0.99	0.86	− 13	− 13	− 0.0787	− 7.89	Increase	0
YSI-HI	FA2	0.95	0.96	1.1	1.1	0.00649	0.682	Increase	100
RSI-HI	FA2	1.01	1.02	1.1	1.1	0.00689	0.682	Increase	100
TOL-HI	FA2	1.61	1.27	− 21.1	− 21.1	− 0.206	− 12.8	Increase	0
SSI-TGW	FA3	0.99	1.03	3.24	3.24	0.0275	2.75	Increase	100
YSI-TGW	FA3	0.82	0.81	− 0.59	− 0.59	− 0.00409	− 0.496	Increase	0
RSI-TGW	FA3	1	0.99	− 0.59	− 0.59	− 0.00499	− 0.496	Increase	0
TOL-TGW	FA3	5.88	6.11	3.79	3.79	0.19	3.22	Increase	100
SSI-PH	FA4	1	1.07	6.87	6.87	0.00365	0.365	Increase	100
STI-PH	FA4	0.90	1.02	12.2	12.2	0.0506	5.58	Increase	100
YSI-PH	FA4	0.91	0.89	− 2.35	− 2.35	− 0.0116	− 1.27	Increase	0
RSI-PH	FA4	1.02	0.99	− 2.35	− 2.35	− 0.013	− 1.27	Increase	0
TOL-PH	FA4	10.5	11.3	7.53	7.53e + 0	0.0421	0.4	Increase	100
SSI-SPAD	FA5	0.99	1.01	0.891	0.891	0.00263	0.264	Increase	100
YSI-SPAD	FA5	0.69	0.68	− 0.796	− 0.796	− 0.00204	− 0.294	Increase	0
RSI-SPAD	FA5	1.02	1.02	− 0.796	− 0.796	− 0.00301	− 0.294	Increase	0
TOL-SPAD	FA5	14.1	14.2	0.956	0.956	0.0398	0.283	Increase	100
SSI-RWC	FA6	0.99	0.99	− 0.00926	− 0.00926	− 0.0000189	− 0.0019	Increase	0
YSI-RWC	FA6	0.73	0.74	0.405	0.405	0.00051	0.0692	Increase	100
RSI-RWC	FA6	1.02	1.03	0.405	0.405	0.000707	0.0692	Increase	100
TOL-RWC	FA6	0.16	0.16	− 0.0573	− 0.0573	− 0.0000199	− 0.0117	Increase	0
STI-GY	FA7	0.77	1.19	53	53	0.387	49.7	Increase	100
STI-BY	FA7	0.82	1.21	47	47	0.361	44.1	Increase	100
STI-RWC	FA7	0.74	0.89	21.3	21.3	0.126	17.1	Increase	100
STI-HI	FA8	0.95	0.98	3.93	3.93	0.0345	3.63	Increase	100
STI-TGW	FA8	0.82	0.84	2.2	2.2	0.0168	2.04	Increase	100
STI-SPAD	FA8	0.67	0.67	− 0.968	− 0.968	− 0.00331	− 0.487	Increase	0

*SSI* stress susceptibility index, *STI* stress tolerance index, *YSI* yield stability index, *RSI* relative stress index, *TOL* tolerance index, *GY* grain yield, *BY* biological yield, *HI* harvest index, *TGW* thousand grain weight, *PH* plant height, *SPAD* estimation of chlorophyll using SPAD, *RWC* relative water content.

**Table 6:** Linkage of factors to traits, mean of the original population (*Xo*), mean of the selected genotype (*Xs*), selection differential (SD), selection differential percentage (SD%), selection gains (SG), selection gain percentage (SG%), objectives, and goal based on the multi-trait genotype–ideotype distance index (MGIDI) in 2021–2022.

Trait	Factor	Mean performance						Objective	Goal
		*Xo*	*Xs*	SD	SD%	SG	SG%		
SSI-GY	FA1	0.85	0.92	0.06	7.79	0.04	4.7	Increase	100
YSI-GY	FA1	0.74	0.73	− 0.008	− 1.13	− 0.004	− 0.62	Increase	0
RSI-GY	FA1	1.08	1.07	− 0.01	− 1.13	− 0.006	− 0.62	Increase	0
TOL-GY	FA1	108	116	8.49	7.86	5.12	4.75	Increase	100
SSI-BY	FA1	0.92	1.03	0.10	10.9	0.03	3.45	Increase	100
YSI-BY	FA1	0.91	0.91	− 0.006	− 0.71	− 0.001	− 0.16	Increase	0
RSI-BY	FA1	1.03	1.02	− 0.007	− 0.71	− 0.001	− 0.16	Increase	0
TOL-BY	FA1	112	125	12.3	11	3.91	3.48	Increase	100
SSI-HI	FA2	0.84	0.84	− 0.001	− 0.12	− 5.7e− 05	− 0.006	Increase	0
YSI-HI	FA2	0.82	0.82	3.99e− 05	0.004	2.2e− 07	2.66e− 05	Increase	100
RSI-HI	FA2	1.05	1.05	5.08e− 05	0.004	2.8e− 07	2.66e− 05	Increase	100
TOL-HI	FA2	6.49	6.48	− 0.009	− 0.13	− 0.0005	− 0.007	Increase	0
SSI-SPAD	FA3	0.95	0.90	− 0.05	− 5.76	− 0.03	− 3.98	Increase	0
STI-SPAD	FA3	0.65	0.64	− 0.009	− 1.39	− 0.005	− 0.85	Increase	0
YSI-SPAD	FA3	0.67	0.68	0.01	1.59	0.007	1.11	Increase	100
RSI-SPAD	FA3	1.06	1.08	0.016	1.59	0.01	1.11	Increase	100
TOL-SPAD	FA3	15.1	14.1	− 0.92	− 6.14	− 0.63	− 4.25	Increase	0
SSI-RWC	FA4	1.01	1.01	0.001	0.13	0.0001	0.01	Increase	100
YSI-RWC	FA4	0.74	0.74	− 0.001	− 0.18	− 0.0002	− 0.03	Increase	0
RSI-RWC	FA4	0.98	0.98	− 0.001	− 0.18	− 0.0003	− 0.03	Increase	0
TOL-RWC	FA4	0.18	0.19	0.0016	0.87	0.0001	0.08	Increase	100
SSI-PH	FA5	1.02	1.01	− 0.007	− 0.69	− 0.001	− 0.16	Increase	0
YSI-PH	FA5	0.91	0.91	0.0009	0.10	7.26e− 05	0.007	Increase	100
RSI-PH	FA5	1	1	0.001	0.10	8.01e− 05	0.007	Increase	100
TOL-PH	FA5	9.1	9.03	− 0.06	− 0.76	− 0.01	− 0.18	Increase	0
SSI-TGW	FA6	0.99	0.96	− 0.03	− 3.08	− 0.01	− 1.82	Increase	0
YSI-TGW	FA6	0.79	0.80	0.006	0.78	0.0003	0.04	Increase	100
RSI-TGW	FA6	1.03	1.04	0.008	0.78	0.0005	0.04	Increase	100
TOL-TGW	FA6	8.38	8.09	− 0.28	− 3.45	− 0.17	− 2.04	Increase	0
STI-GY	FA7	0.71	0.80	0.09	12.7	0.07	10.8	Increase	100
STI-BY	FA7	0.91	0.93	0.02	2.61	0.01	1.51	Increase	100
STI-HI	FA7	0.78	0.80	0.01	2.27	0.004	0.51	Increase	100
STI-TGW	FA8	0.78	0.81	0.02	3.03	0.01	2.51	Increase	100
STI-PH	FA8	1.07	1.43	0.36	34	0.35	33.2	Increase	100
STI-RWC	FA8	0.73	0.81	0.08	11.9	0.06	9.13	Increase	100

*SSI* stress susceptibility index, *STI* stress tolerance index, *YSI* yield stability index, *RSI* relative stress index, *TOL* tolerance index, *GY* grain yield, *BY* biological yield, *HI* harvest index, *TGW* thousand grain weight, *PH* plant height, *SPAD* estimation of chlorophyll using SPAD, *RWC* relative water content.

### The strengths and weaknesses of the selected RILs based on MGIDI

The drought indices for morpho-physiological and yield traits were divided into eight factors (FA1, FA2, FA3, FA4, FA5, FA6, FA7, and FA8) over a span of two years, as shown in Tables 5 and 6. The analysis of screening RILs, based on the proportion of each factor to the MGIDI index of the RILs, is depicted in Fig. 6, d. In the first year, FA1 had the most significant impact on the MGIDI for RILs 64, 77, 90, and 95, indicating that these RILs exhibit higher biological and grain yields under drought conditions (Fig. 6c). Conversely, FA1 had the lowest influence on RILs 21, 23, and 30. FA2, which is associated with tolerance indices such as YSI, RSI, SSI, and TOL for harvest index, had the highest contribution for RILs 21, 30, and 64, while RIL 23 had the lowest contribution, suggesting high values of TOL (5.77%) and SSI (2.58%) for harvest index. FA3, linked to tolerance indices for TGW, showed the highest contribution for RIL 43. FA4, associated with all drought indices for PH, had the highest contribution for RILs 23, 95, and 125. FA5, linked to all drought indices for SPAD, had the highest contribution for RILs 41, 64, 101, 124, and 132, which exhibited higher STI (0.63–1.25%) and RSI (0.75–1.24%). FA6, composed of YSI, RSI, SSI, and TOL for RWC, showed the highest contribution for RILs 23, 48, and 104. Furthermore, FA7, which is composed of STI for biological and grain yields, as well as RWC, exhibited the greatest impact on RILs 41 and 159, while having the least impact on RIL 90, as indicated by the negative estimates of TOL and SSI for BY (-27%, -0.12%) and GY (-2%, -0.01%) (Supp. Tables 11 and 12). Additionally, FA8, which is associated with STI for SPAD (0.69%), TGW (0.81), and HI (0.94%), had the most significant effect on RIL 6 (Supp. Table 5).

In the second year of our study, we conducted an analysis of drought indices and classified them into eight factors (FA1-FA8), as outlined in Supp. Tables 13–19. FA1, which includes SSI, YSI, RSI, and TOL for biological and grain yields, had a notable impact on RILs 48 and 55 (Fig. 6d). FA2, which focuses on drought indices for harvest index, exerted the most influence on RILs 16, 56, 99, 103, 140, 150, and 162, while showing minimal impact on RIL 149, as indicated by negative estimates of TOL (-2.09%) and SSI (-0.14%) for HI (Supp. Table 17). FA3, encompassing all drought indices for SPAD, had the greatest impact on RILs 46, 99, 103, and 149 (Supp.Table 13). FA4, related to drought indices for RWC, significantly affected RILs 46 and 54, while FA5, focusing on plant height, had the most impact on RILs 99 and 103.Furthermore, FA6, consisting of drought indices for TGW, had a significant impact on RILs 22 and 69, with high RSI values observed in these RILs. FA7 had the most significant impact on RIL 95, with high estimates of STI for BY (1.29%), GY (1.51%), and HI (1.18%) (Supp. Tables 17–19). Moreover, FA8, associated with STI for TGW, PH, and RWC, had the most significant effect on RILs 50 and 101 (Fig. 5d).

### Drought resistance index (DRI)

The DRI values were utilized to differentiate between drought-sensitive and drought-tolerant RILs that demonstrated exceptional performance under drought stress conditions. The DRI values for morpho-physiological and yield-related traits can be referenced in Tables 7 and 8. In the year 2020, the DRI for SPAD ranged from 0.13% (RIL 3) to 2.16% (RIL 40), while the DRI for RWC ranged from -0.24% (RIL 133) to 2.29% (RIL 12). The highest relative DRI for PH was observed in RIL 43 (32.76%), with the lowest index recorded for RIL 61 (-0.04) (Table 7). During the initial year, RIL 146 displayed the highest DRI for TGW at 2.15%, whereas RIL 87 had the lowest DRI at -0.02%. The DRI for HI varied from -4.69 (RIL 65) to 5.77 (RIL 59). RIL 4 exhibited high DRI values for BY, while RIL 59 had the lowest DRI at -0.54%. Regarding GY, the highest DRI was observed in RIL 12 (3.12%), while the lowest was found in RIL 90 (-0.03%) (Table 7).

**Table 7:** Drought resistance index (DRI) of 167 wheat recombinant inbred lines (RILs) along with their two parents for morpho-physiological traits in 2020–2021.

Code	SPAD	RWC	PH	TGW	HI	BY	GY	Code	SPAD	RWC	PH	TGW	HI	BY	GY
G1	1.48	1.25	0.53	1.04	2.3	1.88	1.89	G58	1.22	0.16	1.91	1.15	− 3.61	1.91	0.73
G2	1.81	0.94	1.11	0.74	1	1.45	1.22	G59	1.49	1.76	1.87	0.83	5.77	-0.54	0.62
G3	0.13	1.29	0.23	1	2.93	0.95	1.25	G60	1.08	1.37	0.63	0.42	− 0.81	1.9	1.31
G4	0.85	1.57	1.63	0.71	− 0.86	2.8	2.08	G61	1.66	0.43	− 0.04	0.5	− 0.53	1.08	0.83
G5	1.48	0.76	1.44	1.37	2.49	1.11	1.5	G62	0.44	1.2	1.39	0.72	1.63	1.5	1.57
G6	1.73	0.59	1.77	0.7	− 0.57	1.28	0.9	G63	0.61	0.31	1.22	0.79	1.68	-0.04	0.26
G7	0.52	0.98	1.61	0.6	3.8	1.38	2.11	G64	1.35	1.49	1.39	0.59	0.4	0.68	0.56
G8	1.16	0.92	0.39	1.15	0.29	2.05	1.63	G65	1.15	0.78	0.06	0.94	− 4.69	1.44	0.59
G9	0.58	1.02	0.78	0.83	1.22	0.66	0.74	G66	0.87	1.08	0.34	0.64	1.51	0.97	1.25
G10	1.01	0.47	0.1	1.6	− 1.13	1.37	0.99	G67	0.66	1.06	0.56	0.71	3.13	0.81	1.37
G11	0.84	1.76	0.53	1.29	1.63	2.22	2.12	G68	1.1	0.82	1.09	0.98	− 0.37	0.99	0.86
G12	1.29	2.29	0.73	1.58	2.73	2.56	3.12	G69	0.48	1.2	1.36	0.58	1.31	0.67	0.82
G13	1.33	1	0.96	1.4	− 2.77	1.65	0.86	G70	1.18	0.75	1.21	1.14	0.81	1.46	1.39
G14	0.93	1.31	2.12	0.94	− 2.06	1.65	0.99	G71	1.5	1.08	0.7	1.47	0.12	1.25	0.96
G15	0.71	1.53	1.17	0.41	2.07	1.43	1.62	G72	0.87	0.8	0.25	1.2	3.41	0.11	0.79
G16	0.83	0.63	1.23	0.82	1.1	1.27	1.1	G73	0.48	0.98	0.65	1.14	− 0.35	2.32	1.54
G17	1.09	0.9	0.23	1.96	1.44	0.41	0.61	G74	1	0.96	0.54	0.6	3.81	0.35	0.82
G18	1.48	1.69	0.23	1.86	1.91	1.34	1.49	G75	0.74	0.49	0.24	0.58	− 0.35	0.68	0.51
G19	1.02	1.86	0.81	1.28	− 0.6	2.48	1.7	G76	1.44	0.82	0.23	0.61	0.21	0.14	0.14
G20	0.86	1.35	0.54	0.76	1.93	1.07	1.12	G77	1.21	0.8	1.53	0.81	− 0.37	0.69	0.51
G21	1.66	1.02	2.09	1.35	− 0.36	1.7	1.49	G78	1.16	1.53	0.72	0.48	− 0.67	1.14	0.86
G22	0.8	1.06	2.49	0.24	0.06	2.31	1.82	G79	1.19	0.67	0.5	0.9	0.91	0.99	0.82
G23	0.75	1.65	1.54	1.37	3.56	1.02	1.62	G80	0.39	1.35	0.94	1.01	− 0.39	1.5	0.94
G24	0.92	2	0.34	0.95	− 0.02	1.73	1.42	G81	0.28	1.04	0.87	0.61	0.9	0.51	0.62
G25	1.23	1.27	0.27	0.91	0.87	1.69	1.88	G82	1.5	0.82	0.42	1.09	1.11	0.37	0.47
G26	1.02	1.31	0.94	1.83	0.35	1.35	0.98	G83	1.12	0.47	0.51	1.03	0.72	1.06	0.86
G27	0.44	0.71	0.87	1.19	1.6	0.98	1.26	G84	0.26	0.65	0.77	1.6	2.43	0.26	0.7
G28	0.88	1.94	0.78	1.35	− 0.38	1.82	1.29	G85	0.91	1.02	0.24	1.2	0.65	1.68	1.29
G29	1.17	0.49	1.6	0.4	1.28	0.59	0.7	G86	1.15	0.31	0.37	1.53	0.87	0.4	0.47
G30	1.13	0.92	1.06	0.98	0.09	1.56	1.54	G87	0.75	1.65	1.31	− 0.02	2.37	0.89	1.21
G31	0.17	0.82	0.89	0.73	− 0.14	1.22	1.01	G88	0.72	1.1	0.58	1.08	1.65	1.08	1.29
G32	1.44	0.84	1.19	1.1	0.73	0.67	0.72	G89	0.96	0.8	0.06	0.5	1.36	1.02	0.92
G33	1.14	0.9	1.15	0.62	1.04	0.55	0.77	G90	0.69	1.65	1.53	0.64	0.21	-0.12	-0.03
G34	0.92	0.9	0.31	1.17	2.05	0.57	0.87	G91	0.54	0.78	0.44	1.13	3.22	0.29	0.82
G35	1.38	1.1	0.46	0.79	0.1	1.32	1.1	G92	1.45	0.33	0.87	0.88	2.19	0.3	0.59
G36	1.2	0.39	1.07	1.26	2.07	0.48	0.72	G93	1.13	0.37	0.32	1.75	2.71	0.35	0.77
G37	1.12	0.98	1.02	1.28	1.2	1.07	1.1	G94	0.56	1.37	1.17	1.32	4.26	0.44	1.23
G38	1.05	1.16	0.75	0.24	2.49	0.67	0.93	G95	0.71	0.88	0.74	1.42	1.03	0.45	0.66
G39	1.57	0.12	1.13	1.39	0.59	0.89	0.82	G96	0.94	1.41	1.09	0.84	2.78	1.24	1.5
G40	2.16	1.02	0.78	0.36	1.09	1.1	1.1	G97	1.61	1.04	0.71	0.79	3.72	1.29	2.02
G41	1.04	0.45	1.42	0.59	− 1.01	1.66	0.92	G98	0.95	0.78	0.04	1.36	− 1.38	0.71	0.24
G42	1.32	0.92	1.4	1.49	− 1.68	1.56	0.75	G99	0.33	1.41	1.1	1.07	1.73	0.56	0.81
G43	0.56	0.9	32.76	1.81	1.05	1.35	1.24	G100	1.25	0.31	0.5	1.31	1.61	0.42	0.65
G44	0.98	0.59	0.67	0.94	1.8	0.48	0.57	G101	1.07	0.78	0.35	1.55	− 1.17	1.57	0.9
G45	0.91	1.88	0.94	0.64	0.94	1.76	1.71	G102	1.77	1.65	0.63	1.23	0.09	0.52	0.42
G46	0.94	1.02	0.75	0.52	0.69	0.48	0.55	G103	1.42	1.27	0.98	1.12	− 1.11	1.54	0.81
G47	1.61	− 0.02	0.62	0.71	1.48	0.07	0.19	G104	1.54	0.92	1.03	0.31	1.95	-0.19	0.13
G48	0.64	1.57	1.46	0.69	− 0.52	1.3	0.91	G105	0.64	0.55	0.03	0.37	2.12	1.01	1.18
G49	1.27	1.14	1.49	0.94	− 1.31	1.54	0.96	G106	0.49	0.69	0.7	0.42	1.36	0.46	0.51
G50	1.02	1.59	1.41	1.05	2.69	1.64	1.91	G107	1.2	1.37	0.45	0.72	1.44	1.44	1.3
G51	0.72	0.2	0.42	1.08	− 2.11	0.89	0.34	G108	1.26	1.37	1.1	1.33	0.52	1.37	1.21
G52	1.36	1.04	0.82	1.12	1.65	1.14	1.19	G109	0.32	1.06	0.98	0.98	1.37	0.85	0.91
G53	0.71	1.12	0.6	0.32	1.94	0.51	0.66	G110	0.97	1.45	0.77	1.01	2.53	1.15	1.4
G54	0.83	1.04	1.35	0.5	2.25	0.85	1.27	G111	1.1	1.35	0.63	1.04	1.45	1.12	1.19
G55	1.44	1.82	0.79	0.75	2.7	0.77	1.29	G112	0.88	1.61	0.91	0.84	1.27	1.07	1.1
G56	1.47	0.55	0.85	1.26	1.58	1.43	1.64	G113	0.63	0.31	0.54	0.84	1.4	0.27	0.48
G57	1.42	0.88	0.94	1.19	− 0.39	0.76	0.67	G114	0.93	0.51	0.22	0.84	− 0.43	0.82	0.72

Code	SPAD	RWC	PH	TGW	HI	BY	GY
G115	0.94	1.25	0.96	1.03	0.49	1.12	1.16
G116	0.7	1.37	0.69	0.55	1.52	1.29	1.15
G117	0.68	0.35	0.64	1.43	2.37	0.74	1.01
G118	1.18	0.49	0.77	0.65	0.19	0.74	0.66
G119	0.86	1.39	0.82	0.88	2.85	0.7	1.08
G120	0.86	0.67	0.17	1.08	2.94	0.59	0.99
G121	0.9	0.57	0.82	0.92	1.62	0.62	0.75
G122	0.55	1.61	1.15	0.45	1.54	0.29	0.52
G123	1.1	0.63	0.17	1.01	1.47	0.48	0.62
G124	1.04	0.8	0.99	1.26	1.23	0.65	0.83
G125	0.7	0.67	0.73	1.39	1.55	0.93	1.19
G126	0.88	0.61	1.1	0.79	1.85	0.71	0.95
G127	1.48	1.02	0.54	0.83	1.97	0.78	1.13
G128	0.59	1.18	0.34	1.4	1.95	1	1.2
G129	1.19	1.16	0.51	1.16	3.14	0.74	1.38
G130	0.9	0.73	0.46	0.8	2.55	0.64	0.99
G131	1.05	0.65	0.75	1.14	1.4	0.57	0.81
G132	0.95	2.04	0.99	1.19	0.77	1.15	1.08
G133	1.32	− 0.24	0.04	1.3	− 0.35	0.19	0.13
G134	1.27	0.08	0.25	1.59	0.91	1.12	1.22
G135	1.5	0.59	0.97	0.78	2.37	0.41	0.66
G136	0.71	1.51	0.3	0.7	1.11	0.88	0.97
G137	0.98	0.2	1.02	0.82	2.86	− 0.04	0.42
G138	1.08	0.43	0.8	0.5	− 0.16	0.97	0.82
G139	0.98	0.86	1.06	1.28	− 1.07	1.47	0.74
G140	0.89	0.8	1.23	1.31	0.21	1.18	1.08
G141	0.91	1.47	0.85	1.22	1.84	1.14	1.31
G142	0.7	0.98	0.56	1.68	0.16	0.94	0.82
G143	1.37	0.76	0.2	1.17	0.36	0.58	0.49
G144	1.5	0.8	1.37	0.06	0.41	0.82	0.76
G145	1.3	1.53	0.68	1.13	− 1.02	1.4	0.98
G146	1.69	1.43	0.46	2.15	2.35	1.02	1.48
G147	1.12	0.53	0.31	1.54	1.39	0.65	0.9
G148	0.57	0.86	0.42	1.1	1.27	0.79	0.73
G149	0.34	1.55	1.02	0.86	1.57	0.83	0.96
G150	0.82	1.16	0.54	0.83	0.47	1.36	1.16
G151	1.06	1.16	0.46	0.91	− 2.66	2.1	0.83
G152	0.72	1.02	0.34	1.59	0.4	0.94	0.9
G153	0.73	1.02	1.22	1.49	1.37	1.1	1.16
G154	1.37	0.71	0.49	0.65	0.48	0.55	0.58
G155	0.74	1.04	0.63	1.78	0.22	1.04	0.93
G156	1.13	1.04	0.77	1.32	0.61	1.13	1.09
G157	0.43	0.8	0.72	1.23	1.59	0.66	0.78
G158	0.42	1.55	1.34	0.46	0.26	1.14	1.03
G159	0.78	0.94	0.69	1.12	1.1	0.94	1.12
G160	0.97	1.43	0.68	0.42	0.96	1.19	1.11
G161	1.44	0.96	0.53	1.09	0.08	1.63	1.04
G162	0.82	0.96	0.53	0.53	0.89	0.46	0.48
G163	0.72	1.29	0.46	2.11	0.23	0.93	0.66
G164	0.38	1.43	0.63	1.17	1.74	0.63	0.79
G165	0.82	0.22	1.32	1.21	1.52	0.61	0.85
G166	0.79	0.82	0.61	0.81	1.46	0.86	1
G167	0.56	0.22	0.45	0.76	1.46	0.7	0.77
G168	1.29	1.71	1.06	0.92	1.24	1.13	1.2
G169	1.28	0.78	1.23	1.56	1.45	1.08	1.26

SPAD, RWC, PH, TGW, HI, BY, and GY were the abbreviations estimation of chlorophyll using SPAD, relative water content, plant height, thousand grain weight, harvest index, biological yield, and grain yield.

**Table 8:** Drought resistance index (DRI) of 167 wheat recombinant inbred lines (RILs) along with their two parents for morpho-physiological traits in 2021–2022.

Code	SPAD	RWC	PH	TGW	HI	BY	GY	Code	SPAD	RWC	PH	TGW	HI	BY	GY
G1	0.59	1.79	0.66	2.32	1.23	− 2.22	0.11	G58	1.01	0.11	3.93	1.54	1.96	3.02	2.36
G2	1.12	0.95	0.77	0.87	− 1.43	2.08	− 0.11	G59	0.50	1.26	1.11	0.49	0.46	1.98	1.01
G3	1.83	1.74	1.51	1.77	2.34	− 3.09	0.70	G60	0.98	0.53	0.48	1.08	1.85	− 0.07	1.34
G4	1.67	2.00	2.12	0.94	0.47	0.95	0.62	G61	0.58	1.37	0.26	− 0.26	0.22	3.25	1.23
G5	2.06	0.95	3.02	0.72	0.61	1.11	0.88	G62	− 0.47	1.26	0.52	0.35	− 0.40	0.64	-0.11
G6	1.63	0.74	1.09	0.69	1.47	− 1.89	0.35	G63	1.41	0.05	1.60	1.99	0.08	2.52	1.06
G7	0.40	1.84	1.33	0.69	1.69	− 0.94	0.77	G64	1.49	2.26	1.44	2.00	0.79	3.51	1.54
G8	0.54	0.89	0.77	1.12	0.70	− 0.72	0.11	G65	0.78	0.84	− 0.31	0.98	0.91	− 0.74	0.48
G9	0.63	0.47	0.85	0.36	1.39	− 2.40	0.15	G66	0.67	0.58	0.13	1.23	1.39	− 0.35	0.91
G10	1.45	0.11	0.52	1.84	2.66	1.68	2.32	G67	1.82	0.42	− 0.68	0.81	2.09	2.40	2.25
G11	0.00	1.11	0.48	1.25	0.60	− 1.32	− 0.05	G68	0.63	0.42	1.18	1.34	1.19	0.65	1.15
G12	0.63	2.84	0.79	1.39	− 0.25	1.76	0.35	G69	0.74	1.05	− 0.15	0.27	2.10	− 2.26	0.68
G13	1.54	1.63	0.42	1.27	− 1.25	1.13	− 0.18	G70	1.61	2.32	1.42	1.20	− 0.03	4.34	1.32
G14	1.10	1.47	2.89	1.74	0.76	0.94	0.70	G71	1.04	0.53	0.28	1.41	1.09	2.79	1.63
G15	1.80	1.68	1.81	0.01	− 0.20	1.68	0.53	G72	0.47	0.16	− 0.07	1.02	1.30	− 1.50	0.33
G16	1.27	1.95	1.03	1.17	0.94	− 0.42	0.38	G73	0.24	1.84	0.59	1.75	0.54	1.11	0.71
G17	− 0.66	0.89	0.52	1.68	1.22	1.45	1.24	G74	− 0.31	0.68	1.20	0.88	− 0.59	2.10	0.44
G18	1.16	1.89	0.94	1.66	1.26	− 1.39	0.37	G75	1.59	0.42	0.52	0.86	1.64	− 2.45	0.33
G19	1.08	1.63	1.44	1.40	1.83	− 3.86	0.37	G76	1.24	1.68	− 0.31	1.49	2.75	− 3.02	0.80
G20	− 0.15	2.05	0.09	0.92	− 1.50	0.99	− 0.38	G77	− 0.29	0.89	0.70	0.41	1.49	1.15	1.44
G21	0.49	0.32	2.10	1.17	− 0.17	2.91	0.97	G78	1.98	1.74	0.20	1.16	0.61	1.32	0.88
G22	0.79	0.68	1.75	0.81	0.30	2.95	1.41	G79	1.88	0.63	0.15	1.21	1.57	0.07	1.10
G23	0.91	0.84	1.81	1.79	0.58	1.78	0.88	G80	1.26	2.11	0.63	0.80	− 0.38	3.18	1.01
G24	2.36	2.53	1.38	1.68	1.80	− 1.18	0.80	G81	0.91	0.58	0.52	0.63	1.24	− 1.08	0.44
G25	0.24	2.42	− 0.07	0.73	2.40	0.19	1.74	G82	2.16	1.37	1.20	1.25	1.16	2.47	1.83
G26	1.00	2.00	1.60	2.10	− 1.00	5.42	1.08	G83	1.45	0.68	0.57	1.04	0.92	− 0.76	0.27
G27	2.56	0.68	0.92	1.89	0.21	1.32	0.66	G84	1.54	1.00	− 0.02	1.12	0.67	− 1.25	0.07
G28	1.41	1.21	2.19	0.97	− 0.13	− 0.25	− 0.16	G85	1.42	0.58	0.35	1.10	0.63	3.23	1.50
G29	1.14	0.53	2.69	0.95	− 0.05	3.21	0.80	G86	1.02	0.30	0.54	1.15	0.89	1.57	1.10
G30	1.26	0.68	2.03	1.48	− 0.01	1.91	0.60	G87	0.79	1.51	1.70	0.93	0.66	3.68	1.76
G31	1.78	0.79	1.75	1.22	0.18	1.31	0.49	G88	1.77	1.00	0.44	1.12	1.24	− 0.68	0.63
G32	0.75	0.37	1.27	1.66	0.43	0.21	0.29	G89	0.65	1.19	0.45	0.64	1.38	0.78	1.14
G33	0.58	0.53	0.72	1.17	0.83	− 1.18	0.16	G90	1.26	1.09	1.03	0.86	1.24	2.38	1.60
G34	1.71	1.05	1.14	1.12	0.74	0.71	0.70	G91	0.70	0.70	1.04	1.14	1.58	0.93	1.15
G35	− 0.13	0.74	0.72	0.28	− 1.13	2.24	− 0.09	G92	− 0.04	0.32	0.68	0.72	2.14	1.95	2.11
G36	1.45	0.84	2.16	1.02	0.16	2.54	0.99	G93	0.67	0.35	0.50	1.37	1.07	0.16	0.87
G37	1.59	0.84	1.36	1.96	0.65	0.30	0.55	G94	1.47	1.18	0.87	1.38	1.10	− 0.19	0.62
G38	0.78	− 0.16	0.96	− 0.01	0.61	− 0.34	0.22	G95	0.28	1.05	0.09	1.52	1.32	1.82	1.70
G39	0.11	− 0.11	1.79	1.31	1.21	− 0.81	0.59	G96	0.85	2.63	0.46	0.86	1.95	− 1.01	0.86
G40	2.19	2.00	0.74	0.94	0.55	− 2.03	− 0.16	G97	0.13	1.21	− 0.02	1.06	1.97	− 2.19	0.77
G41	0.20	0.47	1.86	1.00	− 0.28	0.83	0.07	G98	1.10	1.84	0.33	1.23	1.37	2.77	1.83
G42	− 0.76	0.89	2.78	1.20	0.39	1.52	0.75	G99	1.16	1.68	1.60	0.86	1.63	2.86	1.96
G43	1.73	0.63	1.99	1.63	− 0.54	2.44	0.38	G100	0.14	− 0.47	0.52	1.49	2.65	− 2.45	0.82
G44	0.78	0.58	1.20	1.13	2.40	− 1.98	0.95	G101	− 0.09	1.32	1.07	1.39	0.61	2.63	1.37
G45	1.74	1.26	0.85	1.20	0.29	− 0.05	0.18	G102	− 0.04	1.00	0.59	1.01	0.48	− 0.21	0.27
G46	1.33	2.00	0.72	1.03	0.20	0.16	0.18	G103	1.49	1.26	0.59	0.53	2.12	− 0.72	1.01
G47	2.12	− 0.11	1.44	1.12	− 1.10	3.32	0.73	G104	0.11	0.53	1.09	0.70	0.06	1.21	0.41
G48	2.57	1.79	0.92	0.70	0.35	0.23	0.27	G105	1.81	0.63	0.61	1.05	0.91	0.09	0.70
G49	2.39	0.63	1.20	1.81	− 0.03	1.73	0.53	G106	0.63	1.42	0.07	1.07	2.11	− 3.92	0.27
G50	0.89	1.74	1.66	1.22	1.55	1.75	1.61	G107	− 0.32	1.58	0.66	0.83	1.76	1.02	1.72
G51	0.81	0.00	1.18	1.42	− 0.74	4.71	1.08	G108	0.89	1.21	0.11	1.25	1.66	− 1.25	0.67
G52	1.90	0.53	0.35	0.91	1.14	− 3.28	0.04	G109	1.17	0.95	0.13	1.29	1.06	2.44	1.54
G53	2.15	1.74	0.66	1.15	− 0.29	1.27	0.29	G110	1.20	2.32	0.74	1.17	0.49	2.54	1.12
G54	0.58	2.11	0.83	2.10	2.50	− 2.95	0.64	G111	1.85	1.42	0.70	1.24	1.20	1.57	1.37
G55	0.69	2.26	0.44	0.57	0.22	0.72	0.37	G112	0.75	1.21	0.96	1.44	− 0.12	3.25	1.01
G56	1.45	0.89	1.31	1.40	0.41	0.44	0.38	G113	1.10	0.53	0.28	0.98	2.43	0.44	1.54
G57	1.75	0.74	1.07	1.32	− 0.23	2.93	0.77	G114	− 0.26	0.63	− 0.09	0.36	0.70	1.22	0.86

Code	SPAD	RWC	PH	TGW	HI	BY	GY
G115	1.08	2.16	1.03	0.76	2.22	0.53	1.55
G116	1.06	1.63	0.74	0.81	1.94	0.90	1.57
G117	0.79	0.53	− 0.02	1.52	2.24	0.46	1.81
G118	1.37	0.79	0.63	0.38	1.70	2.82	2.07
G119	1.10	0.84	0.98	1.04	2.30	− 3.39	0.64
G120	1.50	− 0.32	− 0.28	1.72	1.93	− 0.21	1.04
G121	1.68	− 0.05	0.81	0.89	0.69	3.78	1.63
G122	1.53	1.26	0.70	0.34	1.61	− 0.26	1.19
G123	0.35	0.11	0.92	1.03	− 0.71	2.47	0.40
G124	− 0.63	0.00	0.63	− 0.02	0.71	− 2.01	− 0.07
G125	2.26	1.05	1.27	1.81	1.68	2.24	2.07
G126	1.30	1.32	1.27	0.93	0.97	− 0.56	0.42
G127	1.68	0.79	0.39	0.61	1.89	1.46	1.68
G128	0.44	1.63	0.17	1.41	2.26	− 4.46	0.27
G129	0.60	1.26	0.50	1.76	1.45	2.63	1.99
G130	1.04	1.53	0.04	0.91	0.01	2.03	0.70
G131	0.77	1.16	− 0.24	0.37	− 1.25	2.59	0.20
G132	1.30	1.16	0.79	1.43	1.40	0.92	1.10
G133	1.06	0.05	0.74	1.25	1.58	0.71	1.24
G134	0.77	0.37	0.63	1.09	0.08	1.69	0.51
G135	0.48	1.11	1.84	1.07	0.75	0.53	0.60
G136	1.64	1.11	0.20	1.22	0.11	0.99	0.44
G137	1.41	− 0.53	0.42	1.03	1.78	2.79	2.12
G138	1.17	1.53	0.48	0.25	1.54	0.62	1.15
G139	1.53	1.53	0.72	1.03	0.69	4.29	1.98
G140	− 0.46	1.26	1.03	0.91	1.58	2.05	1.74
G141	0.63	0.47	0.39	1.57	1.86	− 1.16	1.02
G142	0.54	1.05	0.83	1.31	1.22	3.09	1.94
G143	1.29	0.16	0.96	0.80	0.87	1.98	1.21
G144	1.57	0.11	1.38	0.28	− 0.91	3.16	0.64
G145	1.14	1.42	1.92	1.66	− 0.82	0.72	− 0.26
G146	1.45	1.11	0.96	2.13	0.93	0.30	0.73
G147	0.18	0.68	− 0.39	1.51	2.57	1.09	2.16
G148	2.31	0.58	0.81	1.34	1.94	0.49	1.32
G149	1.13	2.26	1.75	1.36	0.62	4.38	2.16
G150	1.07	2.26	1.11	1.59	2.18	0.88	1.94
G151	2.21	1.00	0.33	0.83	− 0.05	0.90	0.29
G152	1.23	0.26	0.46	1.41	1.25	1.08	1.32
G153	1.79	0.53	1.95	1.79	3.23	1.52	2.89
G154	0.73	0.74	− 0.15	0.80	0.20	4.09	1.41
G155	1.24	0.16	1.16	0.97	1.69	− 3.46	0.17
G156	2.13	1.47	1.95	0.31	2.23	3.79	2.80
G157	0.44	0.16	1.90	1.32	0.35	3.11	1.21
G158	0.47	1.00	2.10	0.14	− 0.12	3.69	1.41
G159	1.30	1.05	1.05	0.96	0.82	− 0.35	0.40
G160	0.76	1.89	− 0.92	1.12	3.26	− 2.21	1.68
G161	− 0.27	1.16	1.40	1.57	1.37	− 0.26	0.93
G162	2.56	1.26	1.09	0.81	1.77	− 1.45	0.57
G163	0.88	0.53	0.42	1.59	2.36	− 0.18	1.55
G164	1.59	2.05	0.28	1.29	0.45	0.51	0.48
G165	1.74	0.21	− 0.04	1.06	1.25	2.44	1.74
G166	1.61	0.68	0.81	0.94	3.69	− 0.23	3.09
G167	0.72	− 0.11	1.09	0.87	1.71	− 0.37	1.32
G168	2.33	1.95	1.46	0.89	2.34	− 1.89	1.06
G169	− 0.87	0.95	1.51	0.97	0.14	3.35	1.26

SPAD, RWC, PH, TGW, HI, BY, and GY were the abbreviations estimation of chlorophyll using SPAD, relative water content, plant height, thousand grain weight, harvest index, biological yield, and grain yield.

In the second year of evaluation, a total of 167 RILs along with their two parents were assessed for SPAD levels. Among these, 153 RILs demonstrated positive DRI values, indicating a high level of tolerance to drought, while the remaining sixteen RILs showed negative DRI values, suggesting susceptibility to drought. Further analysis of SPAD, RWC, and PH revealed that RILs 48, 12, and 58 exhibited superior tolerance to drought compared to other RILs, with DRI values of 2.57%, 2.84%, and 3.93%, respectively (Table 8). In terms of grain yield components such as TGW and BY, RILs 1 and 26 displayed heightened responsiveness to drought stress, with DRI values of 2.32% and 5.52%, respectively. Notably, RIL 166 emerged as the top performer under drought stress conditions, boasting DRI values exceeding 3 for HI and GY. Additionally, RILs 26, 51, 70, 139, 149, and 154 stood out as the top-performing RILs under drought stress, with DRI values for BY ranging from 4.09 to 5.42% (Table 8). These results underscore the potential of these select RILs to excel in environments prone to drought, highlighting their resilience and adaptability in challenging conditions.

### Drought-tolerant RILs selected based on an integrated MGIDI and stress related indices

Based on the integrated analysis of drought tolerance indices (STI, SSI, TOL, YSI, RSI), cluster analysis, and the MGIDI index, a consistent drought-tolerant RILs was identified, together with genotypes highly susceptible to stress. The most strongly tolerant RILs including RILs 101, 41, 33, 159, and 95 demonstrated high STI and YSI values, low SSI scores, maintained superior grain yield under drought stress, and were consistently ranked in the top clusters (e.g., Cluster I) and selected through the 10% MGIDI selection pressure. These genotypes exhibit the favorable combination of high yield potential and stability, with physiological mechanisms such as higher RWC and BY contributing to their resilience. Conversely, RILs 12, 156, and those within Cluster III were classified as susceptible; while some (e.g., RIL 12) achieved high yield under well-watered conditions, they showed the highest SSI values and significant yield losses under drought, indicating poor stress adaptability and high sensitivity.

## Discussion

### Response of morpho-physiological traits to drought stress

Plants demonstrate a sophisticated response to stress by utilizing acclimation and adaptation strategies at the cellular, molecular, and morphological levels^64^. This response involves the development of tolerance mechanisms to combat stressors^65^. The ability of plants to endure challenging conditions is vital for their survival^66,67^. Studies have shown that the productivity and morpho-physiological traits of wheat are significantly impacted by the specific cultivar used and the growing environment, as well as their interactions. Therefore, conducting multi-environment trials (METs) is crucial for accurately selecting RILs with desired traits^68–72^. In the current study, we assessed 167 RILs along with their two parents in field experiments under well-watered and drought-stressed conditions over a two-year period to identify and select drought-tolerant RILs based on the evaluation of morpho-physiological traits, drought indices, *D* value, and MGIDI. Our results confirmed a significant G × E interaction for grain yield and most traits. This interaction, characterized by crossover effects where top performers changed ranks across environments, underscores the necessity of stability analysis within METs to identify genotypes with reliable performance, as defined by the differential response of genotypes to environmental variation^69^. Understanding the intricate nature of drought stress and identifying the key parameters responsible for drought tolerance are essential steps in the selection of drought-tolerant RILs. When exposed to water deficit stress, all measured parameters in our germplasm displayed varying degrees of decline. Notably, leaf chlorophyll concentrations (SPAD) and RWC exhibited the most significant decreases under water deficit conditions, with reductions of up to 26%. Monitoring chlorophyll content, in conjunction with other traits, presents a promising yet underexplored approach to breeding for drought tolerance. The drought tolerance of certain wheat cultivars relies on the mobilization of carbon from stems to support grain filling, prioritizing grain fill over the length of the plant cycle^73^. In such instances, the stay-green phenotype should be avoided as it hinders the utilization of vegetative biomass for grain filling, and chlorophyll degradation may serve as a tolerance response, depending on the RILs. These findings underscore the importance of screening for chlorophyll content under specific conditions, such as drought, as it could significantly enhance breeding programs. The findings align with those of Kettani et al.^74^, who observed a decrease in physiological parameters, such as SPAD and RWC, in 16 durum wheat varieties over a two-year period. This decline was linked to water deficit during crucial growth stages like booting, heading, anthesis, and physiological maturity. Similarly, Xu et al.^75^ and Javed et al.^76^ reported comparable reductions in spring wheat and elite bread wheat, respectively, under drought conditions. Susceptible wheat varieties exhibited a notable decrease in SPAD values, while tolerant varieties showed higher SPAD readings^74^. Our study unveiled a strong positive correlation between RWC and both BY and GY in both experimental settings. Recent work similarly identifies physiological traits like RWC, and photosynthetic parameters as pivotal for distinguishing drought-tolerant wheat genotypes in integrated, multi-trait analyses^46^. This discovery supports the theoretical framework proposed by Wasaya et al.^77^ regarding the interconnectedness of these three traits. RWC emerges as a crucial indicator of drought stress tolerance in wheat and can aid in selecting plants capable of enduring drought conditions^26,78^. Studies have shown that plant species can experience a reduction of over 50% in relative water content under stressful conditions, highlighting the significant impact of drought on plant water status^79,80^. Understanding and mitigating the effects of water stress on crop productivity is crucial. In our study, we observed a decrease in yield parameters such as BY, HI, and GY due to drought stress, which can be attributed to the shortened grain filling period and accelerated leaf senescence. Bhandari et al. ^42^ found that wheat yield was reduced by 24% under heat stress and 48% under combined heat-drought stress, revealing the severe cumulative impact of such environments. Furthermore, their stability analysis confirmed that environment had the greatest effect on trait expression, supporting our finding that G × E is a major factor determining performance in stress breeding program. In our study, the different RILs tested displayed distinct responses to drought conditions, indicating significant variations in the level of damage caused by this stressThe wide range in yield loss and TGW reduction under drought highlights the considerable genetic variability for stress sensitivity within the RIL population. This diversity of response, which aligns with previously reported losses for wheat yield components^34^, provides a critical foundation for selecting genotypes with genetic resilience. In barley, drought tolerance indices such as MP, GMP, and STI were used to identify genotypes with high and stable yields under stress, illustrating how integrated index-based selection can complement yield stability analysis^81^. Overall, these findings underscore the importance of addressing water stress in agriculture to ensure optimal crop productivity and sustainability. Our research has revealed a noteworthy connection between grain yield and HI and BY, suggesting that the decline in yield under drought conditions can be linked to reductions in both HI and BY. The decrease in biological yield is believed to be caused by the premature ripening of photosynthetic parts, which impeded photosynthesis and ultimately led to a lower grain yield. These results align with the findings of Johari-Pireivatlou et al.^82^, who studied the effects of drought stress on the biological yield of four wheat genotypes and observed that resistant genotypes displayed higher biological and grain yields.

Crop drought resistance is a multifaceted trait influenced by numerous genes, as widely recognized in the scientific community. Depending on a single indicator to gauge drought tolerance is deemed inadequate^26^. Instead of fixating on a singular trait, it is imperative to take into account the response of multiple traits to drought conditions. A comprehensive evaluation of drought resistance, achieved by amalgamating various traits and employing multivariate techniques, is deemed more precise and dependable than relying on a single indicator^83^. PCA emerges as a versatile tool for multivariate analysis, offering valuable insights into the intricate structure of complex datasets. PCA not only diminishes the dimensionality of datasets while preserving most of the variability but also identifies the pivotal components steering the variation. The outcomes of PCA underscored the significance of GY, BY, and RWC in one dimension of the component analysis, showcasing strong correlations among them.

Principal component and drought tolerance (*D* value) analyses effectively divided the population, showing RILs with different adaptation strategies. This separation revealed a range from high tolerance to high sensitivity. RILs such as 76, 104, and 144 were classified as highly drought-tolerant, exhibiting consistently superior integrated performance (high *D* values > 0.599). An intermediate group, including RILs 33, 40, 59, 126, and 140, demonstrated moderate tolerance (*D* values 0.206–0.503), suggesting partial but incomplete resilience mechanisms. In contrast, a group of sensitive RILs, represented by RILs 18, 80, 153, and 157, achieved high yields only under well-watered conditions, with poor stress adaptation (low *D* values). This clear phenotypic separation is highly valuable. The highly tolerant and highly sensitive RILs provide an ideal contrasting pair for comparative genomics and physiology studies aimed at understanding drought resilience mechanisms. Furthermore, the tolerant lines represent direct candidates for targeted crossing in breeding programs to enhance yield stability in water-limited environments.

### Genetic variability for various traits

Genetic improvement is a continuous process that involves the creation and utilization of genetic variability. Understanding the extent of variation and diversity within existing genetic materials, especially in challenging conditions, can aid plant breeders in developing effective breeding strategies and selection criteria to enhance desired traits in stressful environments. Exploring new genetic resources in wheat, analyzing variability, and employing appropriate breeding techniques are crucial steps in producing innovative cultivars with increased yields and improved nutritional content in drought-prone regions like Iran. High PCV and GCV indicate a significant level of genetic diversity within a population. Conversely, traits with low PCV and GCV values suggest a reduced level of diversity^84,85^. In this study, PCV was higher than GCV, indicating minimal environmental influence on the phenotypic expression of traits. Overall, genetic improvement plays a vital role in enhancing crop productivity and resilience in challenging environments. By leveraging genetic variability and employing advanced breeding techniques, plant breeders can develop cultivars that not only increase yields but also improve nutritional content, ultimately contributing to food security in regions prone to drought like Iran.

It was observed that traits such as TGW, GY, BY, and SPAD exhibited similar PCV and GCV values under both conditions, indicating that they were less affected by environmental factors and could be consistently chosen for in future breeding programs. The substantial values of PCV, GCV, and *H*^2^ for each experiment, along with GA and GAM under drought stress conditions, have demonstrated significant correlations with traits such as BY and GY in 2020, PH in both years, SPAD in 2021, and TGW in 2020. These variables are essential in elucidating the variability observed in wheat, suggesting that further improvements can be achieved through selective breeding. Conversely, limited progress is expected for RWC due to its low genetic advance value. Strong selection for quantitative traits, even with high heritability, based on a single testing environment, may prove to be unreliable due to significant genotype-environment interactions and high error variance^86^. Pankaj et al.^87^ also emphasized the role of genotype × environment interaction (G × E) in wheat under stress and used METs with multiple sowing dates to reliably identify stable genotypes, supporting the need for multi-environment testing in selection. Results of our study highlights the importance of certain traits in wheat breeding programs, emphasizing the need for careful consideration of environmental factors and genetic variability. By focusing on traits with high heritability and consistent performance across different conditions, breeders can make more informed decisions to enhance wheat productivity and quality. Additionally, understanding the limitations of certain traits, such as RWC, can help researchers prioritize resources and efforts towards traits that offer greater potential for improvement. Ultimately, this research underscores the complexity of wheat genetics and the importance of comprehensive breeding strategies to address the challenges of modern agriculture. Estimates of heritability in breeding trials with non-replicated plots in a single testing environment have consistently shown to be low^88^. This indicates that selecting for productivity using non-replicated trials may not be effective, even when utilizing the best breeding lines from previous years' assessments^89^. In our study, a high estimate of heritability in METs for PH suggests that this trait is less influenced by environmental factors. This finding implies that PH can be leveraged to develop more resilient wheat varieties in arid regions. The high heritability in METs, coupled with high genetic consistency ratios for PH and RWC, indicates a strong genetic determinism and predictable performance, key attributes of phenotypic stability. This aligns with previous research on wheat^90–93^.

### Identifying drought tolerant wheat RILs using different indices

Identifying drought-tolerant cultivars is crucial for breeding for drought tolerance due to the varying adaptability and resistance to drought stress among different genotypes. Utilizing stress tolerance indices is an efficient method to accurately identify tolerant genotypes under stress conditions. In wheat, the stress tolerance index has been widely used in studies to select genotypes capable of withstanding drought^26,34,41,94^. When choosing tolerant genotypes, it is preferable to select those with smaller values of TOL and STI, as larger values indicate a higher susceptibility to stress. However, distinguishing between tolerant and high-yielding genotypes can be challenging using TOL and STI^95^. In our current study, we found a positive and significant correlation between grain yield under well-watered conditions (Yp) and SSI, STI, and TOL over two years. Similarly, a positive and significant relationship was observed between grain yield under stress conditions (Ys) and STI, YSI, and RSI. Therefore, STI was identified as a more reliable indicator of Yp and Ys compared to TOL, SSI, and RSI. Aktaş^96^ also noted similar correlations in drought-resistant wheat genotypes, while Mwadzingeni et al.^97^ suggested that STI could effectively identify high-yielding genotypes in various conditions. Based on various drought tolerance indices such as SSI, STI, YSI, RSI, and TOL, the RILs under investigation were categorized into five distinct clusters. RILs in cluster I demonstrated the highest values for RSI, STI, and YSI, followed by those in cluster II. Conversely, RILs in cluster III showed the lowest values. Among the RILs, numbers 90, 133, 76, 104, and 98 stood out with the most favorable rankings for Ys, TOL, SSI (sensitive indices), YI, YSI, and RSI (Drought yieldperformance) in this study. These specific RILs show promising potential as genotypes suitable for cultivation in agricultural regions that frequently face prolonged periods of water scarcity. They will play a crucial role in further research studies aimed at developing drought-resistant wheat cultivars using traditional breeding techniques such as hybridization, backcrossing, and pedigree analysis. Majidimehr et al.^20^ also reported significant genotypic variation for drought indices in spring bread wheat, noting a high heritability for SSI (0.96) and using PCA and cluster analysis to successfully group 100 genotypes based on their stress response.

The superior and stable performance of the elite RILs, such as 41, 101, and 33, which demonstrated clear transgressive segregation beyond their parental lines (Seri and Babax), validates the effectiveness of our multi-trait, multi-environment selection strategy. While the parents exhibited expected contrasting sensitivity, the elite RILs combined higher yield potential with lower percent reductions under stress. This demonstrates that recombination successfully combined superior allele combinations, achieving genetic gains in both productivity and drought resilience that exceeded the parental range.

### Selection of best-performing RILs based on MGIDI

Traditional multi-environment performance evaluation methods analysis methods based on univariate techniques have been found to be ineffective^54,98,99^. In response, researchers have turned to multivariate techniques to evaluate genotype performance across different environments across different environments^100,101^. As breeding methods continue to evolve, the use of multi-trait selection indexes has gained popularity among plant breeders. Linear indexes are commonly used when considering multiple selection criteria. However, a limitation of linear selection indexes is the collinearity often observed among the traits being evaluated^60^. This collinearity can result in biased coefficients in multiple regression analysis, leading to reduced selection gains. To tackle this issue, Olivoto and Nardino^53^ introduced the MGIDI. This innovative index effectively addresses the challenges associated with collinearity in trait assessment. Recent studies have shown the success of the MGIDI approach in selecting superior genotypes under both biotic and abiotic stress conditions in various crops, such as sesame^102^, wheat^46^, rice^103^, tomato^104^, and rapeseed^57^. Genotypes with lower MGIDI values generally demonstrate superior performance across testing conditions and stress tolerance. The MGIDI offers breeders a targeted approach to improving or reducing specific traits, aligning genotypes with breeding goals. In our study, we analyzed the genetic distance between 167 RILs along with their two parents using an ideotype defined as the MGIDI. This allowed us to establish a selection process that eliminates the need for weighting coefficients and addresses concerns about multicollinearity. This multivariate approach, provides a more comprehensive assessment of stability by simultaneously considering multiple traits and their interactions across environments, effectively capturing the complexity of G × E interaction and identifying genotypes with broad adaptability^46^. Through this method, we identified five top-performing RILs (33, 48, 77, 101, and 140) that consistently outperformed others over two years. Our research highlighted that the selection differential percentage (SDs%) in MGIDI rankings for traits related to GY, BY, and RWC exhibited significant variability among genotypes. This suggests the potential for breeding drought-tolerant wheat. Overall, the development of this new index represents a significant advancement in genotype evaluation methodology analysis, providing a more efficient and effective approach for plant breeders to assess multi-trait performance across environments.

#### Identification of tolerant RILs by DRI

The DRI is a crucial tool used to assess a plant's ability to withstand drought conditions through field experiments. It is particularly valuable in comparing different genotypes with varying phenology across different crops, making it an essential component in crop breeding programs^94,105^. Several RILs exhibited high DRI for yield and its components. The RIL 166 was identified as particularly outstanding, combining high DRI for both HI and GY, indicating an efficient movement of assimilates to the grain under drought. Similarly, the cluster of RILs including RILs 26, 51, 70, 139, 149, and 154 showed significant resilience in BY, suggesting strong biomass production stability. Given that 153 out of 167 RILs along with their two parents showed positive DRI for SPAD in the second year suggests a population-level bias towards some degree of drought tolerance, possibly inherited from the resilient parent. However, the critical insight lies in identifying those lines that combine positive DRI across multiple traits, especially yield. The superior performance of lines like RIL 12 (consistently high for RWC and GY), RIL 166 (for HI and GY), and RIL 26 (for TGW and BY) underscores the importance of selecting for integrated drought tolerance mechanisms rather than single traits. The ranking of lines based on the DRI index closely aligns with that of the MGIDI, underscoring the significant role of DRI in selecting genotypes under stress conditions. This finding is supported by a previous study conducted by Salami et al.^57^, which also emphasized the strong correlation between MGIDI and DRI as predictors of potential under drought stress. This evaluation approach could serve as a valuable reference point for studying the mechanisms of drought tolerance in wheat and developing drought-tolerant wheat cultivars.

## Conclusions

Our results indicated that leaf chlorophyll concentration and RWC were critical for breeding drought-tolerant varieties. High heritability for thousand grain weight and grain yield suggested higher contribution of genetic variance in the phenotypic variations over two seasons and that these traits were less influenced by environmental variation and showed reasonable stability across environments, as supported by their moderate genetic consistency ratios and the identification of RILs with high rank consistency (e.g., RILs 41, 101). These traits can be leveraged to develop drought resilient varieties in arid regions. The STI of RILs with significant variability for each grain and biological yields and RWC represented good potential for selection in drought-tolerance breeding programs. The RILs 21, 23, 25, 48, 54, 64, 70, 77, 90, 139, 149, and 162, which selected as the ideotype drought-tolerant lines demonstrated superior stability through integrated analysis (MGIDI, DRI, cluster analysis). RILs such as 41, 33, 101, and 95 demonstrated transgressive segregation, achieving higher yield potential and significantly greater drought tolerance than either parental line (Seri and Babax), showing genetic gain from the breeding program. These lines have the potential to enhance drought tolerance in future wheat breeding programs. Furthermore, such lines might serve as valuable genetic resources to be utilized to identify the molecular networks responsible for drought tolerance. The MGIDI and DRI were identified as the most reliable indices for predicting drought tolerance in wheat.

## Methods

### Experimental design and treatment details

The study involved the evaluation of 167 recombinant inbred lines (RILs) along with their two parents (SeriM82 and Babax), developed from the cross SeriM82 × Babax evaluated in the Research Farm of Plant Production and Genetics in Bajgah, Shiraz, Iran (29°435N, 52°3528E) during two consecutive cropping seasons, 2020–2021 and 2021–2022. SeriM82 is a semi-dwarf spring wheat variety derived from the “Veery” cross (KVZ/BUHO//KAL/BB), while Babax was released from the “Babax” cross program (BOW/NAC//VEE/3/BJY/COC)^106,107^. SeriM82 carries the 1BL/1RS (rye) translocation and is known for its high yield potential, whereas Babax is recognized as a drought-tolerant variety^107^. The RILs were planted under well-watered and drought stress conditions, each in an *α*-lattice design with three replications. Planting dates was on November 4th and 6th in the first and second years, respectively, with harvests occurring on June 30th and July 6th in the first and second years, respectively. Each plot consisted of two rows of 1.5 m with a 30 cm spacing. During the growing season, fertilizers were applied strategically to maximize crop yield. Specifically, 150 kg ha^−1^kgha diammonium phosphate was used at the beginning of cultivation as an under-cultivation, and 200 kg ha^−1^urea was used at the beginning of tillering and stem elongation. In terms of irrigation, strip irrigation was implemented with drippers spaced 10 cm apart, delivering 1.8 to 2 L water per hour at a pressure of 1 bar.

### Drought stress quantification

To characterize the drought stress environment, meteorological data including monthly rainfall and the corresponding wheat growth stages based on the Zadoks decimal scale^108^ were recorded daily at an on-site weather station (Supp. Table 1). Over the two growing seasons, total rainfall from planting to harvesting time was 190.5 mm in 2020–2021 and 153.5 mm in 2021–2022. The drought stress treatment was imposed by withholding irrigation at the booting stage of wheat (Zadoks code Z45)^108^ on 27 April in both seasons. Rainfall after the start of drought stress was very limited in both growing seasons. In the 2020–2021 season, only two rainfall events occurred between 27 April and 30 June, with a total precipitation of 1.5 mm (0.5 mm on 30 April and 1 mm on 3 May). These events occurred during the transition from the booting stage to the heading and flowering stages. In the 2021–2022 season, two rainfall events were recorded after 27 April, totaling 1 mm (0.5 mm on 7 May and 0.5 mm on 8 May), which also coincided with the booting to early heading stages of crop development. Overall, precipitation during the stress period in both seasons was extremely low (≤ 2 mm). The Drought Intensity Index (DII), equivalent to Stress Intensity (SI), was calculated for each year and combined as^109^:

$$ {\mathrm{DII}}\, = \,{1}\left( {{\overline{\mathrm{Y}}}_{{\mathrm{s}}} /{\overline{\mathrm{Y}}}_{{\mathrm{p}}} } \right) $$

where ${\overline{\mathrm{Y}}}_{{\mathrm{s}}}$ and ${\overline{\mathrm{Y}}}_{{\mathrm{p}}}$ are the mean grain yields of all 167 RILs along with their two parents under drought stress and well-watered conditions, respectively. The DII was 0.25 in the first year and 0.27 in the second year, with a combined mean of 0.26. According to standard classifications^110^, where 0.25 ≤ DII < 0.40 indicates moderate stress, the imposed treatment created a reproducible moderate terminal drought stress, resulting in a significant average yield reduction of 26% across the population.”

### Relative water content (RWC)

The RWC was measured by randomly selecting the mature wheat flag leaves in each plot and treatment. Flag leaves were sampled at the anthesis stage based on the Zadoks decimal code (code Z65)^108^. The leaves were weighed, and quickly immersed in distilled water overnight to saturation. After moving the excess water, the leaves were weighed again (turgor weight) and then oven-dried at 70 °C until a constant weight was achieved. The dry weight was measured and RWC was calculated using the formula^109^:

$$ RWC = \frac{{\left( {FW - DW} \right)}}{{\left( {TW - DW} \right)}} \times 100 $$

where, FW (g) is the fresh weight of the leaf just removed from the plant; DW (g) is the weight of the leaf oven-dried at 70 °C; and TW (g) is the turgor weight of the leaf after saturation.

### Estimation of chlorophyll using SPAD

A chlorophyll meter (SPAD-502, Minolta, Japan) was used to obtain readings for leaf chlorophyll concentration (SPAD value). One month after implementation of drought stress, five plants from each plot were randomly selected, and SPAD values were recorded from the middle of the upper surface of fully matured flag leaves.

### Agronomic and yield components

At the harvest stage, plant height (PH; cm) was recorded from the ground to the tip of the main spike at the physiological maturity stage. For thousand grain weight (TGW; g), 10 randomly selected plants were harvested from the central section of each two-row plot to avoid border effects in each plot. Plants from both rows were harvested for biological yield (BY; kg m^−2^), and grain yield (GY; kg m^−2^). Harvest index (%) was calculated using the equation:

### Analysis of variance (ANOVA) and estimation of genetic variation and genetic gain

The data underwent a combined analysis of variance (c-ANOVA) for treatments and years using the Statistical Analysis System (SAS) software, version 9.3^111^. The variance component and genetic variability, including broad sense heritability (*H*^*2*^) were estimated to determine the genetic and environmental effects on variability of the measured quantitative traits.

Variance components were estimated from the mean squares (MS) of source of variations in the analysis of variance (ANOVA)^112^. Then components of variance including error variance (VE), genotypic variance (VG), and phenotypic variance (VP) were estimated using the following formula:

$$ VE = MSE $$

$$ VG = \left( {\left. {\frac{{\left. {({\mathrm{MSG}} - {\mathrm{MSE}}} \right)}}{r}} \right) \times \left( {100} \right)} \right. $$

$$ VP = VG + VE $$

where, $$VG $$ is the genotypic variance; MSG is the mean square for genotype; MSE is the error mean square in ANOVA and *r* is the number of replications.

The phenotypic coefficient of variation (PCV) and genotypic coefficient of variation (GCV) were calculated and expressed as a percentage^113^. Meanwhile PCV and GCV were classified into three classes; less than 10% (Low), 10–20% (Moderate) and more than 20% (High).

$$ PCV = \left( {\left. {\frac{{\left. {\sqrt {VP} } \right)}}{Mean}} \right) \times \left( {100} \right)} \right. $$

$$ GCV = \left( {\left. {\frac{{\left. {\sqrt {VG} } \right)}}{Mean}} \right) \times \left( {100} \right)} \right. $$

Broad-sense heritability (*H*^*2*^) calculated per environment (within each irrigation regime-year combination) and across all four environments (combined analysis). *H*^2^ for individual environments was estimated as the ratio of genetic variance to the phenotypic variance^114^ as follows:

$$ H^{2} = \left( {\left. {\frac{VG}{{VP}}} \right) \times \left( {100} \right)} \right. $$

*H*^*2*^ across-environment was estimated for traits as follows:

$$ { }H^{2} = \left( {\left. {\frac{VG}{{VG + \frac{VGE}{E} + \frac{VE}{{RE}}\user2{ }}}} \right) \times \left( {100} \right)} \right. $$

where VGE is the genotype × environment variance, and R and E are the number of replications and environments, respectively. The heritability categorized (into three classes: 0–30% (Low), 31–60% (Medium) and more than 60% (High)^115^.

Genetic advance (GA) and GA as a percent of traits mean (GAM) were estimated using the following formula^116^:

$$ GA = \left( {\left. {\frac{{K*H^{2} *VP}}{Mean}} \right) \times \left( {100} \right)} \right. $$

$$ GAM\left( \% \right) = \left( {\left. {\frac{GA}{{Mean}}} \right) \times \left( {100} \right)} \right. $$

where K = 2.06 at 5% selection intensity, *H*^*2*^ = heritability, and VP = phenotypic variance. Meanwhile, GAM was categorized to three classes: less than 10% (Low), 10–20% (Moderate) and more than 20% (High).

### Analysis of genotype × environment interaction

To characterize the significant G × E interaction, rank consistency analysis was performed for grain yield across the four environments (well-watered _2020, Drought_2020, well-watered 2021, Drought_2021). For each RIL, the mean yield in each environment was ranked relative to all other RILs (1 = highest yield, 169 = lowest). The consistency of each genotype's performance was assessed by calculating the standard deviation (SD) of its ranks across environments, with lower SD values indicating more consistent ranking.

### Estimation of consistency ratio for agronomic traits

For each trait, variance components were estimated using mean squares (MS) from the Type III Sum of Squares in the provided ANOVA tables. he genotypic variance (VG) and genotype-by-year interaction variance (VGE) were estimated from the mean squares derived from the Type III Sum of Squares in the ANOVA tables for each trait. The calculations followed standard formulas for a balanced mixed model, where genotypes and the genotype-by-year interaction were considered random effects. The following equations were applied:

Genotypic variance (VG):

$$ \sigma_{G}^{2} = { }\frac{{MS_{Genotype } - MS_{Year \times Genotype } }}{r \times y} $$

Genotype × year interaction variance (VGE):

$$ \sigma_{G \times Y}^{2} = { }\frac{{MS_{Year \times Genotype } - MS_{Error } }}{r} $$

Consistency ratio:

$$ Consistency ratio = { }\frac{{\sigma_{G}^{2} }}{{\sigma_{G}^{2} + \sigma_{G \times Y}^{2} }} $$

where, $\sigma_{G}^{2}$ is the estimated genotypic variance component, $MS_{{Genotype{ }}}$ is the mean square for the genotype effect, $MS_{{Year{ } \times { }Genotype{ }}}$ is The Mean Square for the Genotype-by-Year interaction effect, $MS_{{Year{ } \times { }Genotype{ }}} - { }MS_{{Error{ }}}$ is The Mean Square for the residual Error term, r: The number of replications (blocks) per unique environment, y: The number of years (environments), $\sigma_{G}^{2}$ is The estimated Genotypic Variance component, $\sigma_{G \times Y}^{2}$ is The estimated Genotype-by-Year Interaction Variance component.

### Drought tolerance and susceptibility indices

Indices for drought stress tolerance, such as the Stress Susceptibility Index (SSI), Stress Tolerance Index (STI), Yield Stability Index (YSI), Relative Stress Index (RSI), and Tolerance Index (TOL), were calculated to identify tolerant RILs. The data for trait values under control conditions (*Yp*), traits under water deficit treatment (*Ys*), trait means under control conditions (*Ȳp*), and trait means under water deficit treatment (*Ȳs*) were used to calculate the five-drought tolerance and susceptibility indices through the following Eqs.^110,117–120^:

$$ SSI = \frac{{\left[ {1 - \left( {YS - YP} \right)} \right]}}{{\left[ {1 - \left( {\overline{Y}S - \overline{Y}P} \right)} \right]}} $$

$$ STI = \frac{{\left( {YS \times YP} \right)}}{{\overline{Y}P^{2} }} $$

$$ YSI = \frac{YS}{{YP}} $$

$$ RSI = \frac{{\left( {YS/YP} \right)}}{{\left( {\overline{Y}S/\overline{Y}P} \right)}} $$

$$ TOL = \left( {YP - YS} \right) $$

### The multi-trait genotype-ideotype distance index (MGIDI)

The MGIDI was calculated by measuring the Euclidean distance between treatment scores and the ideal treatment. The formula for MGIDI is as follows^53^:

$$ MGIDI_{i} = \left[ {\mathop \sum \limits_{j = 1}^{f} \gamma ij - \gamma j^{2} } \right]^{0.5} $$

where, *γ*_*ij*_ represents the score of the *i*th accession in the *j*th factor (*i* = 1, 2,…, *t*; *j* = 1,2,…,*f* ), with *t* and *f* stand for the number of accessions and factors, respectively; and *γ*_*j*_ is the *j*th score of the ideal accession. A lower MGIDI value indicates that an accession is closer to the ideal accession, reflecting desirable values for all calculated indices. The selection differential for all traits was determined using a selection intensity of approximately 10%. Data manipulation and computations for indices were performed using the 'metan' package in the R program^53^.

### Factor analysis (FA)

The FA was used to rank RILs based on desired trait values using the MGIDI data. Initially, FA was performed using *r*X*ij* to analyze the correlation structure and reduce the dimensionality of the data. This process provided a more comprehensive understanding of the relationships between RILs and tested traits^53^. The FA was calculated as follows,

$$ X = \mu + Lf + \varepsilon $$

where, *X* represents a *p* × 1 vector of rescaled observations, μ is a *p* × 1 vector of standardized means, *L* is a *p* × *f* matrix of factorial loadings, *f* is a *p* × 1 vector of common factors, and *ε* is a *p* × 1 vector of residuals. Here, *p* and *f* represent to the number of traits and common factors retained, respectively. The eigenvalues and eigenvectors are obtained from the correlation matrix of *r*X*ij*. Initially, loadings are determined by considering only factors with eigenvalues greater than one. Subsequently, the varimax rotation criteria^121^ were applied for the analytic rotation and estimation of final loadings. Finally, scores were computed as follows:

$$ F = Z\left( {A^{T} R^{ - 1} } \right)^{T} $$

where, *F* is a *g* × *f* matrix containing the factorial scores; *Z* is a *g* × *p* matrix with the rescaled standardized means; *A* is a *p* × *f* matrix of canonical loadings; and *R* is a *p* × *p* correlation matrix between the traits. Here, *g*, *f*, and *p* represent the number of treatments, factors retained, and traits, respectively. The decision on the number of factors retained was determined using the Guttman-Kaiser criterion^122^. This criterion follows the rule of eigenvalues greater than unit.

### Strengths and weaknesses

The proportion of the MGIDI attributed to the *i*th treatment explained by the *j*th factor (ω*ij*) was utilized. To evaluate the rank of each selected RILs as outlined below^53^:

$$ \omega ij = \frac{{\sqrt {D_{ij}^{2} } }}{{\mathop \sum \nolimits_{j = 1}^{f} D_{ij}^{2} }} $$

where *Dij* represents the distance between the *i*th RIL and the ideal RIL for the *j*th factor. A low contribution of a factor suggests that the characteristics linked to that factor closely match the desired RIL.

### Drought resistance index (DRI)

The DRI was determined by analyzing the SPAD, RWC, PH, TGW, HI, BY, and GY scores in both 2020 and 2021, under drought stress and non-stress conditions. The DRI was calculated as follows^123^:

$$ DRI = \frac{{\left( {Ys/Yn} \right)}}{{\left( {Ms/Mn} \right)}} $$

where *Ys* and *Yn* represent the values of measured traits in each genotype under stress and non-stress conditions, respectively. Similarly, *Ms* and *Mn* denote the mean traits across all genotypes under stress and non-stress conditions, respectively.

### Statistical analysis

Descriptive statistics (means and standard deviations), were calculated using the Statistical Analysis System (SAS, Version 9.3, SAS Institute Inc., Cary, NC, USA). The PROC GLM procedure was used for c-ANOVA in SAS software^111^. An analysis of variance was performed for the alpha lattice design using the R software package agridat. To compare the distribution of data between well-watered and drought stress groups over two years, violin plots were generated using R software version 3.4.0 and the ggplot2 package. Principal components analysis (PCA) was conducted to visualize the relationships between traits and similarities of wheat RILs under two experimental conditions in R version 3.4.0 (http://www.r-project.org/). The correlation matrix between variables and construction of a heatmap of correlation coefficients were computed using packages plotly, heatmaply, and ggcorrplot (https://cran.r-project.org/web/packages/ggcorrplot/index.html) in the R program. A phylogenetic tree for the wheat RILs under two experimental conditions was created using the R package ape^124^.

### Comprehensive evaluation value (*D*)

In order to assess the impact of dimension, the drought tolerance coefficient (DTC) was calculated for all traits. This process involved comparing the performance of each RIL under drought stress and well-watered conditions^125^. A Principal Component Analysis (PCA) was then conducted to determine the DTC value of all traits, which involved obtaining the eigenvalues, percentage variability, and cumulative variance proportion of each principal component (PC). Generally, the PCs with eigenvalues ≥ 1 were selected^126^. The membership function value (*Ui*) was calculated based on the comprehensive score value from the i^th^ principal component, providing a holistic evaluation of drought tolerance for multiple traits^127^. Subsequently, the *D* value was calculated using *Ui* and weight value (*Wi*), which allowed for the grouping of wheat RILs as drought tolerant, moderate, or sensitive^128^. The calculations for the DTC, *Ui*, and *D* were carried out as follows:

$$ DTC = \left( {\left. {\frac{XDS}{{XCK}}} \right) \times \left( {100} \right)} \right. $$

$$ Wi = \frac{Pi}{{\mathop \sum \nolimits_{i}^{n} Pi}} , i = 1, 2, 3, \ldots .. , n $$

$$ Ui = \frac{{Ti - T{\mathrm{min}}}}{{T{\mathrm{max}} - T{\text{min }}}} , i = 1, 2, 3, \ldots .. , n $$

$$ D = \mathop \sum \limits_{i = 1}^{n} \left[ {Ui \times Wi} \right] , i = 1, 2, 3, \ldots .. , n $$

where *X*_*DS*_ and *X*_*CK*_ represent the trait values of the tested RIL under drought stress (DS) and well-watered treatment (CK), respectively. *Wi* stands for the weight of the *i*_*th*_ principal component, *Pi* indicates the contribution rate of the *i*_*th*_ principal component, and *Ui* represents the membership function value of the *i*_*th*_ principal component. *Ti*, *T*max, and *T*min correspond to the comprehensive score, maximum, and minimum values of the *i*_*th*_ principal component, respectively. *D* is the comprehensive evaluation index of drought for the 167 RILs along with their two parents . It is important to note that the higher the *D* value, the stronger the drought tolerance of the wheat varieties.

## Supplementary Information

Below is the link to the electronic supplementary material.

Supplementary Material 1

## Data Availability

Data will be made available on request.
