# Supplementary material for: Identification of drought-tolerant ideotype in wheat using multi-trait genotype-ideotype distance index (MGIDI) and comprehensive evaluation value
Source: Sci Rep. 2026 Apr 17;16:17882. doi: 10.1038/s41598-026-46140-6 (PMC13250071; doi:10.1038/s41598-026-46140-6)
Supplement: Supplementary file 1 — Supplementary Material 1 [file 41598_2026_46140_MOESM1_ESM.docx]

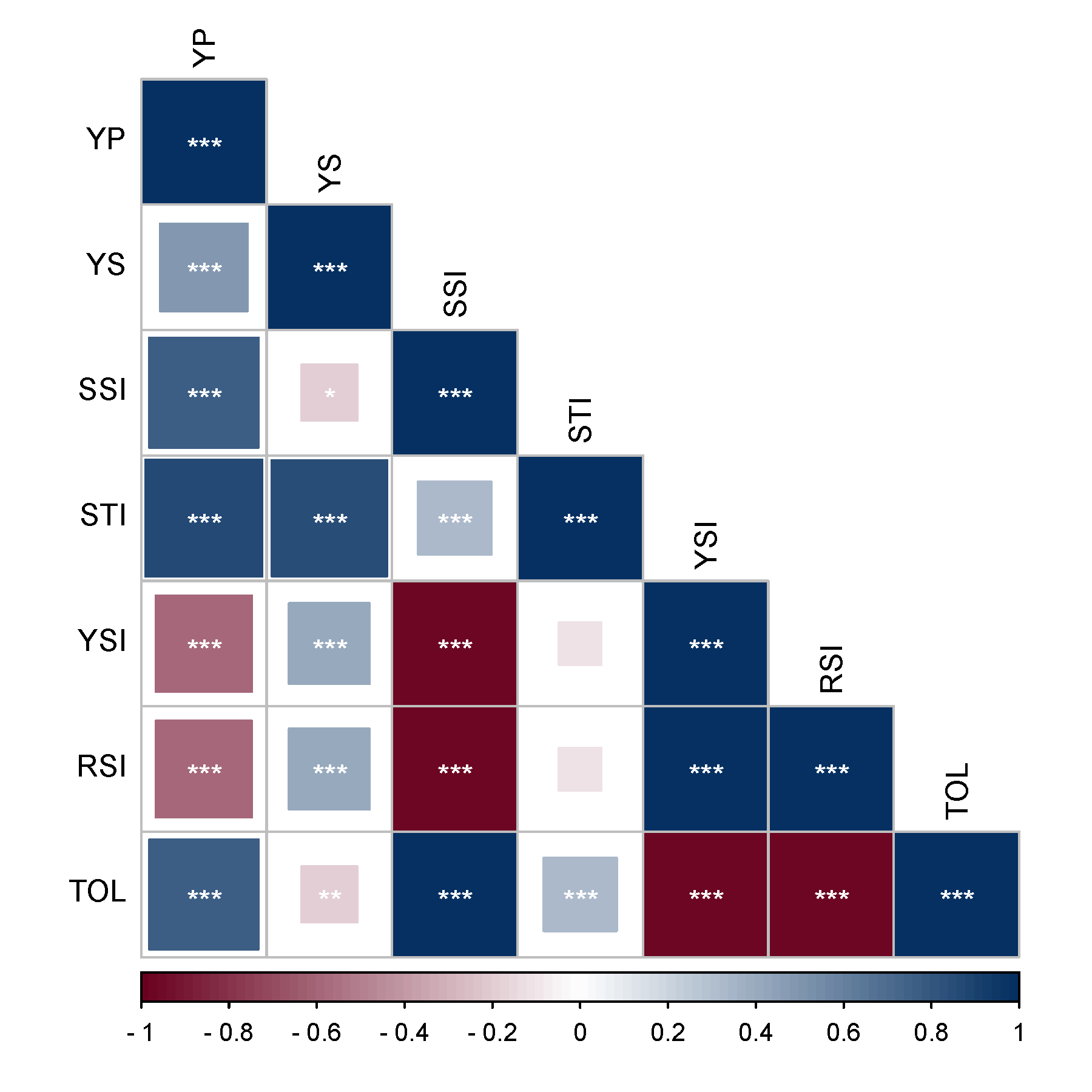

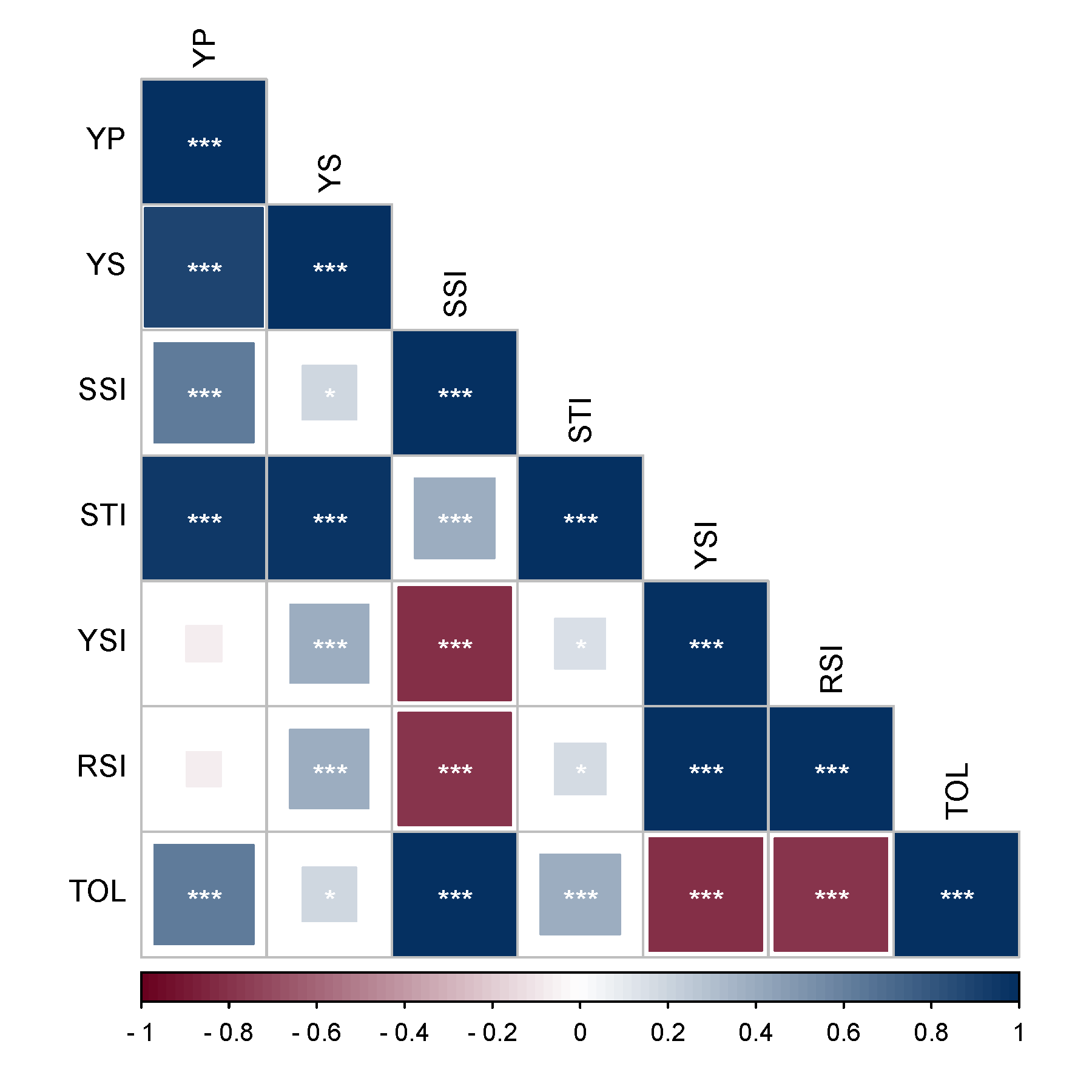


**A**

**B**

**FIGURE S1.** Correlation coefficients and diagram between grain yield under non-stress (YP), and drought stress condition (YS) along with five indices values for the 169 wheat RILs, in 2020-2021 (**A**), 2021-2022 (**B**). SSI, STI, YSI, RSI, and TOL were the abbreviations of stress susceptibility index, stress tolerance index, yield stability index, relative stress index, tolerance index respectively.

| TABLE S1. Meteorological data for the growing seasons recorded at the on-site weather station. | | | |
| --- | --- | --- | --- |
| Meteorological Parameter | Wheat Growth Stage  (Zadoks scale) | 2020-2021 Season  (4 Nov – 30 Jun) | 2021-2022 Season  (6 Nov – 6 Jul) |
| Total Rainfall | Whole growing season | 190.5 mm | 153.5 mm |
| Temperature Range (Min – Max) | Whole growing season | -5.6°C – 39.5°C | -9°C – 40°C |
| Relative Humidity Range (Min – Max) | Whole growing season | 7% – 97% | 0% – 100% |
| Monthly Rainfall (mm) |  |  |  |
| November | Germination – Seedling (Z00–Z13) | 65.5 | 5.0 |
| December | Early tillering (Z20–Z23) | 63.5 | 39.5 |
| January | Tillering (Z21–Z25) | 0.0 | 78.5 |
| February | Late tillering (Z25–Z29) | 47.5 | 20.5 |
| March | Stem elongation (Z30–Z32) | 8.5 | 1.5 |
| April | Stem elongation – Booting (Z33–Z49) | 4.5 | 7.5 |
| May | Heading – Flowering (Z50–Z69) | 1.0 | 1.0 |
| June | Grain filling – Ripening (Z70–Z90) | 0.0 | 0.0 |
| July | Late ripening / harvest | – | 0.0 |
| Total Rainfall: Cumulative precipitation from planting to harvest date. Ranges: The minimum and maximum daily values recorded over the entire season for temperature and relative humidity. | | | |

| TABLE S2. Yield reduction percentages of 169 recombinant inbred lines (RILs) under drought stress in two consecutive growing seasons (2020-2021 and 2021-2022) | | | | | |
| --- | --- | --- | --- | --- | --- |
| RIL | 2020-2021 | 2021-2022 | RIL | 2020-2021 | 2021-2022 |
| 1 | 41.06 | 5.26 | 86 | 23.08 | 31.38 |
| 2 | 33.65 | 21.37 | 87 | 28.52 | 42.74 |
| 3 | 36.59 | 12.07 | 88 | 42.04 | 28.50 |
| 4 | 40.51 | 15.93 | 89 | 31.58 | 27.84 |
| 5 | 31.38 | 30.30 | 90 | 22.78 | 35.04 |
| 6 | 27.50 | 30.85 | 91 | 28.76 | 27.65 |
| 7 | 41.51 | 31.43 | 92 | 24.51 | 49.14 |
| 8 | 36.82 | 27.47 | 93 | 17.85 | 26.16 |
| 9 | 23.54 | 15.28 | 94 | 36.85 | 23.68 |
| 10 | 23.78 | 37.50 | 95 | 23.00 | 29.32 |
| 11 | 50.18 | 7.87 | 96 | 42.38 | 28.30 |
| 12 | 46.75 | 36.82 | 97 | 48.80 | 40.20 |
| 13 | 37.27 | -8.67 | 98 | 20.78 | 52.76 |
| 14 | 37.74 | 17.39 | 99 | 11.18 | 43.71 |
| 15 | 35.68 | 15.59 | 100 | 39.30 | 30.56 |
| 16 | 35.59 | 32.94 | 101 | 22.75 | 37.32 |
| 17 | 25.50 | 54.26 | 102 | 30.54 | 20.20 |
| 18 | 36.31 | 41.07 | 103 | 31.74 | 21.98 |
| 19 | 51.43 | 12.00 | 104 | 82.65 | 15.76 |
| 20 | 37.26 | 16.50 | 105 | 31.75 | 27.78 |
| 21 | 30.20 | 30.99 | 106 | 24.25 | 14.79 |
| 22 | 38.36 | 36.65 | 107 | 41.64 | 36.71 |
| 23 | 40.18 | 26.42 | 108 | 24.94 | 10.39 |
| 24 | 43.29 | 26.84 | 109 | 30.07 | 37.74 |
| 25 | 38.66 | 28.53 | 110 | 34.53 | 36.06 |
| 26 | 36.46 | 48.26 | 111 | 28.06 | 29.73 |
| 27 | 28.10 | 59.13 | 112 | 33.37 | 28.25 |
| 28 | 31.64 | 18.35 | 113 | 23.62 | 34.97 |
| 29 | 31.91 | 22.46 | 114 | 23.73 | 26.88 |
| 30 | 24.18 | 16.04 | 115 | 27.08 | 41.55 |
| 31 | 37.32 | 13.89 | 116 | 33.65 | 30.00 |
| 32 | 30.14 | 13.40 | 117 | 23.99 | 36.22 |
| 33 | 15.43 | 10.14 | 118 | 28.48 | 43.50 |
| 34 | 26.32 | 20.62 | 119 | 42.28 | 23.81 |
| 35 | 24.22 | 15.93 | 120 | 46.79 | 51.74 |
| 36 | 25.99 | 23.91 | 121 | 50.79 | 53.53 |
| 37 | 36.66 | 14.49 | 122 | 16.84 | 27.50 |
| 38 | 43.07 | 9.37 | 123 | 34.88 | 15.62 |
| 39 | 26.63 | 44.60 | 124 | 22.06 | 15.83 |
| 40 | 24.70 | 9.94 | 125 | 27.19 | 65.35 |
| 41 | 18.39 | 6.03 | 126 | 31.30 | 20.35 |
| 42 | 26.92 | 21.28 | 127 | 28.58 | 34.11 |
| 43 | 36.90 | 14.14 | 128 | 33.90 | 12.93 |
| 44 | 72.76 | 25.94 | 129 | 28.36 | 39.42 |
| 45 | 36.22 | 15.31 | 130 | 27.77 | 26.32 |
| 46 | 29.66 | 11.90 | 131 | 29.59 | 18.21 |
| 47 | 27.45 | 25.00 | 132 | 29.82 | 38.26 |
| 48 | 26.60 | 26.74 | 133 | 21.38 | 28.99 |
| 49 | 27.78 | 20.35 | 134 | 28.24 | 32.21 |
| 50 | 45.63 | 33.78 | 135 | 31.60 | 21.70 |
| 51 | -0.98 | 37.75 | 136 | 26.84 | 16.34 |
| 52 | 32.68 | 8.04 | 137 | 22.44 | 52.57 |
| 53 | 34.44 | 25.80 | 138 | 33.07 | 33.04 |
| 54 | 25.50 | 22.36 | 139 | 24.55 | 36.80 |
| 55 | 34.16 | 15.23 | 140 | 26.44 | 41.03 |
| 56 | 36.07 | 49.46 | 141 | 32.18 | 27.50 |
| 57 | 75.26 | 28.08 | 142 | 23.92 | 38.58 |
| 58 | 24.69 | 43.07 | 143 | 29.65 | 27.59 |
| 59 | 21.61 | 28.34 | 144 | 21.76 | 22.91 |
| 60 | 28.62 | 31.21 | 145 | 28.86 | -0.96 |
| 61 | 73.37 | 35.86 | 146 | 33.79 | 21.77 |
| 62 | 32.12 | 16.30 | 147 | 25.56 | 69.13 |
| 63 | 17.79 | 38.84 | 148 | 72.90 | 33.53 |
| 64 | 24.37 | 47.15 | 149 | 22.31 | 39.23 |
| 65 | 68.07 | 15.54 | 150 | 26.67 | 35.34 |
| 66 | 25.76 | 28.38 | 151 | 20.00 | 16.30 |
| 67 | 34.58 | 45.47 | 152 | 19.92 | 27.74 |
| 68 | 26.41 | 29.41 | 153 | 45.89 | 77.70 |
| 69 | 26.76 | 30.70 | 154 | -8.84 | 27.94 |
| 70 | 26.50 | 26.86 | 155 | 25.88 | 15.15 |
| 71 | 35.90 | 40.82 | 156 | 30.63 | 69.31 |
| 72 | 25.56 | 27.84 | 157 | 32.93 | 38.29 |
| 73 | 32.71 | 30.96 | 158 | 30.59 | 30.43 |
| 74 | 49.81 | 23.63 | 159 | 24.80 | 18.22 |
| 75 | 25.42 | 24.95 | 160 | 35.77 | 52.28 |
| 76 | 19.30 | 31.95 | 161 | 22.96 | 25.66 |
| 77 | 17.92 | 31.59 | 162 | 24.87 | 22.16 |
| 78 | 32.61 | 22.67 | 163 | 22.86 | 25.89 |
| 79 | 35.19 | 33.38 | 164 | 33.18 | 17.75 |
| 80 | 40.76 | 39.25 | 165 | 26.73 | 32.06 |
| 81 | 20.64 | 28.78 | 166 | 25.58 | 65.54 |
| 82 | 23.76 | 40.07 | 167 | 27.10 | 30.87 |
| 83 | 31.03 | 34.63 | 168 | 32.90 | 37.07 |
| 84 | 19.89 | 21.07 | 169 | 30.48 | 29.70 |
| 85 | 30.62 | 37.27 |  |  |  |

| TABLE S3. Performance and ranking consistency of top 10 RILs for grain yield across four environments | | | | | | | | | | | |
| --- | --- | --- | --- | --- | --- | --- | --- | --- | --- | --- | --- |
| RIL | Mean SY | E1 (WW_20) | Rank | E2 (DS_20) | Rank | E3 (WW_21) | Rank | E4 (DS_21) | Rank | Rank SD | Consistency |
| 12 | 445.1 | 523.0 | 1 | 293.3 | 8 | 403.3 | 27 | 288.7 | 33 | 15.2 | Moderate |
| 101 | 429.6 | 436.3 | 5 | 370.0 | 1 | 482.7 | 5 | 324.0 | 13 | 5.0 | High |
| 159 | 392.4 | 421.0 | 8 | 338.3 | 3 | 378.7 | 44 | 292.7 | 29 | 20.1 | Moderate |
| 41 | 391.8 | 404.7 | 9 | 337.0 | 4 | 470.0 | 8 | 398.7 | 3 | 2.9 | Very High |
| 82 | 386.7 | 265.0 | 74 | 230.3 | 43 | 585.3 | 1 | 392.0 | 4 | 34.7 | Low |
| 95 | 381.6 | 354.7 | 23 | 306.0 | 6 | 593.3 | 2 | 411.3 | 1 | 10.2 | High |
| 142 | 369.6 | 383.3 | 15 | 322.7 | 5 | 446.0 | 14 | 300.0 | 20 | 6.4 | High |
| 30 | 364.3 | 448.7 | 3 | 335.7 | 2 | 352.7 | 65 | 306.0 | 18 | 32.8 | Low |
| 66 | 363.0 | 387.7 | 13 | 295.7 | 7 | 408.7 | 23 | 314.0 | 12 | 7.4 | High |
| 5 | 352.2 | 346.7 | 27 | 236.0 | 38 | 478.0 | 6 | 361.3 | 7 | 16.1 | Moderate |
| WW_20: Well-watered conditions during the 2020-2021 growing season. DS_20: Drought stress conditions during the 2020-2021 growing season. WW_21: Well-watered conditions during the 2021-2022 growing season. DS_21: Drought stress conditions during the 2021-2022 growing season. Ranks are from 1 (highest yield) to 169 (lowest yield) within each environment. Rank SD = standard deviation of ranks across environments. Stability classification: Very High (SD < 5), High (5 ≤ SD < 15), Moderate (15 ≤ SD < 25), Low (SD ≥ 25). | | | | | | | | | | | |

| TABLE S4. Genotypic and genotype-by-year interaction variance components and consistency ratio for key agronomic traits | | | |
| --- | --- | --- | --- |
| Trait | VG | VGE | Consistency Ratio |
| PH | 347.91 | 15.08 | 0.958 |
| RWC | 0.0137 | 0.00 | 1.000 |
| TGW | 4.84 | 3.85 | 0.557 |
| GY | 1208.62 | 3554.28 | 0.254 |
| BY | 7447.40 | 38623.20 | 0.162 |
| SPAD | 1.94 | 10.89 | 0.151 |
| HI | 0.50 | 7.24 | 0.064 |
| PH: plant height, RWC: relative water content, TGW: thousand grain weight, GY: grain yield, BY: biomass yield, SPAD: chlorophyll index (SPAD value), HI: harvest index. VG: genotypic variance, VGE (genotype × year interaction variance). consistency ratio (VG/(VG+VGE)). | | | |

| TABLE S5. Eigen vectors and eigen values of the principal components (PC) for various morphophysiological traits in 169 wheat accessions at well-watered and drought stress conditions across two years (2020-2021 and 2021-2022). | | | | | | | |
| --- | --- | --- | --- | --- | --- | --- | --- |
| Well-watered condition |  |  |  |  |  |  |  |
| Variable | PC1 | PC2 | PC3 | PC4 | PC5 | PC6 | PC7 |
| Eigenvalue | 59230.40 | 1044.86 | 167.33 | 16.38 | 7.2 | 0.5 | 0.002 |
| Variation (%) | 0.97 | 0.01 | 0.002 | 0.0003 | 0.0001 | 0 | 0 |
| Cumulative | 0.97 | 0.99 | 0.99 | 0.99 | 1 | 1 | 1 |
| Grain yield | 0.24 | 0.96 | 0.002 | 0.004 | -0.002 | -0.09 | -0.0002 |
| Biological yield | 0.96 | -0.24 | -0.18 | -0.001 | -0.002 | 0.02 | -0.0002 |
| Harvest index | -0.002 | 0.09 | 0.004 | -0.005 | 0.02 | 0.99 | -0.009 |
| Thousand grain weight | 0.003 | -0.0004 | -0.018 | 0.09 | 0.99 | -0.02 | -0.009 |
| Plant height | 0.01 | -0.007 | 0.99 | 0.05 | 0.01 | -0.003 | -0.0003 |
| Relative water content | 0.0002 | 0.001 | 0.0003 | 0.0006 | 0.0003 | 0.009 | 0.99 |
| Estimation of chlorophyll using SPAD | -0.0006 | -0.003 | -0.05 | 0.99 | -0.09 | 0.007 | -0.0006 |
| Drought stress condition |  |  |  |  |  |  |  |
| Variable | PC1 | PC2 | PC3 | PC4 | PC5 | PC6 | PC7 |
| Eigenvalue | 40882.14 | 438.83 | 126.18 | 12.50 | 5.04 | 0.37 | 0.002 |
| Variation (%) | 0.98 | 0.01 | 0.003 | 0.0003 | 0.0001 | 0 | 0 |
| Cumulative | 0.98 | 0.99 | 0.99 | 0.99 | 1 | 1 | 1 |
| Grain yield | 0.24 | 0.96 | 0.04 | -0.001 | 0.00004 | -0.11 | -0.001 |
| Biological yield | 0.96 | -0.24 | -0.02 | 0.001 | -0.001 | 0.03 | 0.0001 |
| Harvest index | -0.001 | 0.11 | 0.004 | -0.001 | 0.01 | 0.99 | 0.0004 |
| Thousand grain weight | 0.001 | -0.003 | 0.01 | -0.12 | 0.99 | -0.01 | -0.0007 |
| Plant height | 0.01 | -0.05 | 0.99 | 0.03 | -0.007 | 0.001 | 0.0003 |
| Relative water content | 0.0003 | 0.001 | -0.0002 | -0.0007 | 0.0006 | -0.0006 | 0.99 |
| Estimation of chlorophyll using SPAD | -0.001 | 0.003 | -0.03 | 0.99 | 0.13 | -0.0009 | 0.0005 |

| TABLE S6. Calculated drought tolerance and susceptibility indices in 169 wheat accessions based on the chlorophyll estimated using SPAD indices in 2020-2021. | | | | | | | | | | | |
| --- | --- | --- | --- | --- | --- | --- | --- | --- | --- | --- | --- |
| Code | SSI | STI | YSI | RSI | TOL | Code | SSI | STI | YSI | RSI | TOL |
| G1 | 1.45 | 0.58 | 0.54 | 0.80 | 20.86 | G86 | 1.14 | 0.67 | 0.64 | 0.94 | 16.30 |
| G2 | 1.75 | 0.59 | 0.48 | 0.70 | 25.50 | G87 | 0.77 | 0.52 | 0.72 | 1.06 | 10.57 |
| G3 | 0.19 | 0.75 | 0.95 | 1.41 | 1.80 | G88 | 0.74 | 0.84 | 0.78 | 1.15 | 10.17 |
| G4 | 0.86 | 0.63 | 0.71 | 1.05 | 11.97 | G89 | 0.97 | 0.63 | 0.68 | 1.00 | 13.60 |
| G5 | 1.45 | 0.60 | 0.54 | 0.80 | 20.87 | G90 | 0.71 | 0.78 | 0.78 | 1.15 | 9.80 |
| G6 | 1.68 | 0.69 | 0.52 | 0.76 | 24.47 | G91 | 0.57 | 0.74 | 0.82 | 1.21 | 7.63 |
| G7 | 0.55 | 0.66 | 0.81 | 1.20 | 7.30 | G92 | 1.42 | 0.68 | 0.57 | 0.84 | 20.43 |
| G8 | 1.15 | 0.82 | 0.66 | 0.98 | 16.40 | G93 | 1.12 | 0.58 | 0.62 | 0.92 | 15.90 |
| G9 | 0.61 | 0.59 | 0.78 | 1.16 | 8.23 | G94 | 0.59 | 0.76 | 0.81 | 1.20 | 7.97 |
| G10 | 1.01 | 0.91 | 0.71 | 1.05 | 14.23 | G95 | 0.73 | 0.49 | 0.72 | 1.07 | 10.06 |
| G11 | 0.85 | 0.77 | 0.74 | 1.09 | 11.83 | G96 | 0.95 | 0.57 | 0.67 | 0.99 | 13.33 |
| G12 | 1.27 | 0.67 | 0.60 | 0.89 | 18.20 | G97 | 1.57 | 0.75 | 0.55 | 0.82 | 22.70 |
| G13 | 1.31 | 0.53 | 0.56 | 0.82 | 18.74 | G98 | 0.96 | 0.64 | 0.68 | 1.01 | 13.47 |
| G14 | 0.93 | 0.59 | 0.68 | 1.00 | 13.10 | G99 | 0.38 | 0.82 | 0.89 | 1.31 | 4.67 |
| G15 | 0.73 | 0.59 | 0.74 | 1.10 | 10.04 | G100 | 1.24 | 0.72 | 0.62 | 0.92 | 17.70 |
| G16 | 0.84 | 0.66 | 0.72 | 1.06 | 11.76 | G101 | 1.06 | 0.54 | 0.63 | 0.93 | 15.07 |
| G17 | 1.08 | 0.83 | 0.68 | 1.01 | 15.33 | G102 | 1.71 | 0.74 | 0.52 | 0.77 | 24.93 |
| G18 | 1.45 | 0.65 | 0.56 | 0.82 | 20.90 | G103 | 1.40 | 0.74 | 0.59 | 0.87 | 20.10 |
| G19 | 1.02 | 0.68 | 0.67 | 0.99 | 14.36 | G104 | 1.50 | 0.76 | 0.57 | 0.84 | 21.74 |
| G20 | 0.87 | 0.73 | 0.72 | 1.07 | 12.17 | G105 | 0.66 | 0.72 | 0.78 | 1.16 | 9.03 |
| G21 | 1.62 | 0.65 | 0.52 | 0.77 | 23.50 | G106 | 0.52 | 0.74 | 0.83 | 1.23 | 6.90 |
| G22 | 0.81 | 0.58 | 0.71 | 1.05 | 11.27 | G107 | 1.19 | 0.55 | 0.60 | 0.88 | 16.93 |
| G23 | 0.77 | 0.60 | 0.73 | 1.08 | 10.63 | G108 | 1.25 | 0.72 | 0.62 | 0.92 | 17.84 |
| G24 | 0.92 | 0.67 | 0.70 | 1.03 | 12.97 | G109 | 0.36 | 0.70 | 0.88 | 1.31 | 4.50 |
| G25 | 1.21 | 0.69 | 0.62 | 0.92 | 17.34 | G110 | 0.98 | 0.79 | 0.70 | 1.04 | 13.77 |
| G26 | 1.02 | 0.60 | 0.65 | 0.97 | 14.47 | G111 | 1.10 | 0.56 | 0.62 | 0.92 | 15.60 |
| G27 | 0.48 | 0.75 | 0.85 | 1.25 | 6.20 | G112 | 0.88 | 0.83 | 0.73 | 1.08 | 12.36 |
| G28 | 0.88 | 0.75 | 0.72 | 1.07 | 12.37 | G113 | 0.65 | 0.82 | 0.80 | 1.18 | 8.83 |
| G29 | 1.16 | 0.60 | 0.62 | 0.91 | 16.56 | G114 | 0.93 | 0.62 | 0.68 | 1.01 | 13.13 |
| G30 | 1.12 | 0.65 | 0.64 | 0.94 | 15.96 | G115 | 0.94 | 0.75 | 0.71 | 1.04 | 13.23 |
| G31 | 0.23 | 0.71 | 0.94 | 1.38 | 2.46 | G116 | 0.72 | 0.58 | 0.75 | 1.10 | 9.83 |
| G32 | 1.42 | 0.88 | 0.61 | 0.90 | 20.40 | G117 | 0.70 | 0.67 | 0.77 | 1.13 | 9.60 |
| G33 | 1.13 | 0.70 | 0.65 | 0.95 | 16.16 | G118 | 1.17 | 0.71 | 0.64 | 0.94 | 16.67 |
| G34 | 0.93 | 0.72 | 0.71 | 1.04 | 13.03 | G119 | 0.87 | 0.78 | 0.73 | 1.08 | 12.13 |
| G35 | 1.35 | 0.60 | 0.57 | 0.84 | 19.47 | G120 | 0.87 | 0.47 | 0.67 | 0.99 | 12.17 |
| G36 | 1.19 | 0.43 | 0.56 | 0.82 | 16.97 | G121 | 0.91 | 0.74 | 0.71 | 1.05 | 12.76 |
| G37 | 1.12 | 0.64 | 0.64 | 0.94 | 15.87 | G122 | 0.58 | 0.71 | 0.81 | 1.20 | 7.77 |
| G38 | 1.05 | 0.45 | 0.61 | 0.90 | 14.83 | G123 | 1.10 | 1.06 | 0.71 | 1.05 | 15.56 |
| G39 | 1.53 | 0.58 | 0.52 | 0.77 | 22.14 | G124 | 1.04 | 0.58 | 0.65 | 0.95 | 14.70 |
| G40 | 2.08 | 0.74 | 0.45 | 0.67 | 30.50 | G125 | 0.72 | 0.75 | 0.77 | 1.14 | 9.90 |
| G41 | 1.04 | 0.54 | 0.63 | 0.94 | 14.74 | G126 | 0.89 | 0.57 | 0.69 | 1.02 | 12.43 |
| G42 | 1.30 | 0.50 | 0.55 | 0.81 | 18.67 | G127 | 1.45 | 0.89 | 0.61 | 0.89 | 20.94 |
| G43 | 0.59 | 0.60 | 0.79 | 1.17 | 7.90 | G128 | 0.62 | 0.60 | 0.78 | 1.15 | 8.37 |
| G44 | 0.99 | 0.59 | 0.66 | 0.98 | 13.90 | G129 | 1.18 | 0.58 | 0.61 | 0.89 | 16.83 |
| G45 | 0.92 | 0.79 | 0.72 | 1.06 | 12.87 | G130 | 0.91 | 1.00 | 0.75 | 1.10 | 12.77 |
| G46 | 0.94 | 0.50 | 0.65 | 0.96 | 13.23 | G131 | 1.04 | 0.75 | 0.68 | 1.00 | 14.80 |
| G47 | 1.57 | 0.79 | 0.56 | 0.83 | 22.80 | G132 | 0.95 | 0.60 | 0.68 | 1.00 | 13.36 |
| G48 | 0.66 | 0.74 | 0.79 | 1.16 | 9.00 | G133 | 1.30 | 0.66 | 0.59 | 0.88 | 18.67 |
| G49 | 1.25 | 0.76 | 0.63 | 0.93 | 17.96 | G134 | 1.26 | 0.75 | 0.62 | 0.92 | 18.00 |
| G50 | 1.02 | 0.67 | 0.67 | 0.99 | 14.43 | G135 | 1.47 | 0.85 | 0.60 | 0.88 | 21.23 |
| G51 | 0.74 | 0.81 | 0.77 | 1.14 | 10.20 | G136 | 0.73 | 0.61 | 0.75 | 1.10 | 9.97 |
| G52 | 1.34 | 0.65 | 0.58 | 0.86 | 19.26 | G137 | 0.98 | 0.81 | 0.70 | 1.04 | 13.84 |
| G53 | 0.72 | 0.50 | 0.73 | 1.07 | 9.96 | G138 | 1.07 | 0.72 | 0.67 | 0.98 | 15.24 |
| G54 | 0.84 | 0.60 | 0.71 | 1.05 | 11.73 | G139 | 0.98 | 0.76 | 0.70 | 1.03 | 13.87 |
| G55 | 1.41 | 0.74 | 0.59 | 0.87 | 20.33 | G140 | 0.90 | 0.64 | 0.70 | 1.03 | 12.57 |
| G56 | 1.44 | 0.59 | 0.54 | 0.80 | 20.80 | G141 | 0.92 | 0.57 | 0.68 | 1.00 | 12.87 |
| G57 | 1.39 | 0.73 | 0.59 | 0.87 | 20.03 | G142 | 0.72 | 0.59 | 0.75 | 1.10 | 9.83 |
| G58 | 1.21 | 0.69 | 0.63 | 0.92 | 17.23 | G143 | 1.35 | 0.90 | 0.63 | 0.93 | 19.34 |
| G59 | 1.46 | 0.51 | 0.51 | 0.76 | 21.04 | G144 | 1.46 | 0.82 | 0.59 | 0.87 | 21.13 |
| G60 | 1.07 | 0.72 | 0.67 | 0.98 | 15.23 | G145 | 1.28 | 0.67 | 0.60 | 0.89 | 18.37 |
| G61 | 1.61 | 0.69 | 0.53 | 0.78 | 23.40 | G146 | 1.64 | 0.58 | 0.49 | 0.73 | 23.87 |
| G62 | 0.48 | 0.68 | 0.84 | 1.24 | 6.26 | G147 | 1.11 | 0.80 | 0.67 | 0.99 | 15.80 |
| G63 | 0.64 | 0.66 | 0.78 | 1.16 | 8.67 | G148 | 0.60 | 0.69 | 0.80 | 1.18 | 8.04 |
| G64 | 1.33 | 0.65 | 0.59 | 0.87 | 19.06 | G149 | 0.39 | 0.82 | 0.89 | 1.31 | 4.83 |
| G65 | 1.14 | 0.72 | 0.65 | 0.96 | 16.27 | G150 | 0.83 | 0.66 | 0.72 | 1.07 | 11.56 |
| G66 | 0.88 | 0.61 | 0.70 | 1.03 | 12.30 | G151 | 1.05 | 0.71 | 0.67 | 0.99 | 14.94 |
| G67 | 0.69 | 0.74 | 0.78 | 1.15 | 9.37 | G152 | 0.73 | 0.83 | 0.78 | 1.15 | 10.10 |
| G68 | 1.10 | 0.48 | 0.60 | 0.89 | 15.56 | G153 | 0.75 | 0.65 | 0.75 | 1.10 | 10.27 |
| G69 | 0.52 | 0.62 | 0.82 | 1.21 | 6.84 | G154 | 1.35 | 0.69 | 0.59 | 0.87 | 19.40 |
| G70 | 1.17 | 0.59 | 0.61 | 0.90 | 16.66 | G155 | 0.76 | 0.66 | 0.75 | 1.10 | 10.43 |
| G71 | 1.46 | 0.76 | 0.58 | 0.85 | 21.14 | G156 | 1.12 | 0.62 | 0.63 | 0.93 | 15.90 |
| G72 | 0.88 | 0.63 | 0.70 | 1.04 | 12.24 | G157 | 0.46 | 0.71 | 0.85 | 1.25 | 6.03 |
| G73 | 0.51 | 0.60 | 0.82 | 1.21 | 6.77 | G158 | 0.46 | 0.77 | 0.86 | 1.27 | 5.90 |
| G74 | 1.00 | 0.63 | 0.67 | 0.98 | 14.13 | G159 | 0.79 | 0.70 | 0.74 | 1.09 | 11.00 |
| G75 | 0.76 | 0.70 | 0.75 | 1.11 | 10.43 | G160 | 0.97 | 0.54 | 0.66 | 0.97 | 13.64 |
| G76 | 1.41 | 0.75 | 0.59 | 0.87 | 20.30 | G161 | 1.41 | 0.70 | 0.58 | 0.85 | 20.30 |
| G77 | 1.20 | 0.83 | 0.65 | 0.96 | 17.10 | G162 | 0.83 | 0.58 | 0.71 | 1.05 | 11.53 |
| G78 | 1.15 | 0.82 | 0.66 | 0.98 | 16.44 | G163 | 0.74 | 0.77 | 0.77 | 1.13 | 10.13 |
| G79 | 1.17 | 0.68 | 0.63 | 0.93 | 16.73 | G164 | 0.43 | 0.79 | 0.87 | 1.28 | 5.43 |
| G80 | 0.43 | 0.74 | 0.86 | 1.28 | 5.47 | G165 | 0.84 | 0.49 | 0.68 | 1.01 | 11.63 |
| G81 | 0.33 | 0.68 | 0.90 | 1.32 | 4.00 | G166 | 0.80 | 0.60 | 0.72 | 1.07 | 11.13 |
| G82 | 1.47 | 0.61 | 0.54 | 0.80 | 21.20 | G167 | 0.59 | 0.72 | 0.81 | 1.19 | 7.90 |
| G83 | 1.11 | 0.53 | 0.61 | 0.91 | 15.77 | G168 | 1.27 | 0.63 | 0.59 | 0.88 | 18.26 |
| G84 | 0.31 | 0.76 | 0.91 | 1.34 | 3.64 | G169 | 1.27 | 0.52 | 0.57 | 0.84 | 18.13 |
| G85 | 0.92 | 0.54 | 0.67 | 0.99 | 12.87 |  |  |  |  |  |  |

SSI, STI, YSI, RSI, and TOL were the abbreviations of stress susceptibility index, stress tolerance index, yield stability index, relative stress index, tolerance index respectively.

| TABLE S7. Calculated drought tolerance and susceptibility indices in 169 wheat accessions based on the relative water content (RWC) indices in 2020-2021. | | | | | | | | | | | |
| --- | --- | --- | --- | --- | --- | --- | --- | --- | --- | --- | --- |
| Code | SSI | STI | YSI | RSI | TOL | Code | SSI | STI | YSI | RSI | TOL |
| G1 | 1.04 | 0.87 | 0.68 | 0.94 | 0.22 | G86 | 0.91 | 0.57 | 0.88 | 1.22 | 0.06 |
| G2 | 0.99 | 0.60 | 0.71 | 0.99 | 0.16 | G87 | 1.09 | 0.73 | 0.59 | 0.82 | 0.28 |
| G3 | 1.04 | 0.69 | 0.65 | 0.90 | 0.22 | G88 | 1.01 | 0.85 | 0.73 | 1.01 | 0.18 |
| G4 | 1.08 | 0.74 | 0.61 | 0.85 | 0.26 | G89 | 0.97 | 0.42 | 0.70 | 0.97 | 0.14 |
| G5 | 0.97 | 0.97 | 0.81 | 1.12 | 0.13 | G90 | 1.09 | 0.52 | 0.53 | 0.74 | 0.28 |
| G6 | 0.94 | 1.03 | 0.85 | 1.18 | 0.10 | G91 | 0.97 | 0.77 | 0.77 | 1.07 | 0.14 |
| G7 | 0.99 | 0.89 | 0.76 | 1.05 | 0.16 | G92 | 0.91 | 0.81 | 0.90 | 1.24 | 0.06 |
| G8 | 0.99 | 0.89 | 0.76 | 1.05 | 0.16 | G93 | 0.91 | 0.78 | 0.89 | 1.24 | 0.06 |
| G9 | 1.00 | 0.47 | 0.67 | 0.92 | 0.17 | G94 | 1.05 | 0.82 | 0.66 | 0.92 | 0.23 |
| G10 | 0.92 | 0.96 | 0.88 | 1.21 | 0.08 | G95 | 0.98 | 0.87 | 0.77 | 1.07 | 0.15 |
| G11 | 1.11 | 0.75 | 0.57 | 0.79 | 0.30 | G96 | 1.06 | 0.72 | 0.63 | 0.87 | 0.24 |
| G12 | 1.19 | 1.31 | 0.58 | 0.80 | 0.39 | G97 | 1.01 | 1.04 | 0.75 | 1.04 | 0.18 |
| G13 | 1.00 | 0.44 | 0.66 | 0.92 | 0.17 | G98 | 0.97 | 0.70 | 0.78 | 1.08 | 0.13 |
| G14 | 1.04 | 0.42 | 0.58 | 0.80 | 0.22 | G99 | 1.06 | 0.58 | 0.60 | 0.83 | 0.24 |
| G15 | 1.08 | 0.77 | 0.62 | 0.86 | 0.26 | G100 | 0.91 | 0.59 | 0.88 | 1.22 | 0.06 |
| G16 | 0.94 | 0.64 | 0.81 | 1.13 | 0.10 | G101 | 0.97 | 1.25 | 0.83 | 1.15 | 0.13 |
| G17 | 0.99 | 0.65 | 0.72 | 1.00 | 0.16 | G102 | 1.09 | 0.62 | 0.56 | 0.78 | 0.28 |
| G18 | 1.09 | 0.73 | 0.59 | 0.82 | 0.28 | G103 | 1.04 | 0.75 | 0.66 | 0.92 | 0.22 |
| G19 | 1.13 | 0.66 | 0.53 | 0.73 | 0.32 | G104 | 0.99 | 0.71 | 0.73 | 1.02 | 0.16 |
| G20 | 1.05 | 0.43 | 0.57 | 0.78 | 0.23 | G105 | 0.94 | 0.51 | 0.80 | 1.10 | 0.10 |
| G21 | 1.00 | 0.84 | 0.74 | 1.02 | 0.17 | G106 | 0.96 | 0.27 | 0.68 | 0.95 | 0.12 |
| G22 | 1.01 | 0.88 | 0.73 | 1.01 | 0.18 | G107 | 1.05 | 0.62 | 0.62 | 0.86 | 0.23 |
| G23 | 1.09 | 0.76 | 0.59 | 0.82 | 0.28 | G108 | 1.05 | 0.73 | 0.65 | 0.90 | 0.23 |
| G24 | 1.15 | 0.71 | 0.52 | 0.72 | 0.34 | G109 | 1.01 | 0.68 | 0.70 | 0.97 | 0.18 |
| G25 | 1.03 | 0.89 | 0.70 | 0.96 | 0.21 | G110 | 1.07 | 0.73 | 0.62 | 0.86 | 0.25 |
| G26 | 1.04 | 0.44 | 0.58 | 0.81 | 0.22 | G111 | 1.05 | 0.65 | 0.63 | 0.87 | 0.23 |
| G27 | 0.96 | 0.89 | 0.81 | 1.13 | 0.12 | G112 | 1.09 | 0.72 | 0.60 | 0.83 | 0.27 |
| G28 | 1.14 | 0.70 | 0.53 | 0.73 | 0.33 | G113 | 0.90 | 0.71 | 0.91 | 1.26 | 0.05 |
| G29 | 0.93 | 0.58 | 0.82 | 1.14 | 0.09 | G114 | 0.92 | 1.10 | 0.88 | 1.22 | 0.08 |
| G30 | 0.99 | 1.26 | 0.79 | 1.10 | 0.16 | G115 | 1.04 | 0.81 | 0.67 | 0.93 | 0.22 |
| G31 | 0.97 | 0.46 | 0.71 | 0.99 | 0.14 | G116 | 1.06 | 0.61 | 0.61 | 0.84 | 0.24 |
| G32 | 0.98 | 0.62 | 0.73 | 1.02 | 0.15 | G117 | 0.91 | 0.70 | 0.89 | 1.23 | 0.06 |
| G33 | 0.98 | 1.00 | 0.78 | 1.08 | 0.15 | G118 | 0.92 | 0.75 | 0.86 | 1.19 | 0.08 |
| G34 | 0.98 | 0.84 | 0.77 | 1.06 | 0.15 | G119 | 1.05 | 0.65 | 0.63 | 0.87 | 0.23 |
| G35 | 1.02 | 0.86 | 0.72 | 0.99 | 0.19 | G120 | 0.96 | 0.64 | 0.78 | 1.08 | 0.12 |
| G36 | 0.91 | 0.63 | 0.87 | 1.20 | 0.07 | G121 | 0.94 | 0.67 | 0.82 | 1.13 | 0.10 |
| G37 | 1.00 | 0.69 | 0.72 | 0.99 | 0.17 | G122 | 1.09 | 0.58 | 0.56 | 0.78 | 0.27 |
| G38 | 1.02 | 0.51 | 0.65 | 0.90 | 0.19 | G123 | 0.95 | 0.62 | 0.80 | 1.10 | 0.11 |
| G39 | 0.87 | 1.03 | 0.97 | 1.34 | 0.02 | G124 | 0.97 | 0.86 | 0.78 | 1.08 | 0.14 |
| G40 | 1.00 | 0.81 | 0.73 | 1.02 | 0.17 | G125 | 0.96 | 0.99 | 0.82 | 1.14 | 0.12 |
| G41 | 0.91 | 1.11 | 0.90 | 1.24 | 0.07 | G126 | 0.95 | 0.76 | 0.81 | 1.13 | 0.11 |
| G42 | 0.99 | 0.71 | 0.73 | 1.02 | 0.16 | G127 | 1.01 | 0.88 | 0.73 | 1.01 | 0.18 |
| G43 | 0.98 | 0.94 | 0.78 | 1.08 | 0.15 | G128 | 1.03 | 0.76 | 0.69 | 0.95 | 0.20 |
| G44 | 0.94 | 0.29 | 0.74 | 1.02 | 0.10 | G129 | 1.02 | 0.93 | 0.72 | 1.00 | 0.19 |
| G45 | 1.13 | 0.71 | 0.54 | 0.75 | 0.32 | G130 | 0.97 | 0.82 | 0.79 | 1.10 | 0.13 |
| G46 | 1.01 | 0.73 | 0.71 | 0.98 | 0.18 | G131 | 0.95 | 0.98 | 0.83 | 1.16 | 0.11 |
| G47 | 0.85 | 0.41 | 1.00 | 1.39 | 0.00 | G132 | 1.15 | 0.84 | 0.54 | 0.75 | 0.35 |
| G48 | 1.08 | 0.80 | 0.62 | 0.86 | 0.26 | G133 | 0.82 | 0.84 | 1.07 | 1.49 | -0.04 |
| G49 | 1.02 | 1.06 | 0.74 | 1.03 | 0.19 | G134 | 0.86 | 1.02 | 0.98 | 1.36 | 0.01 |
| G50 | 1.09 | 0.93 | 0.64 | 0.88 | 0.27 | G135 | 0.94 | 0.54 | 0.80 | 1.11 | 0.10 |
| G51 | 0.88 | 0.77 | 0.95 | 1.31 | 0.03 | G136 | 1.08 | 0.92 | 0.64 | 0.89 | 0.26 |
| G52 | 1.01 | 0.70 | 0.70 | 0.98 | 0.18 | G137 | 0.88 | 0.63 | 0.94 | 1.30 | 0.03 |
| G53 | 1.02 | 0.40 | 0.61 | 0.85 | 0.19 | G138 | 0.92 | 0.45 | 0.82 | 1.14 | 0.08 |
| G54 | 1.01 | 0.88 | 0.73 | 1.01 | 0.18 | G139 | 0.98 | 0.87 | 0.77 | 1.07 | 0.15 |
| G55 | 1.12 | 0.73 | 0.56 | 0.77 | 0.31 | G140 | 0.97 | 0.95 | 0.79 | 1.10 | 0.14 |
| G56 | 0.93 | 1.18 | 0.87 | 1.21 | 0.09 | G141 | 1.07 | 0.76 | 0.63 | 0.87 | 0.25 |
| G57 | 0.98 | 0.30 | 0.64 | 0.89 | 0.15 | G142 | 1.00 | 1.00 | 0.76 | 1.05 | 0.17 |
| G58 | 0.87 | 0.67 | 0.96 | 1.33 | 0.02 | G143 | 0.97 | 0.65 | 0.77 | 1.06 | 0.13 |
| G59 | 1.11 | 0.69 | 0.56 | 0.77 | 0.30 | G144 | 0.97 | 0.88 | 0.80 | 1.10 | 0.13 |
| G60 | 1.05 | 0.85 | 0.67 | 0.92 | 0.23 | G145 | 1.08 | 0.63 | 0.59 | 0.81 | 0.26 |
| G61 | 0.91 | 0.30 | 0.81 | 1.12 | 0.07 | G146 | 1.06 | 0.90 | 0.66 | 0.92 | 0.24 |
| G62 | 1.03 | 1.07 | 0.73 | 1.01 | 0.20 | G147 | 0.93 | 1.26 | 0.88 | 1.22 | 0.09 |
| G63 | 0.90 | 0.86 | 0.92 | 1.27 | 0.05 | G148 | 0.98 | 0.29 | 0.63 | 0.88 | 0.15 |
| G64 | 1.08 | 0.63 | 0.59 | 0.81 | 0.26 | G149 | 1.09 | 0.75 | 0.60 | 0.84 | 0.27 |
| G65 | 0.97 | 0.34 | 0.67 | 0.93 | 0.14 | G150 | 1.03 | 0.88 | 0.71 | 0.98 | 0.20 |
| G66 | 1.01 | 1.01 | 0.75 | 1.03 | 0.18 | G151 | 1.03 | 0.76 | 0.69 | 0.95 | 0.20 |
| G67 | 1.01 | 0.82 | 0.72 | 1.00 | 0.18 | G152 | 1.00 | 0.96 | 0.75 | 1.04 | 0.17 |
| G68 | 0.97 | 0.71 | 0.76 | 1.06 | 0.14 | G153 | 1.00 | 0.36 | 0.63 | 0.87 | 0.17 |
| G69 | 1.03 | 0.79 | 0.69 | 0.96 | 0.20 | G154 | 0.96 | 0.66 | 0.79 | 1.09 | 0.12 |
| G70 | 0.97 | 1.11 | 0.82 | 1.13 | 0.13 | G155 | 1.01 | 0.88 | 0.73 | 1.01 | 0.18 |
| G71 | 1.01 | 0.33 | 0.60 | 0.83 | 0.18 | G156 | 1.00 | 1.00 | 0.76 | 1.05 | 0.17 |
| G72 | 0.97 | 0.95 | 0.79 | 1.10 | 0.14 | G157 | 0.97 | 0.42 | 0.70 | 0.97 | 0.14 |
| G73 | 0.99 | 0.83 | 0.75 | 1.04 | 0.16 | G158 | 1.08 | 0.74 | 0.61 | 0.85 | 0.26 |
| G74 | 0.99 | 0.35 | 0.64 | 0.89 | 0.16 | G159 | 0.99 | 1.08 | 0.78 | 1.08 | 0.16 |
| G75 | 0.93 | 0.71 | 0.84 | 1.16 | 0.09 | G160 | 1.06 | 0.44 | 0.56 | 0.77 | 0.24 |
| G76 | 0.97 | 0.44 | 0.71 | 0.98 | 0.14 | G161 | 0.99 | 0.98 | 0.77 | 1.06 | 0.16 |
| G77 | 0.97 | 1.07 | 0.81 | 1.13 | 0.13 | G162 | 1.00 | 0.69 | 0.72 | 0.99 | 0.17 |
| G78 | 1.08 | 0.47 | 0.54 | 0.75 | 0.26 | G163 | 1.04 | 0.75 | 0.66 | 0.92 | 0.22 |
| G79 | 0.96 | 0.34 | 0.71 | 0.99 | 0.12 | G164 | 1.06 | 0.41 | 0.55 | 0.76 | 0.24 |
| G80 | 1.05 | 0.34 | 0.53 | 0.74 | 0.23 | G165 | 0.88 | 1.05 | 0.95 | 1.32 | 0.03 |
| G81 | 1.00 | 0.75 | 0.73 | 1.01 | 0.17 | G166 | 0.97 | 0.89 | 0.78 | 1.09 | 0.14 |
| G82 | 0.97 | 0.69 | 0.76 | 1.05 | 0.14 | G167 | 0.89 | 0.52 | 0.91 | 1.27 | 0.04 |
| G83 | 0.92 | 0.52 | 0.83 | 1.16 | 0.08 | G168 | 1.10 | 0.80 | 0.59 | 0.82 | 0.29 |
| G84 | 0.95 | 0.98 | 0.83 | 1.16 | 0.11 | G169 | 0.97 | 1.01 | 0.81 | 1.12 | 0.13 |
| G85 | 1.00 | 0.75 | 0.73 | 1.01 | 0.17 |  |  |  |  |  |  |

SSI, STI, YSI, RSI, and TOL were the abbreviations of stress susceptibility index, stress tolerance index, yield stability index, relative stress index, tolerance index respectively.

| TABLE S8. Calculated drought tolerance and susceptibility indices in 169 wheat accessions based on the plant height (PH) indices in 2020-2021. | | | | | | | | | | | |
| --- | --- | --- | --- | --- | --- | --- | --- | --- | --- | --- | --- |
| Code | SSI | STI | YSI | RSI | TOL | Code | SSI | STI | YSI | RSI | TOL |
| G1 | 0.57 | 0.91 | 0.94 | 1.06 | 5.53 | G86 | 0.42 | 0.58 | 0.95 | 1.06 | 3.87 |
| G2 | 1.11 | 1.15 | 0.89 | 1.00 | 11.73 | G87 | 1.28 | 0.84 | 0.86 | 0.96 | 13.80 |
| G3 | 0.30 | 0.64 | 0.97 | 1.09 | 2.46 | G88 | 0.62 | 0.84 | 0.93 | 1.05 | 6.13 |
| G4 | 1.57 | 1.14 | 0.85 | 0.95 | 17.13 | G89 | 0.14 | 0.62 | 0.99 | 1.11 | 0.66 |
| G5 | 1.40 | 1.04 | 0.86 | 0.96 | 15.13 | G90 | 1.49 | 0.80 | 0.83 | 0.93 | 16.13 |
| G6 | 1.70 | 0.68 | 0.79 | 0.89 | 18.60 | G91 | 0.49 | 0.75 | 0.95 | 1.06 | 4.60 |
| G7 | 1.56 | 0.91 | 0.83 | 0.93 | 17.00 | G92 | 0.88 | 0.68 | 0.89 | 1.00 | 9.13 |
| G8 | 0.44 | 1.00 | 0.96 | 1.08 | 4.07 | G93 | 0.38 | 0.78 | 0.96 | 1.08 | 3.33 |
| G9 | 0.80 | 1.32 | 0.93 | 1.04 | 8.20 | G94 | 1.16 | 0.92 | 0.88 | 0.98 | 12.33 |
| G10 | 0.18 | 0.43 | 0.98 | 1.10 | 1.07 | G95 | 0.76 | 0.92 | 0.92 | 1.03 | 7.80 |
| G11 | 0.57 | 1.20 | 0.95 | 1.06 | 5.60 | G96 | 1.08 | 1.04 | 0.89 | 1.00 | 11.46 |
| G12 | 0.76 | 0.57 | 0.90 | 1.01 | 7.73 | G97 | 0.73 | 1.20 | 0.93 | 1.05 | 7.46 |
| G13 | 0.97 | 0.93 | 0.90 | 1.01 | 10.13 | G98 | 0.13 | 0.54 | 0.99 | 1.11 | 0.47 |
| G14 | 2.03 | 0.97 | 0.79 | 0.89 | 22.33 | G99 | 1.09 | 1.22 | 0.90 | 1.01 | 11.60 |
| G15 | 1.16 | 1.08 | 0.88 | 0.99 | 12.33 | G100 | 0.54 | 1.05 | 0.95 | 1.06 | 5.26 |
| G16 | 1.22 | 1.02 | 0.88 | 0.98 | 13.00 | G101 | 0.41 | 1.30 | 0.97 | 1.08 | 3.74 |
| G17 | 0.30 | 0.52 | 0.97 | 1.08 | 2.47 | G102 | 0.66 | 0.83 | 0.93 | 1.04 | 6.66 |
| G18 | 0.30 | 0.83 | 0.97 | 1.09 | 2.40 | G103 | 0.98 | 1.10 | 0.90 | 1.01 | 10.33 |
| G19 | 0.83 | 0.84 | 0.91 | 1.02 | 8.53 | G104 | 1.03 | 1.17 | 0.90 | 1.01 | 10.87 |
| G20 | 0.58 | 0.91 | 0.94 | 1.05 | 5.73 | G105 | 0.12 | 0.74 | 1.00 | 1.12 | 0.34 |
| G21 | 2.00 | 1.29 | 0.82 | 0.92 | 22.00 | G106 | 0.73 | 0.87 | 0.92 | 1.03 | 7.40 |
| G22 | 2.36 | 1.23 | 0.78 | 0.88 | 26.20 | G107 | 0.50 | 0.81 | 0.95 | 1.06 | 4.73 |
| G23 | 1.50 | 0.97 | 0.84 | 0.95 | 16.26 | G108 | 1.09 | 1.03 | 0.89 | 1.00 | 11.60 |
| G24 | 0.40 | 0.96 | 0.96 | 1.08 | 3.60 | G109 | 0.98 | 1.05 | 0.90 | 1.01 | 10.34 |
| G25 | 0.33 | 1.25 | 0.97 | 1.09 | 2.80 | G110 | 0.79 | 1.08 | 0.92 | 1.04 | 8.07 |
| G26 | 0.94 | 0.90 | 0.90 | 1.01 | 9.87 | G111 | 0.66 | 0.68 | 0.92 | 1.03 | 6.66 |
| G27 | 0.89 | 0.83 | 0.90 | 1.01 | 9.20 | G112 | 0.92 | 0.62 | 0.88 | 0.99 | 9.60 |
| G28 | 0.80 | 1.07 | 0.92 | 1.03 | 8.20 | G113 | 0.58 | 0.53 | 0.92 | 1.03 | 5.73 |
| G29 | 1.55 | 1.10 | 0.85 | 0.95 | 16.80 | G114 | 0.29 | 0.80 | 0.97 | 1.09 | 2.34 |
| G30 | 1.05 | 1.06 | 0.89 | 1.00 | 11.14 | G115 | 0.96 | 0.83 | 0.89 | 1.00 | 10.07 |
| G31 | 1.00 | 1.04 | 0.90 | 1.01 | 10.53 | G116 | 0.72 | 1.30 | 0.94 | 1.05 | 7.27 |
| G32 | 1.17 | 1.22 | 0.89 | 1.00 | 12.53 | G117 | 0.67 | 0.68 | 0.92 | 1.03 | 6.73 |
| G33 | 1.14 | 1.33 | 0.90 | 1.01 | 12.13 | G118 | 0.79 | 0.70 | 0.91 | 1.02 | 8.07 |
| G34 | 0.37 | 0.78 | 0.96 | 1.08 | 3.27 | G119 | 0.83 | 1.24 | 0.92 | 1.04 | 8.60 |
| G35 | 0.50 | 0.83 | 0.95 | 1.06 | 4.80 | G120 | 0.24 | 0.48 | 0.97 | 1.09 | 1.80 |
| G36 | 1.06 | 0.74 | 0.87 | 0.98 | 11.26 | G121 | 0.84 | 1.01 | 0.91 | 1.03 | 8.66 |
| G37 | 1.02 | 0.75 | 0.88 | 0.99 | 10.73 | G122 | 1.13 | 1.06 | 0.89 | 0.99 | 12.06 |
| G38 | 0.77 | 0.90 | 0.92 | 1.03 | 7.86 | G123 | 0.24 | 0.64 | 0.98 | 1.10 | 1.80 |
| G39 | 1.12 | 1.07 | 0.89 | 1.00 | 11.87 | G124 | 0.99 | 1.23 | 0.91 | 1.02 | 10.40 |
| G40 | 0.80 | 1.02 | 0.92 | 1.03 | 8.20 | G125 | 0.76 | 0.82 | 0.92 | 1.03 | 7.74 |
| G41 | 1.38 | 0.90 | 0.85 | 0.95 | 14.93 | G126 | 1.09 | 1.33 | 0.90 | 1.01 | 11.54 |
| G42 | 1.37 | 1.03 | 0.86 | 0.97 | 14.73 | G127 | 0.58 | 0.57 | 0.93 | 1.04 | 5.67 |
| G43 | 30.03 | 5.07 | 0.23 | 0.26 | 344.93 | G128 | 0.40 | 0.87 | 0.96 | 1.08 | 3.60 |
| G44 | 0.70 | 0.82 | 0.92 | 1.03 | 7.07 | G129 | 0.56 | 0.56 | 0.93 | 1.04 | 5.40 |
| G45 | 0.95 | 0.89 | 0.90 | 1.01 | 9.94 | G130 | 0.50 | 0.50 | 0.93 | 1.05 | 4.80 |
| G46 | 0.77 | 1.07 | 0.92 | 1.04 | 7.86 | G131 | 0.78 | 0.89 | 0.92 | 1.03 | 7.93 |
| G47 | 0.65 | 0.71 | 0.92 | 1.04 | 6.53 | G132 | 0.99 | 0.71 | 0.88 | 0.99 | 10.40 |
| G48 | 1.42 | 1.40 | 0.87 | 0.98 | 15.40 | G133 | 0.13 | 0.66 | 0.99 | 1.12 | 0.46 |
| G49 | 1.45 | 1.19 | 0.86 | 0.97 | 15.73 | G134 | 0.32 | 0.96 | 0.97 | 1.09 | 2.67 |
| G50 | 1.38 | 1.31 | 0.87 | 0.98 | 14.87 | G135 | 0.98 | 0.55 | 0.87 | 0.97 | 10.26 |
| G51 | 0.47 | 0.58 | 0.94 | 1.06 | 4.47 | G136 | 0.36 | 0.84 | 0.97 | 1.08 | 3.13 |
| G52 | 0.84 | 0.86 | 0.91 | 1.02 | 8.67 | G137 | 1.02 | 0.55 | 0.86 | 0.97 | 10.73 |
| G53 | 0.63 | 0.65 | 0.92 | 1.04 | 6.27 | G138 | 0.82 | 0.53 | 0.89 | 1.00 | 8.46 |
| G54 | 1.33 | 1.08 | 0.87 | 0.97 | 14.27 | G139 | 1.05 | 1.05 | 0.89 | 1.00 | 11.14 |
| G55 | 0.80 | 1.27 | 0.93 | 1.04 | 8.27 | G140 | 1.21 | 1.11 | 0.88 | 0.99 | 12.93 |
| G56 | 0.86 | 1.07 | 0.91 | 1.03 | 8.93 | G141 | 0.86 | 1.38 | 0.92 | 1.04 | 8.93 |
| G57 | 0.94 | 0.58 | 0.87 | 0.98 | 9.87 | G142 | 0.60 | 0.53 | 0.92 | 1.03 | 5.86 |
| G58 | 1.83 | 1.00 | 0.81 | 0.91 | 20.07 | G143 | 0.27 | 0.73 | 0.98 | 1.09 | 2.07 |
| G59 | 1.79 | 0.73 | 0.79 | 0.89 | 19.67 | G144 | 1.34 | 0.91 | 0.86 | 0.96 | 14.40 |
| G60 | 0.66 | 1.09 | 0.94 | 1.05 | 6.60 | G145 | 0.71 | 1.02 | 0.93 | 1.04 | 7.20 |
| G61 | 0.05 | 0.64 | 1.01 | 1.13 | -0.40 | G146 | 0.50 | 0.67 | 0.94 | 1.06 | 4.80 |
| G62 | 1.36 | 0.68 | 0.83 | 0.93 | 14.67 | G147 | 0.37 | 0.81 | 0.96 | 1.08 | 3.26 |
| G63 | 1.20 | 0.77 | 0.86 | 0.96 | 12.87 | G148 | 0.47 | 0.87 | 0.95 | 1.07 | 4.47 |
| G64 | 1.36 | 1.14 | 0.87 | 0.97 | 14.67 | G149 | 1.02 | 1.16 | 0.90 | 1.01 | 10.73 |
| G65 | 0.14 | 0.80 | 0.99 | 1.11 | 0.66 | G150 | 0.58 | 1.11 | 0.95 | 1.06 | 5.70 |
| G66 | 0.40 | 0.76 | 0.96 | 1.08 | 3.60 | G151 | 0.51 | 0.69 | 0.94 | 1.06 | 4.86 |
| G67 | 0.60 | 0.38 | 0.91 | 1.02 | 5.87 | G152 | 0.40 | 0.65 | 0.95 | 1.07 | 3.60 |
| G68 | 1.08 | 0.51 | 0.85 | 0.95 | 11.47 | G153 | 1.20 | 1.11 | 0.88 | 0.99 | 12.80 |
| G69 | 1.33 | 1.29 | 0.88 | 0.98 | 14.33 | G154 | 0.54 | 0.60 | 0.93 | 1.05 | 5.20 |
| G70 | 1.19 | 1.08 | 0.88 | 0.99 | 12.73 | G155 | 0.66 | 0.91 | 0.93 | 1.04 | 6.66 |
| G71 | 0.73 | 0.44 | 0.89 | 1.00 | 7.40 | G156 | 0.79 | 0.78 | 0.91 | 1.02 | 8.14 |
| G72 | 0.32 | 1.23 | 0.98 | 1.09 | 2.67 | G157 | 0.74 | 0.72 | 0.91 | 1.02 | 7.53 |
| G73 | 0.68 | 0.98 | 0.93 | 1.04 | 6.86 | G158 | 1.31 | 0.68 | 0.84 | 0.94 | 14.07 |
| G74 | 0.59 | 0.82 | 0.94 | 1.05 | 5.74 | G159 | 0.72 | 0.62 | 0.91 | 1.02 | 7.27 |
| G75 | 0.31 | 0.69 | 0.97 | 1.09 | 2.53 | G160 | 0.71 | 0.84 | 0.92 | 1.04 | 7.13 |
| G76 | 0.30 | 0.50 | 0.97 | 1.08 | 2.40 | G161 | 0.57 | 0.89 | 0.94 | 1.06 | 5.60 |
| G77 | 1.49 | 0.99 | 0.85 | 0.95 | 16.14 | G162 | 0.57 | 1.00 | 0.94 | 1.06 | 5.53 |
| G78 | 0.75 | 0.50 | 0.89 | 1.00 | 7.60 | G163 | 0.50 | 0.70 | 0.94 | 1.06 | 4.80 |
| G79 | 0.54 | 0.73 | 0.94 | 1.05 | 5.27 | G164 | 0.67 | 0.68 | 0.92 | 1.03 | 6.67 |
| G80 | 0.95 | 0.88 | 0.90 | 1.01 | 9.93 | G165 | 1.29 | 0.92 | 0.86 | 0.97 | 13.87 |
| G81 | 0.88 | 0.89 | 0.90 | 1.02 | 9.14 | G166 | 0.64 | 0.70 | 0.92 | 1.04 | 6.40 |
| G82 | 0.47 | 0.97 | 0.95 | 1.07 | 4.40 | G167 | 0.50 | 1.06 | 0.95 | 1.07 | 4.74 |
| G83 | 0.55 | 0.83 | 0.94 | 1.06 | 5.33 | G168 | 1.06 | 1.13 | 0.90 | 1.01 | 11.20 |
| G84 | 0.79 | 0.86 | 0.91 | 1.03 | 8.07 | G169 | 1.22 | 1.28 | 0.89 | 1.00 | 13.00 |
| G85 | 0.31 | 0.62 | 0.97 | 1.09 | 2.53 |  |  |  |  |  |  |

SSI, STI, YSI, RSI, and TOL were the abbreviations of stress susceptibility index, stress tolerance index, yield stability index, relative stress index, tolerance index respectively.

| TABLE S9. Calculated drought tolerance and susceptibility indices in 169 wheat accessions based on the thousand grain weight (TGW) indices in 2020-2021. | | | | | | | | | | | |
| --- | --- | --- | --- | --- | --- | --- | --- | --- | --- | --- | --- |
| Code | SSI | STI | YSI | RSI | TOL | Code | SSI | STI | YSI | RSI | TOL |
| G1 | 1.03 | 0.89 | 0.82 | 1.00 | 6.13 | G86 | 0.05 | 1.05 | 0.76 | 0.93 | 9.06 |
| G2 | 0.02 | 0.75 | 0.86 | 1.05 | 4.40 | G87 | 0.00 | 0.71 | 1.00 | 1.23 | -0.13 |
| G3 | 0.03 | 0.64 | 0.80 | 0.97 | 5.94 | G88 | 0.03 | 0.65 | 0.79 | 0.96 | 6.36 |
| G4 | 0.02 | 0.62 | 0.85 | 1.04 | 4.19 | G89 | 0.02 | 0.72 | 0.90 | 1.10 | 2.96 |
| G5 | 0.04 | 0.81 | 0.76 | 0.93 | 8.07 | G90 | 0.02 | 0.95 | 0.89 | 1.08 | 3.81 |
| G6 | 0.02 | 1.01 | 0.88 | 1.08 | 4.13 | G91 | 0.04 | 0.60 | 0.77 | 0.94 | 6.70 |
| G7 | 0.02 | 0.88 | 0.89 | 1.09 | 3.56 | G92 | 0.03 | 0.97 | 0.85 | 1.04 | 5.23 |
| G8 | 0.04 | 1.01 | 0.81 | 0.99 | 6.81 | G93 | 0.05 | 0.78 | 0.70 | 0.86 | 10.36 |
| G9 | 0.03 | 1.01 | 0.86 | 1.05 | 4.91 | G94 | 0.04 | 0.73 | 0.76 | 0.92 | 7.79 |
| G10 | 0.05 | 0.83 | 0.73 | 0.89 | 9.44 | G95 | 0.04 | 0.77 | 0.75 | 0.91 | 8.38 |
| G11 | 0.04 | 0.99 | 0.79 | 0.97 | 7.61 | G96 | 0.03 | 0.81 | 0.85 | 1.03 | 4.98 |
| G12 | 0.05 | 0.84 | 0.73 | 0.90 | 9.35 | G97 | 0.03 | 0.98 | 0.87 | 1.06 | 4.64 |
| G13 | 0.04 | 0.83 | 0.76 | 0.93 | 8.25 | G98 | 0.04 | 1.17 | 0.80 | 0.97 | 8.04 |
| G14 | 0.03 | 0.77 | 0.82 | 1.00 | 5.58 | G99 | 0.03 | 0.91 | 0.82 | 1.00 | 6.33 |
| G15 | 0.02 | 0.88 | 0.92 | 1.13 | 2.44 | G100 | 0.04 | 0.90 | 0.78 | 0.95 | 7.74 |
| G16 | 0.03 | 0.79 | 0.85 | 1.03 | 4.87 | G101 | 0.05 | 0.87 | 0.74 | 0.90 | 9.18 |
| G17 | 0.06 | 0.73 | 0.66 | 0.81 | 11.60 | G102 | 0.04 | 1.20 | 0.82 | 1.00 | 7.28 |
| G18 | 0.05 | 0.80 | 0.69 | 0.84 | 11.01 | G103 | 0.03 | 0.96 | 0.81 | 0.99 | 6.61 |
| G19 | 0.04 | 0.91 | 0.79 | 0.96 | 7.57 | G104 | 0.01 | 0.70 | 0.93 | 1.14 | 1.86 |
| G20 | 0.02 | 0.58 | 0.84 | 1.02 | 4.47 | G105 | 0.01 | 0.64 | 0.92 | 1.12 | 2.16 |
| G21 | 0.04 | 1.09 | 0.79 | 0.97 | 7.98 | G106 | 0.02 | 0.63 | 0.91 | 1.11 | 2.50 |
| G22 | 0.01 | 0.78 | 0.95 | 1.16 | 1.40 | G107 | 0.02 | 0.85 | 0.87 | 1.06 | 4.28 |
| G23 | 0.04 | 0.94 | 0.78 | 0.95 | 8.10 | G108 | 0.04 | 0.76 | 0.76 | 0.93 | 7.88 |
| G24 | 0.03 | 0.77 | 0.82 | 1.00 | 5.63 | G109 | 0.03 | 0.69 | 0.81 | 0.99 | 5.81 |
| G25 | 0.03 | 0.86 | 0.84 | 1.02 | 5.40 | G110 | 0.03 | 0.68 | 0.80 | 0.98 | 5.97 |
| G26 | 0.05 | 0.61 | 0.66 | 0.80 | 10.84 | G111 | 0.03 | 0.75 | 0.81 | 0.98 | 6.15 |
| G27 | 0.04 | 0.70 | 0.78 | 0.95 | 7.01 | G112 | 0.03 | 0.87 | 0.85 | 1.04 | 4.99 |
| G28 | 0.04 | 0.91 | 0.78 | 0.95 | 7.97 | G113 | 0.03 | 0.85 | 0.85 | 1.04 | 4.96 |
| G29 | 0.02 | 0.88 | 0.93 | 1.13 | 2.37 | G114 | 0.03 | 0.79 | 0.84 | 1.03 | 4.98 |
| G30 | 0.03 | 0.75 | 0.82 | 0.99 | 5.80 | G115 | 0.03 | 0.64 | 0.79 | 0.97 | 6.10 |
| G31 | 0.02 | 0.77 | 0.86 | 1.05 | 4.29 | G116 | 0.02 | 0.79 | 0.89 | 1.09 | 3.24 |
| G32 | 0.03 | 0.74 | 0.79 | 0.97 | 6.50 | G117 | 0.04 | 0.71 | 0.74 | 0.90 | 8.47 |
| G33 | 0.02 | 0.80 | 0.88 | 1.08 | 3.67 | G118 | 0.02 | 0.96 | 0.89 | 1.08 | 3.84 |
| G34 | 0.04 | 0.83 | 0.79 | 0.97 | 6.92 | G119 | 0.03 | 0.84 | 0.84 | 1.03 | 5.21 |
| G35 | 0.03 | 0.89 | 0.86 | 1.05 | 4.66 | G120 | 0.03 | 0.67 | 0.79 | 0.96 | 6.38 |
| G36 | 0.04 | 1.02 | 0.80 | 0.97 | 7.44 | G121 | 0.03 | 0.70 | 0.82 | 1.00 | 5.43 |
| G37 | 0.04 | 0.83 | 0.78 | 0.95 | 7.56 | G122 | 0.02 | 0.69 | 0.91 | 1.11 | 2.65 |
| G38 | 0.01 | 1.07 | 0.96 | 1.17 | 1.40 | G123 | 0.03 | 0.91 | 0.83 | 1.01 | 5.97 |
| G39 | 0.04 | 0.80 | 0.76 | 0.92 | 8.22 | G124 | 0.04 | 1.06 | 0.80 | 0.98 | 7.42 |
| G40 | 0.01 | 0.83 | 0.93 | 1.14 | 2.11 | G125 | 0.04 | 0.75 | 0.75 | 0.91 | 8.20 |
| G41 | 0.02 | 0.95 | 0.90 | 1.09 | 3.47 | G126 | 0.03 | 1.00 | 0.87 | 1.06 | 4.68 |
| G42 | 0.04 | 0.98 | 0.76 | 0.93 | 8.82 | G127 | 0.03 | 0.82 | 0.85 | 1.03 | 4.92 |
| G43 | 0.05 | 0.87 | 0.71 | 0.86 | 10.72 | G128 | 0.04 | 0.80 | 0.75 | 0.92 | 8.30 |
| G44 | 0.03 | 0.69 | 0.82 | 0.99 | 5.57 | G129 | 0.04 | 0.93 | 0.81 | 0.98 | 6.84 |
| G45 | 0.02 | 0.57 | 0.86 | 1.05 | 3.79 | G130 | 0.03 | 0.96 | 0.86 | 1.05 | 4.71 |
| G46 | 0.02 | 1.01 | 0.91 | 1.11 | 3.09 | G131 | 0.04 | 1.15 | 0.83 | 1.01 | 6.71 |
| G47 | 0.02 | 0.82 | 0.87 | 1.06 | 4.17 | G132 | 0.04 | 0.75 | 0.78 | 0.95 | 7.04 |
| G48 | 0.02 | 0.81 | 0.87 | 1.06 | 4.08 | G133 | 0.04 | 0.66 | 0.75 | 0.91 | 7.71 |
| G49 | 0.03 | 0.80 | 0.83 | 1.01 | 5.54 | G134 | 0.05 | 0.87 | 0.74 | 0.90 | 9.41 |
| G50 | 0.03 | 0.78 | 0.81 | 0.98 | 6.24 | G135 | 0.03 | 0.79 | 0.85 | 1.04 | 4.60 |
| G51 | 0.03 | 0.89 | 0.81 | 0.99 | 6.38 | G136 | 0.02 | 0.73 | 0.86 | 1.05 | 4.16 |
| G52 | 0.03 | 0.77 | 0.79 | 0.97 | 6.64 | G137 | 0.03 | 0.93 | 0.86 | 1.05 | 4.86 |
| G53 | 0.01 | 0.88 | 0.94 | 1.15 | 1.87 | G138 | 0.02 | 0.72 | 0.90 | 1.10 | 2.95 |
| G54 | 0.02 | 0.76 | 0.90 | 1.10 | 2.97 | G139 | 0.04 | 1.03 | 0.80 | 0.97 | 7.56 |
| G55 | 0.02 | 0.74 | 0.85 | 1.04 | 4.43 | G140 | 0.04 | 0.89 | 0.78 | 0.95 | 7.75 |
| G56 | 0.04 | 1.03 | 0.80 | 0.98 | 7.46 | G141 | 0.04 | 0.97 | 0.80 | 0.98 | 7.21 |
| G57 | 0.04 | 0.75 | 0.78 | 0.95 | 7.03 | G142 | 0.05 | 0.72 | 0.70 | 0.86 | 9.92 |
| G58 | 0.04 | 0.84 | 0.80 | 0.97 | 6.81 | G143 | 0.04 | 0.88 | 0.80 | 0.97 | 6.90 |
| G59 | 0.03 | 0.71 | 0.84 | 1.02 | 4.89 | G144 | 0.01 | 0.75 | 0.99 | 1.21 | 0.33 |
| G60 | 0.02 | 0.74 | 0.92 | 1.12 | 2.48 | G145 | 0.03 | 0.73 | 0.79 | 0.96 | 6.66 |
| G61 | 0.02 | 0.65 | 0.90 | 1.09 | 2.93 | G146 | 0.06 | 0.97 | 0.68 | 0.83 | 12.71 |
| G62 | 0.02 | 0.87 | 0.87 | 1.06 | 4.27 | G147 | 0.05 | 0.83 | 0.74 | 0.90 | 9.12 |
| G63 | 0.03 | 0.67 | 0.84 | 1.03 | 4.65 | G148 | 0.03 | 0.79 | 0.80 | 0.98 | 6.53 |
| G64 | 0.02 | 0.76 | 0.89 | 1.08 | 3.47 | G149 | 0.03 | 0.81 | 0.84 | 1.03 | 5.11 |
| G65 | 0.03 | 0.60 | 0.81 | 0.98 | 5.53 | G150 | 0.03 | 0.80 | 0.85 | 1.03 | 4.92 |
| G66 | 0.02 | 0.85 | 0.88 | 1.08 | 3.81 | G151 | 0.03 | 0.77 | 0.83 | 1.01 | 5.37 |
| G67 | 0.02 | 0.85 | 0.87 | 1.06 | 4.22 | G152 | 0.05 | 0.78 | 0.72 | 0.88 | 9.39 |
| G68 | 0.03 | 0.74 | 0.82 | 0.99 | 5.76 | G153 | 0.04 | 0.83 | 0.75 | 0.91 | 8.78 |
| G69 | 0.02 | 0.84 | 0.89 | 1.09 | 3.45 | G154 | 0.02 | 0.81 | 0.88 | 1.07 | 3.84 |
| G70 | 0.04 | 0.92 | 0.81 | 0.98 | 6.75 | G155 | 0.05 | 0.79 | 0.70 | 0.85 | 10.52 |
| G71 | 0.04 | 0.74 | 0.74 | 0.90 | 8.72 | G156 | 0.04 | 0.90 | 0.78 | 0.95 | 7.80 |
| G72 | 0.04 | 0.84 | 0.79 | 0.97 | 7.06 | G157 | 0.04 | 0.72 | 0.77 | 0.94 | 7.28 |
| G73 | 0.04 | 0.81 | 0.80 | 0.97 | 6.76 | G158 | 0.02 | 1.00 | 0.92 | 1.12 | 2.70 |
| G74 | 0.02 | 0.79 | 0.89 | 1.08 | 3.56 | G159 | 0.03 | 0.68 | 0.78 | 0.96 | 6.61 |
| G75 | 0.02 | 0.80 | 0.89 | 1.09 | 3.40 | G160 | 0.02 | 0.70 | 0.91 | 1.11 | 2.47 |
| G76 | 0.02 | 0.77 | 0.88 | 1.08 | 3.61 | G161 | 0.03 | 0.86 | 0.81 | 0.99 | 6.44 |
| G77 | 0.03 | 0.73 | 0.84 | 1.03 | 4.80 | G162 | 0.02 | 0.94 | 0.91 | 1.11 | 3.13 |
| G78 | 0.02 | 0.67 | 0.90 | 1.10 | 2.83 | G163 | 0.06 | 1.19 | 0.71 | 0.86 | 12.47 |
| G79 | 0.03 | 0.91 | 0.84 | 1.03 | 5.34 | G164 | 0.04 | 1.00 | 0.81 | 0.99 | 6.91 |
| G80 | 0.03 | 0.81 | 0.82 | 1.00 | 5.99 | G165 | 0.04 | 0.74 | 0.78 | 0.95 | 7.13 |
| G81 | 0.02 | 0.62 | 0.87 | 1.06 | 3.61 | G166 | 0.03 | 0.99 | 0.86 | 1.05 | 4.79 |
| G82 | 0.03 | 0.62 | 0.78 | 0.95 | 6.44 | G167 | 0.02 | 0.63 | 0.84 | 1.03 | 4.48 |
| G83 | 0.03 | 0.81 | 0.81 | 0.99 | 6.12 | G168 | 0.03 | 0.76 | 0.83 | 1.01 | 5.45 |
| G84 | 0.05 | 0.91 | 0.74 | 0.90 | 9.45 | G169 | 0.05 | 0.88 | 0.74 | 0.91 | 9.23 |
| G85 | 0.04 | 0.84 | 0.79 | 0.96 | 7.09 |  |  |  |  |  |  |

SSI, STI, YSI, RSI, and TOL were the abbreviations of stress susceptibility index, stress tolerance index, yield stability index, relative stress index, tolerance index respectively.

| TABLE S10. Calculated drought tolerance and susceptibility indices in 169 wheat accessions based on the harvest index (HI) indices in 2020-2021. | | | | | | | | | | | |
| --- | --- | --- | --- | --- | --- | --- | --- | --- | --- | --- | --- |
| Code | SSI | STI | YSI | RSI | TOL | Code | SSI | STI | YSI | RSI | TOL |
| G1 | 1.81 | 0.75 | 0.86 | 0.91 | 3.73 | G86 | 0.92 | 0.89 | 0.95 | 1.01 | 1.40 |
| G2 | 1.00 | 0.68 | 0.93 | 0.99 | 1.62 | G87 | 1.85 | 0.92 | 0.87 | 0.92 | 3.84 |
| G3 | 2.19 | 0.84 | 0.83 | 0.88 | 4.74 | G88 | 1.40 | 1.30 | 0.92 | 0.98 | 2.67 |
| G4 | -0.15 | 0.96 | 1.05 | 1.12 | -1.38 | G89 | 1.23 | 0.75 | 0.91 | 0.97 | 2.21 |
| G5 | 1.92 | 1.07 | 0.87 | 0.92 | 4.04 | G90 | 0.51 | 0.81 | 0.99 | 1.05 | 0.34 |
| G6 | 0.03 | 0.94 | 1.03 | 1.10 | -0.93 | G91 | 2.37 | 0.85 | 0.82 | 0.87 | 5.22 |
| G7 | 2.73 | 1.20 | 0.82 | 0.87 | 6.15 | G92 | 1.74 | 1.08 | 0.89 | 0.94 | 3.55 |
| G8 | 0.56 | 0.85 | 0.98 | 1.04 | 0.48 | G93 | 2.06 | 0.85 | 0.84 | 0.90 | 4.39 |
| G9 | 1.14 | 0.98 | 0.93 | 0.99 | 1.98 | G94 | 3.02 | 1.07 | 0.79 | 0.84 | 6.91 |
| G10 | -0.32 | 1.16 | 1.06 | 1.13 | -1.83 | G95 | 1.02 | 0.79 | 0.94 | 0.99 | 1.68 |
| G11 | 1.39 | 0.90 | 0.91 | 0.96 | 2.64 | G96 | 2.10 | 0.90 | 0.85 | 0.90 | 4.49 |
| G12 | 2.07 | 1.16 | 0.86 | 0.92 | 4.43 | G97 | 2.68 | 1.08 | 0.81 | 0.86 | 6.03 |
| G13 | -1.33 | 0.79 | 1.20 | 1.27 | -4.48 | G98 | -0.47 | 0.78 | 1.09 | 1.16 | -2.24 |
| G14 | -0.89 | 0.89 | 1.13 | 1.20 | -3.34 | G99 | 1.45 | 0.76 | 0.89 | 0.95 | 2.81 |
| G15 | 1.66 | 0.87 | 0.88 | 0.93 | 3.36 | G100 | 1.38 | 0.82 | 0.90 | 0.96 | 2.62 |
| G16 | 1.06 | 0.66 | 0.92 | 0.98 | 1.79 | G101 | -0.34 | 0.98 | 1.07 | 1.14 | -1.89 |
| G17 | 1.27 | 1.07 | 0.92 | 0.98 | 2.34 | G102 | 0.44 | 0.72 | 0.99 | 1.05 | 0.15 |
| G18 | 1.56 | 0.98 | 0.90 | 0.95 | 3.09 | G103 | -0.30 | 0.70 | 1.08 | 1.14 | -1.79 |
| G19 | 0.01 | 0.75 | 1.04 | 1.10 | -0.97 | G104 | 1.59 | 0.94 | 0.89 | 0.95 | 3.16 |
| G20 | 1.58 | 0.96 | 0.89 | 0.95 | 3.13 | G105 | 1.69 | 0.88 | 0.88 | 0.93 | 3.43 |
| G21 | 0.16 | 1.13 | 1.02 | 1.08 | -0.58 | G106 | 1.22 | 0.79 | 0.92 | 0.97 | 2.20 |
| G22 | 0.42 | 0.83 | 1.00 | 1.06 | 0.10 | G107 | 1.27 | 0.79 | 0.91 | 0.97 | 2.33 |
| G23 | 2.58 | 1.23 | 0.83 | 0.88 | 5.77 | G108 | 0.71 | 0.91 | 0.97 | 1.03 | 0.85 |
| G24 | 0.37 | 1.01 | 1.00 | 1.06 | -0.03 | G109 | 1.23 | 0.96 | 0.92 | 0.98 | 2.23 |
| G25 | 0.92 | 1.43 | 0.96 | 1.02 | 1.41 | G110 | 1.95 | 0.69 | 0.84 | 0.89 | 4.10 |
| G26 | 0.60 | 0.63 | 0.98 | 1.03 | 0.56 | G111 | 1.28 | 0.87 | 0.91 | 0.97 | 2.35 |
| G27 | 1.37 | 1.32 | 0.92 | 0.98 | 2.59 | G112 | 1.17 | 0.86 | 0.92 | 0.98 | 2.06 |
| G28 | 0.15 | 0.83 | 1.02 | 1.09 | -0.61 | G113 | 1.25 | 0.81 | 0.91 | 0.97 | 2.27 |
| G29 | 1.17 | 1.01 | 0.93 | 0.99 | 2.06 | G114 | 0.11 | 1.29 | 1.02 | 1.08 | -0.70 |
| G30 | 0.44 | 1.35 | 1.00 | 1.06 | 0.14 | G115 | 0.68 | 1.27 | 0.98 | 1.03 | 0.79 |
| G31 | 0.30 | 1.02 | 1.01 | 1.07 | -0.22 | G116 | 1.32 | 0.61 | 0.89 | 0.95 | 2.46 |
| G32 | 0.83 | 1.13 | 0.96 | 1.02 | 1.17 | G117 | 1.85 | 0.85 | 0.86 | 0.92 | 3.84 |
| G33 | 1.02 | 1.30 | 0.95 | 1.01 | 1.68 | G118 | 0.50 | 0.86 | 0.99 | 1.05 | 0.31 |
| G34 | 1.65 | 1.21 | 0.90 | 0.95 | 3.32 | G119 | 2.15 | 0.95 | 0.85 | 0.90 | 4.62 |
| G35 | 0.45 | 0.89 | 0.99 | 1.05 | 0.17 | G120 | 2.20 | 1.02 | 0.85 | 0.90 | 4.76 |
| G36 | 1.66 | 1.07 | 0.89 | 0.95 | 3.35 | G121 | 1.39 | 0.73 | 0.90 | 0.95 | 2.63 |
| G37 | 1.12 | 1.00 | 0.93 | 0.99 | 1.94 | G122 | 1.34 | 0.99 | 0.91 | 0.97 | 2.50 |
| G38 | 1.92 | 1.00 | 0.87 | 0.92 | 4.04 | G123 | 1.29 | 0.72 | 0.91 | 0.96 | 2.37 |
| G39 | 0.75 | 0.73 | 0.96 | 1.02 | 0.97 | G124 | 1.15 | 0.97 | 0.93 | 0.99 | 2.00 |
| G40 | 1.05 | 1.01 | 0.94 | 1.00 | 1.76 | G125 | 1.34 | 0.84 | 0.91 | 0.96 | 2.52 |
| G41 | -0.24 | 0.83 | 1.07 | 1.13 | -1.63 | G126 | 1.52 | 0.62 | 0.87 | 0.93 | 2.99 |
| G42 | -0.66 | 0.70 | 1.12 | 1.19 | -2.73 | G127 | 1.60 | 0.89 | 0.89 | 0.94 | 3.19 |
| G43 | 1.03 | 0.86 | 0.94 | 0.99 | 1.70 | G128 | 1.59 | 0.82 | 0.88 | 0.94 | 3.17 |
| G44 | 1.49 | 0.95 | 0.90 | 0.95 | 2.91 | G129 | 2.32 | 1.22 | 0.85 | 0.90 | 5.08 |
| G45 | 0.96 | 1.14 | 0.95 | 1.01 | 1.52 | G130 | 1.96 | 1.23 | 0.88 | 0.93 | 4.13 |
| G46 | 0.81 | 1.31 | 0.97 | 1.02 | 1.12 | G131 | 1.25 | 1.19 | 0.93 | 0.99 | 2.27 |
| G47 | 1.30 | 1.02 | 0.92 | 0.98 | 2.40 | G132 | 0.86 | 0.85 | 0.95 | 1.01 | 1.26 |
| G48 | 0.06 | 0.90 | 1.03 | 1.10 | -0.85 | G133 | 0.16 | 0.92 | 1.02 | 1.08 | -0.57 |
| G49 | -0.42 | 0.89 | 1.08 | 1.15 | -2.11 | G134 | 0.95 | 1.13 | 0.95 | 1.01 | 1.48 |
| G50 | 2.05 | 0.98 | 0.86 | 0.91 | 4.36 | G135 | 1.84 | 1.14 | 0.88 | 0.93 | 3.83 |
| G51 | -0.93 | 0.98 | 1.13 | 1.20 | -3.43 | G136 | 1.06 | 1.10 | 0.94 | 1.00 | 1.79 |
| G52 | 1.40 | 0.91 | 0.91 | 0.96 | 2.67 | G137 | 2.15 | 0.99 | 0.85 | 0.90 | 4.64 |
| G53 | 1.58 | 1.05 | 0.90 | 0.95 | 3.13 | G138 | 0.28 | 0.99 | 1.01 | 1.07 | -0.26 |
| G54 | 1.77 | 1.15 | 0.89 | 0.94 | 3.65 | G139 | -0.27 | 0.73 | 1.07 | 1.14 | -1.72 |
| G55 | 2.05 | 1.15 | 0.87 | 0.92 | 4.38 | G140 | 0.51 | 1.09 | 0.99 | 1.05 | 0.34 |
| G56 | 1.36 | 1.09 | 0.92 | 0.97 | 2.56 | G141 | 1.52 | 0.79 | 0.89 | 0.94 | 2.98 |
| G57 | 0.14 | 1.22 | 1.02 | 1.08 | -0.63 | G142 | 0.48 | 0.99 | 0.99 | 1.05 | 0.26 |
| G58 | -1.85 | 0.78 | 1.26 | 1.34 | -5.85 | G143 | 0.60 | 0.68 | 0.98 | 1.03 | 0.58 |
| G59 | 3.95 | 1.06 | 0.73 | 0.77 | 9.34 | G144 | 0.64 | 0.97 | 0.98 | 1.04 | 0.67 |
| G60 | -0.12 | 0.88 | 1.05 | 1.12 | -1.32 | G145 | -0.25 | 0.97 | 1.06 | 1.13 | -1.65 |
| G61 | 0.06 | 0.89 | 1.03 | 1.10 | -0.85 | G146 | 1.83 | 1.17 | 0.88 | 0.94 | 3.80 |
| G62 | 1.39 | 0.84 | 0.90 | 0.96 | 2.64 | G147 | 1.25 | 1.18 | 0.93 | 0.99 | 2.27 |
| G63 | 1.42 | 1.07 | 0.91 | 0.97 | 2.73 | G148 | 1.17 | 0.71 | 0.92 | 0.97 | 2.06 |
| G64 | 0.63 | 0.66 | 0.97 | 1.03 | 0.65 | G149 | 1.35 | 0.80 | 0.90 | 0.96 | 2.55 |
| G65 | -2.52 | 0.86 | 1.34 | 1.42 | -7.59 | G150 | 0.67 | 0.83 | 0.97 | 1.03 | 0.75 |
| G66 | 1.31 | 1.22 | 0.92 | 0.98 | 2.44 | G151 | -1.26 | 0.69 | 1.20 | 1.27 | -4.31 |
| G67 | 2.32 | 1.23 | 0.85 | 0.90 | 5.07 | G152 | 0.63 | 1.08 | 0.98 | 1.04 | 0.64 |
| G68 | 0.15 | 1.17 | 1.02 | 1.08 | -0.60 | G153 | 1.23 | 1.13 | 0.93 | 0.99 | 2.22 |
| G69 | 1.19 | 1.11 | 0.93 | 0.99 | 2.12 | G154 | 0.68 | 0.81 | 0.97 | 1.03 | 0.77 |
| G70 | 0.88 | 0.96 | 0.95 | 1.01 | 1.31 | G155 | 0.52 | 0.96 | 0.99 | 1.05 | 0.36 |
| G71 | 0.45 | 0.82 | 0.99 | 1.05 | 0.19 | G156 | 0.76 | 0.99 | 0.97 | 1.02 | 0.98 |
| G72 | 2.49 | 1.20 | 0.84 | 0.89 | 5.52 | G157 | 1.37 | 0.97 | 0.91 | 0.97 | 2.58 |
| G73 | 0.17 | 0.73 | 1.02 | 1.09 | -0.56 | G158 | 0.54 | 1.04 | 0.99 | 1.05 | 0.42 |
| G74 | 2.74 | 1.21 | 0.82 | 0.87 | 6.17 | G159 | 1.06 | 1.07 | 0.94 | 1.00 | 1.79 |
| G75 | 0.16 | 1.04 | 1.02 | 1.08 | -0.57 | G160 | 0.97 | 0.93 | 0.94 | 1.00 | 1.55 |
| G76 | 0.52 | 0.83 | 0.99 | 1.05 | 0.35 | G161 | 0.43 | 0.57 | 0.99 | 1.05 | 0.13 |
| G77 | 0.15 | 1.11 | 1.02 | 1.08 | -0.60 | G162 | 0.93 | 0.74 | 0.94 | 1.00 | 1.44 |
| G78 | -0.03 | 1.00 | 1.04 | 1.10 | -1.07 | G163 | 0.52 | 0.67 | 0.98 | 1.04 | 0.36 |
| G79 | 0.94 | 0.70 | 0.94 | 1.00 | 1.46 | G164 | 1.45 | 1.04 | 0.91 | 0.96 | 2.81 |
| G80 | 0.14 | 0.61 | 1.03 | 1.09 | -0.63 | G165 | 1.32 | 0.96 | 0.91 | 0.97 | 2.46 |
| G81 | 0.94 | 0.90 | 0.95 | 1.00 | 1.45 | G166 | 1.29 | 1.06 | 0.92 | 0.98 | 2.37 |
| G82 | 1.06 | 0.96 | 0.94 | 0.99 | 1.79 | G167 | 1.29 | 0.77 | 0.91 | 0.96 | 2.37 |
| G83 | 0.82 | 0.66 | 0.95 | 1.01 | 1.16 | G168 | 1.15 | 1.00 | 0.93 | 0.99 | 2.01 |
| G84 | 1.89 | 1.43 | 0.89 | 0.94 | 3.94 | G169 | 1.28 | 1.01 | 0.92 | 0.98 | 2.35 |
| G85 | 0.78 | 0.65 | 0.95 | 1.01 | 1.05 |  |  |  |  |  |  |

SSI, STI, YSI, RSI, and TOL were the abbreviations of stress susceptibility index, stress tolerance index, yield stability index, relative stress index, tolerance index respectively.

| TABLE S11. Calculated drought tolerance and susceptibility indices in 169 wheat accessions based on the biological yield (BY) indices in 2020-2021. | | | | | | | | | | | |
| --- | --- | --- | --- | --- | --- | --- | --- | --- | --- | --- | --- |
| Code | SSI | STI | YSI | RSI | TOL | Code | SSI | STI | YSI | RSI | TOL |
| G1 | 1.87 | 1.07 | 0.68 | 0.86 | 410.66 | G86 | 0.40 | 0.61 | 0.90 | 1.14 | 87.66 |
| G2 | 1.44 | 0.88 | 0.72 | 0.92 | 316.34 | G87 | 0.89 | 0.87 | 0.81 | 1.04 | 195.00 |
| G3 | 0.96 | 0.59 | 0.77 | 0.98 | 209.00 | G88 | 1.07 | 0.55 | 0.73 | 0.93 | 235.34 |
| G4 | 2.79 | 1.05 | 0.56 | 0.72 | 612.00 | G89 | 1.02 | 0.37 | 0.70 | 0.89 | 224.00 |
| G5 | 1.11 | 0.93 | 0.78 | 1.00 | 242.00 | G90 | -0.12 | 0.79 | 1.03 | 1.31 | -27.00 |
| G6 | 1.28 | 1.14 | 0.77 | 0.98 | 281.00 | G91 | 0.30 | 0.72 | 0.93 | 1.18 | 64.00 |
| G7 | 1.38 | 0.85 | 0.73 | 0.92 | 302.67 | G92 | 0.30 | 0.52 | 0.91 | 1.16 | 66.00 |
| G8 | 2.04 | 0.95 | 0.64 | 0.81 | 448.67 | G93 | 0.35 | 0.67 | 0.91 | 1.16 | 76.67 |
| G9 | 0.66 | 0.43 | 0.81 | 1.03 | 145.00 | G94 | 0.45 | 0.82 | 0.90 | 1.15 | 97.00 |
| G10 | 1.37 | 0.83 | 0.72 | 0.92 | 300.67 | G95 | 0.45 | 1.65 | 0.93 | 1.18 | 98.00 |
| G11 | 2.21 | 0.95 | 0.62 | 0.79 | 485.34 | G96 | 1.23 | 0.64 | 0.72 | 0.92 | 270.34 |
| G12 | 2.55 | 1.60 | 0.65 | 0.83 | 560.00 | G97 | 1.29 | 0.96 | 0.75 | 0.96 | 283.00 |
| G13 | 1.65 | 0.33 | 0.55 | 0.70 | 361.34 | G98 | 0.71 | 0.82 | 0.85 | 1.08 | 155.34 |
| G14 | 1.65 | 0.40 | 0.57 | 0.73 | 361.00 | G99 | 0.56 | 0.93 | 0.88 | 1.12 | 122.00 |
| G15 | 1.43 | 1.09 | 0.75 | 0.95 | 314.00 | G100 | 0.42 | 0.60 | 0.89 | 1.13 | 92.00 |
| G16 | 1.27 | 0.76 | 0.73 | 0.93 | 278.34 | G101 | 1.57 | 2.01 | 0.79 | 1.00 | 343.66 |
| G17 | 0.41 | 0.46 | 0.88 | 1.12 | 89.67 | G102 | 0.52 | 1.06 | 0.90 | 1.14 | 114.33 |
| G18 | 1.34 | 0.68 | 0.71 | 0.90 | 293.33 | G103 | 1.53 | 0.99 | 0.72 | 0.91 | 336.33 |
| G19 | 2.47 | 0.68 | 0.53 | 0.68 | 543.00 | G104 | -0.19 | 0.46 | 1.06 | 1.35 | -42.00 |
| G20 | 1.07 | 0.34 | 0.68 | 0.86 | 233.67 | G105 | 1.01 | 0.53 | 0.74 | 0.95 | 221.67 |
| G21 | 1.70 | 0.99 | 0.69 | 0.88 | 373.00 | G106 | 0.46 | 0.25 | 0.82 | 1.04 | 100.66 |
| G22 | 2.31 | 1.11 | 0.63 | 0.80 | 506.00 | G107 | 1.43 | 0.68 | 0.69 | 0.88 | 314.34 |
| G23 | 1.02 | 0.86 | 0.79 | 1.01 | 222.33 | G108 | 1.37 | 1.06 | 0.75 | 0.96 | 299.67 |
| G24 | 1.73 | 0.61 | 0.62 | 0.80 | 378.66 | G109 | 0.85 | 0.51 | 0.77 | 0.99 | 187.00 |
| G25 | 1.69 | 0.83 | 0.67 | 0.86 | 370.00 | G110 | 1.15 | 0.95 | 0.78 | 0.99 | 252.33 |
| G26 | 1.35 | 0.64 | 0.70 | 0.89 | 296.33 | G111 | 1.12 | 0.85 | 0.77 | 0.98 | 244.33 |
| G27 | 0.98 | 0.81 | 0.79 | 1.01 | 214.00 | G112 | 1.07 | 0.85 | 0.78 | 0.99 | 233.67 |
| G28 | 1.82 | 1.01 | 0.68 | 0.86 | 399.33 | G113 | 0.27 | 0.75 | 0.94 | 1.19 | 58.66 |
| G29 | 0.60 | 0.45 | 0.83 | 1.05 | 130.00 | G114 | 0.82 | 0.79 | 0.82 | 1.04 | 180.00 |
| G30 | 1.56 | 1.34 | 0.75 | 0.95 | 341.67 | G115 | 1.12 | 0.58 | 0.73 | 0.93 | 245.33 |
| G31 | 1.22 | 0.41 | 0.67 | 0.85 | 267.00 | G116 | 1.28 | 0.92 | 0.75 | 0.96 | 281.33 |
| G32 | 0.67 | 0.45 | 0.81 | 1.03 | 147.33 | G117 | 0.74 | 0.61 | 0.82 | 1.04 | 162.66 |
| G33 | 0.55 | 1.20 | 0.90 | 1.14 | 120.66 | G118 | 0.74 | 1.10 | 0.86 | 1.10 | 161.33 |
| G34 | 0.58 | 0.56 | 0.85 | 1.08 | 125.67 | G119 | 0.70 | 0.57 | 0.82 | 1.04 | 154.00 |
| G35 | 1.32 | 1.20 | 0.77 | 0.98 | 290.00 | G120 | 0.59 | 0.48 | 0.83 | 1.06 | 129.33 |
| G36 | 0.49 | 0.46 | 0.86 | 1.09 | 106.00 | G121 | 0.62 | 0.54 | 0.84 | 1.06 | 135.34 |
| G37 | 1.07 | 0.62 | 0.75 | 0.95 | 233.33 | G122 | 0.30 | 0.67 | 0.93 | 1.18 | 64.00 |
| G38 | 0.67 | 0.44 | 0.81 | 1.03 | 146.33 | G123 | 0.48 | 0.82 | 0.89 | 1.14 | 105.00 |
| G39 | 0.89 | 1.57 | 0.86 | 1.09 | 195.33 | G124 | 0.65 | 1.09 | 0.87 | 1.11 | 143.00 |
| G40 | 1.10 | 0.65 | 0.75 | 0.95 | 240.67 | G125 | 0.93 | 1.76 | 0.86 | 1.09 | 204.00 |
| G41 | 1.66 | 1.97 | 0.78 | 0.99 | 363.00 | G126 | 0.71 | 1.58 | 0.89 | 1.13 | 155.67 |
| G42 | 1.55 | 0.81 | 0.69 | 0.88 | 340.67 | G127 | 0.78 | 1.64 | 0.88 | 1.12 | 170.33 |
| G43 | 1.35 | 1.23 | 0.77 | 0.98 | 295.00 | G128 | 1.00 | 0.75 | 0.78 | 0.99 | 219.67 |
| G44 | 0.48 | 0.20 | 0.80 | 1.02 | 104.00 | G129 | 0.75 | 0.98 | 0.85 | 1.08 | 163.00 |
| G45 | 1.75 | 0.64 | 0.63 | 0.80 | 384.33 | G130 | 0.65 | 0.42 | 0.81 | 1.03 | 141.00 |
| G46 | 0.48 | 0.45 | 0.86 | 1.09 | 104.34 | G131 | 0.57 | 0.90 | 0.88 | 1.12 | 124.34 |
| G47 | 0.07 | 0.19 | 0.97 | 1.23 | 15.00 | G132 | 1.15 | 1.08 | 0.79 | 1.00 | 252.00 |
| G48 | 1.30 | 0.98 | 0.75 | 0.96 | 285.33 | G133 | 0.20 | 1.15 | 0.96 | 1.22 | 42.33 |
| G49 | 1.54 | 0.99 | 0.72 | 0.91 | 337.00 | G134 | 1.12 | 1.08 | 0.79 | 1.01 | 245.67 |
| G50 | 1.63 | 0.98 | 0.70 | 0.89 | 358.33 | G135 | 0.42 | 0.36 | 0.86 | 1.10 | 90.34 |
| G51 | 0.89 | 0.60 | 0.78 | 0.99 | 195.66 | G136 | 0.88 | 0.67 | 0.79 | 1.01 | 192.33 |
| G52 | 1.14 | 0.61 | 0.73 | 0.93 | 248.67 | G137 | -0.03 | 0.49 | 1.01 | 1.29 | -8.34 |
| G53 | 0.51 | 0.27 | 0.81 | 1.03 | 111.33 | G138 | 0.97 | 0.46 | 0.74 | 0.94 | 212.33 |
| G54 | 0.85 | 1.05 | 0.84 | 1.07 | 185.00 | G139 | 1.47 | 1.09 | 0.74 | 0.94 | 321.66 |
| G55 | 0.77 | 0.88 | 0.84 | 1.07 | 169.00 | G140 | 1.18 | 1.26 | 0.80 | 1.02 | 258.00 |
| G56 | 1.43 | 1.24 | 0.76 | 0.97 | 313.00 | G141 | 1.14 | 1.30 | 0.81 | 1.03 | 250.00 |
| G57 | 0.76 | 0.17 | 0.68 | 0.86 | 165.33 | G142 | 0.94 | 1.50 | 0.85 | 1.08 | 206.00 |
| G58 | 1.90 | 0.68 | 0.61 | 0.78 | 417.34 | G143 | 0.58 | 0.85 | 0.87 | 1.11 | 127.33 |
| G59 | -0.53 | 0.72 | 1.14 | 1.46 | -117.34 | G144 | 0.82 | 1.09 | 0.85 | 1.08 | 178.67 |
| G60 | 1.89 | 0.87 | 0.65 | 0.83 | 415.67 | G145 | 1.39 | 0.53 | 0.66 | 0.85 | 305.67 |
| G61 | 1.08 | 0.23 | 0.62 | 0.79 | 235.67 | G146 | 1.02 | 1.11 | 0.81 | 1.04 | 223.00 |
| G62 | 1.50 | 1.13 | 0.74 | 0.94 | 328.67 | G147 | 0.65 | 1.08 | 0.88 | 1.11 | 141.33 |
| G63 | -0.03 | 0.65 | 1.01 | 1.29 | -8.67 | G148 | 0.79 | 0.33 | 0.75 | 0.95 | 172.34 |
| G64 | 0.68 | 1.20 | 0.88 | 1.11 | 149.34 | G149 | 0.83 | 0.97 | 0.83 | 1.06 | 182.33 |
| G65 | 1.43 | 0.26 | 0.55 | 0.70 | 314.33 | G150 | 1.35 | 1.35 | 0.78 | 0.99 | 296.66 |
| G66 | 0.97 | 1.14 | 0.82 | 1.05 | 212.00 | G151 | 2.10 | 1.11 | 0.65 | 0.83 | 460.67 |
| G67 | 0.81 | 0.81 | 0.83 | 1.05 | 177.00 | G152 | 0.94 | 0.89 | 0.81 | 1.03 | 204.66 |
| G68 | 0.99 | 0.45 | 0.73 | 0.93 | 217.00 | G153 | 1.10 | 0.34 | 0.67 | 0.85 | 240.66 |
| G69 | 0.67 | 0.70 | 0.84 | 1.07 | 147.34 | G154 | 0.55 | 0.62 | 0.86 | 1.09 | 121.00 |
| G70 | 1.46 | 1.11 | 0.74 | 0.95 | 320.00 | G155 | 1.04 | 1.08 | 0.81 | 1.03 | 228.67 |
| G71 | 1.25 | 0.44 | 0.67 | 0.85 | 273.66 | G156 | 1.13 | 1.36 | 0.81 | 1.03 | 248.34 |
| G72 | 0.11 | 0.93 | 0.98 | 1.24 | 23.00 | G157 | 0.67 | 0.46 | 0.81 | 1.03 | 145.34 |
| G73 | 2.31 | 1.46 | 0.66 | 0.85 | 507.67 | G158 | 1.14 | 0.70 | 0.75 | 0.95 | 249.67 |
| G74 | 0.35 | 0.28 | 0.87 | 1.11 | 76.34 | G159 | 0.94 | 1.60 | 0.85 | 1.09 | 206.33 |
| G75 | 0.68 | 0.55 | 0.82 | 1.05 | 149.00 | G160 | 1.19 | 0.45 | 0.69 | 0.87 | 259.67 |
| G76 | 0.14 | 0.50 | 0.96 | 1.22 | 29.66 | G161 | 1.63 | 1.82 | 0.77 | 0.98 | 357.00 |
| G77 | 0.70 | 0.82 | 0.85 | 1.08 | 152.00 | G162 | 0.47 | 0.81 | 0.90 | 1.14 | 101.67 |
| G78 | 1.14 | 0.35 | 0.66 | 0.85 | 250.33 | G163 | 0.93 | 1.08 | 0.83 | 1.05 | 204.00 |
| G79 | 0.99 | 0.46 | 0.73 | 0.93 | 215.67 | G164 | 0.64 | 0.38 | 0.80 | 1.02 | 138.67 |
| G80 | 1.50 | 0.44 | 0.62 | 0.79 | 329.00 | G165 | 0.61 | 1.21 | 0.89 | 1.13 | 133.67 |
| G81 | 0.51 | 1.13 | 0.90 | 1.15 | 112.00 | G166 | 0.87 | 0.62 | 0.79 | 1.01 | 189.33 |
| G82 | 0.37 | 0.78 | 0.92 | 1.16 | 80.00 | G167 | 0.70 | 0.59 | 0.82 | 1.05 | 154.00 |
| G83 | 1.06 | 0.67 | 0.76 | 0.96 | 232.66 | G168 | 1.13 | 0.88 | 0.77 | 0.98 | 247.66 |
| G84 | 0.27 | 0.61 | 0.93 | 1.18 | 58.00 | G169 | 1.08 | 1.11 | 0.80 | 1.02 | 237.00 |
| G85 | 1.68 | 0.84 | 0.68 | 0.86 | 368.67 |  |  |  |  |  |  |
| SSI, STI, YSI, RSI, and TOL were the abbreviations of stress susceptibility index, stress tolerance index, yield stability index, relative stress index, tolerance index respectively.  TABLE S12. Calculated drought tolerance and susceptibility indices in 169 wheat accessions based on the grain yield (GY) indices in 2020-2021. | | | | | | | | | | | |
| Code | SSI | STI | YSI | RSI | TOL | Code | SSI | STI | YSI | RSI | TOL |
| G1 | 1.88 | 0.83 | 0.59 | 0.79 | 139.00 | G86 | 0.47 | 0.54 | 0.85 | 1.14 | 34.34 |
| G2 | 1.22 | 0.60 | 0.67 | 0.90 | 90.00 | G87 | 1.21 | 0.80 | 0.71 | 0.95 | 89.00 |
| G3 | 1.25 | 0.50 | 0.64 | 0.86 | 92.33 | G88 | 1.29 | 0.72 | 0.68 | 0.91 | 95.00 |
| G4 | 2.06 | 1.02 | 0.59 | 0.80 | 153.00 | G89 | 0.92 | 0.28 | 0.64 | 0.86 | 68.00 |
| G5 | 1.50 | 1.00 | 0.68 | 0.92 | 110.67 | G90 | -0.01 | 0.65 | 1.01 | 1.36 | -2.00 |
| G6 | 0.90 | 1.09 | 0.80 | 1.08 | 66.33 | G91 | 0.83 | 0.62 | 0.76 | 1.03 | 60.66 |
| G7 | 2.10 | 1.02 | 0.59 | 0.79 | 155.33 | G92 | 0.59 | 0.56 | 0.82 | 1.10 | 43.34 |
| G8 | 1.62 | 0.82 | 0.63 | 0.85 | 120.00 | G93 | 0.77 | 0.58 | 0.77 | 1.04 | 56.67 |
| G9 | 0.75 | 0.42 | 0.75 | 1.00 | 54.67 | G94 | 1.23 | 0.88 | 0.71 | 0.96 | 90.67 |
| G10 | 0.99 | 0.97 | 0.77 | 1.04 | 72.67 | G95 | 0.67 | 1.33 | 0.86 | 1.16 | 48.67 |
| G11 | 2.10 | 0.87 | 0.56 | 0.76 | 156.00 | G96 | 1.49 | 0.59 | 0.61 | 0.82 | 110.34 |
| G12 | 3.09 | 1.88 | 0.56 | 0.76 | 229.67 | G97 | 2.01 | 1.05 | 0.61 | 0.82 | 148.67 |
| G13 | 0.86 | 0.27 | 0.66 | 0.88 | 63.00 | G98 | 0.25 | 0.65 | 0.93 | 1.25 | 17.33 |
| G14 | 0.99 | 0.35 | 0.65 | 0.88 | 73.00 | G99 | 0.81 | 0.72 | 0.78 | 1.05 | 59.67 |
| G15 | 1.61 | 0.97 | 0.66 | 0.88 | 119.00 | G100 | 0.65 | 0.51 | 0.79 | 1.07 | 47.67 |
| G16 | 1.09 | 0.51 | 0.67 | 0.91 | 80.67 | G101 | 0.90 | 1.98 | 0.85 | 1.14 | 66.33 |
| G17 | 0.61 | 0.50 | 0.80 | 1.08 | 44.67 | G102 | 0.42 | 0.76 | 0.88 | 1.19 | 30.67 |
| G18 | 1.48 | 0.67 | 0.63 | 0.85 | 109.34 | G103 | 0.81 | 0.69 | 0.78 | 1.05 | 59.67 |
| G19 | 1.69 | 0.52 | 0.55 | 0.74 | 125.00 | G104 | 0.14 | 0.44 | 0.95 | 1.28 | 9.67 |
| G20 | 1.12 | 0.33 | 0.61 | 0.82 | 82.67 | G105 | 1.18 | 0.47 | 0.65 | 0.87 | 86.67 |
| G21 | 1.48 | 1.12 | 0.70 | 0.94 | 109.33 | G106 | 0.51 | 0.20 | 0.75 | 1.00 | 37.33 |
| G22 | 1.80 | 0.93 | 0.62 | 0.83 | 133.67 | G107 | 1.30 | 0.54 | 0.64 | 0.86 | 96.00 |
| G23 | 1.61 | 1.04 | 0.67 | 0.90 | 119.00 | G108 | 1.21 | 0.97 | 0.73 | 0.98 | 89.00 |
| G24 | 1.42 | 0.62 | 0.63 | 0.85 | 104.66 | G109 | 0.91 | 0.49 | 0.72 | 0.97 | 67.00 |
| G25 | 1.87 | 1.21 | 0.65 | 0.87 | 138.33 | G110 | 1.39 | 0.67 | 0.65 | 0.87 | 103.00 |
| G26 | 0.98 | 0.41 | 0.68 | 0.91 | 72.00 | G111 | 1.19 | 0.75 | 0.70 | 0.95 | 87.67 |
| G27 | 1.26 | 1.09 | 0.73 | 0.99 | 93.00 | G112 | 1.09 | 0.74 | 0.72 | 0.97 | 80.66 |
| G28 | 1.28 | 0.85 | 0.70 | 0.94 | 94.67 | G113 | 0.49 | 0.61 | 0.85 | 1.15 | 35.67 |
| G29 | 0.70 | 0.46 | 0.77 | 1.04 | 51.33 | G114 | 0.72 | 1.04 | 0.83 | 1.12 | 53.00 |
| G30 | 1.53 | 1.84 | 0.75 | 1.01 | 113.00 | G115 | 1.16 | 0.75 | 0.71 | 0.95 | 85.67 |
| G31 | 1.01 | 0.42 | 0.67 | 0.90 | 74.66 | G116 | 1.14 | 0.57 | 0.68 | 0.91 | 84.33 |
| G32 | 0.72 | 0.51 | 0.77 | 1.04 | 53.00 | G117 | 1.01 | 0.53 | 0.70 | 0.94 | 74.33 |
| G33 | 0.77 | 1.57 | 0.85 | 1.15 | 56.33 | G118 | 0.67 | 0.96 | 0.84 | 1.13 | 48.66 |
| G34 | 0.88 | 0.69 | 0.76 | 1.03 | 64.33 | G119 | 1.08 | 0.56 | 0.69 | 0.93 | 79.33 |
| G35 | 1.09 | 1.07 | 0.76 | 1.03 | 80.66 | G120 | 0.99 | 0.50 | 0.70 | 0.94 | 72.66 |
| G36 | 0.72 | 0.50 | 0.77 | 1.04 | 52.67 | G121 | 0.75 | 0.40 | 0.74 | 0.99 | 55.00 |
| G37 | 1.10 | 0.62 | 0.70 | 0.94 | 81.33 | G122 | 0.52 | 0.67 | 0.85 | 1.14 | 38.00 |
| G38 | 0.93 | 0.44 | 0.70 | 0.94 | 68.67 | G123 | 0.62 | 0.59 | 0.81 | 1.10 | 45.34 |
| G39 | 0.82 | 1.15 | 0.82 | 1.11 | 60.00 | G124 | 0.84 | 1.08 | 0.81 | 1.10 | 61.34 |
| G40 | 1.10 | 0.66 | 0.71 | 0.95 | 81.00 | G125 | 1.18 | 1.49 | 0.78 | 1.05 | 87.33 |
| G41 | 0.92 | 1.67 | 0.83 | 1.12 | 67.67 | G126 | 0.95 | 0.98 | 0.78 | 1.05 | 70.00 |
| G42 | 0.75 | 0.58 | 0.78 | 1.05 | 55.00 | G127 | 1.13 | 1.45 | 0.79 | 1.06 | 83.00 |
| G43 | 1.23 | 1.07 | 0.74 | 0.99 | 91.00 | G128 | 1.20 | 0.63 | 0.68 | 0.91 | 88.33 |
| G44 | 0.58 | 0.20 | 0.72 | 0.97 | 42.00 | G129 | 1.38 | 1.20 | 0.72 | 0.97 | 101.67 |
| G45 | 1.70 | 0.75 | 0.61 | 0.82 | 125.67 | G130 | 0.99 | 0.53 | 0.71 | 0.95 | 72.67 |
| G46 | 0.55 | 0.60 | 0.83 | 1.12 | 40.33 | G131 | 0.81 | 1.08 | 0.82 | 1.10 | 59.67 |
| G47 | 0.20 | 0.20 | 0.90 | 1.21 | 13.67 | G132 | 1.08 | 0.93 | 0.75 | 1.01 | 79.34 |
| G48 | 0.92 | 0.89 | 0.78 | 1.05 | 67.34 | G133 | 0.14 | 1.09 | 0.97 | 1.31 | 9.33 |
| G49 | 0.97 | 0.88 | 0.77 | 1.03 | 71.00 | G134 | 1.22 | 1.24 | 0.76 | 1.02 | 89.67 |
| G50 | 1.89 | 1.00 | 0.62 | 0.83 | 140.34 | G135 | 0.67 | 0.41 | 0.77 | 1.03 | 48.67 |
| G51 | 0.35 | 0.60 | 0.89 | 1.20 | 25.00 | G136 | 0.97 | 0.75 | 0.75 | 1.01 | 71.66 |
| G52 | 1.19 | 0.56 | 0.67 | 0.90 | 87.67 | G137 | 0.43 | 0.49 | 0.86 | 1.15 | 31.00 |
| G53 | 0.67 | 0.29 | 0.73 | 0.98 | 48.66 | G138 | 0.82 | 0.47 | 0.74 | 0.99 | 60.00 |
| G54 | 1.27 | 1.22 | 0.74 | 1.00 | 93.67 | G139 | 0.74 | 0.79 | 0.81 | 1.09 | 54.33 |
| G55 | 1.28 | 1.03 | 0.72 | 0.97 | 94.67 | G140 | 1.08 | 1.38 | 0.79 | 1.06 | 79.67 |
| G56 | 1.64 | 1.37 | 0.70 | 0.94 | 121.00 | G141 | 1.31 | 1.04 | 0.72 | 0.97 | 96.67 |
| G57 | 0.67 | 0.22 | 0.69 | 0.93 | 49.00 | G142 | 0.83 | 1.51 | 0.84 | 1.13 | 60.66 |
| G58 | 0.73 | 0.53 | 0.77 | 1.04 | 53.67 | G143 | 0.50 | 0.58 | 0.85 | 1.14 | 36.00 |
| G59 | 0.63 | 0.77 | 0.83 | 1.12 | 45.67 | G144 | 0.76 | 1.07 | 0.83 | 1.12 | 55.66 |
| G60 | 1.30 | 0.77 | 0.68 | 0.92 | 96.33 | G145 | 0.98 | 0.52 | 0.71 | 0.95 | 72.00 |
| G61 | 0.83 | 0.21 | 0.63 | 0.84 | 61.00 | G146 | 1.47 | 1.32 | 0.72 | 0.97 | 108.66 |
| G62 | 1.56 | 0.97 | 0.67 | 0.90 | 115.34 | G147 | 0.90 | 1.29 | 0.82 | 1.10 | 66.33 |
| G63 | 0.27 | 0.70 | 0.92 | 1.24 | 19.33 | G148 | 0.74 | 0.24 | 0.68 | 0.92 | 54.00 |
| G64 | 0.57 | 0.80 | 0.85 | 1.15 | 41.34 | G149 | 0.96 | 0.78 | 0.76 | 1.02 | 70.67 |
| G65 | 0.59 | 0.22 | 0.73 | 0.98 | 43.34 | G150 | 1.16 | 1.13 | 0.76 | 1.02 | 85.67 |
| G66 | 1.25 | 1.40 | 0.76 | 1.03 | 92.00 | G151 | 0.84 | 0.78 | 0.78 | 1.06 | 61.33 |
| G67 | 1.37 | 1.01 | 0.70 | 0.95 | 101.00 | G152 | 0.90 | 0.96 | 0.79 | 1.07 | 66.00 |
| G68 | 0.86 | 0.53 | 0.74 | 1.00 | 63.00 | G153 | 1.16 | 0.39 | 0.62 | 0.84 | 85.33 |
| G69 | 0.82 | 0.78 | 0.79 | 1.06 | 60.34 | G154 | 0.59 | 0.52 | 0.81 | 1.09 | 42.67 |
| G70 | 1.38 | 1.09 | 0.71 | 0.96 | 102.33 | G155 | 0.93 | 1.05 | 0.79 | 1.07 | 68.67 |
| G71 | 0.97 | 0.36 | 0.66 | 0.89 | 71.00 | G156 | 1.09 | 1.35 | 0.79 | 1.06 | 80.33 |
| G72 | 0.80 | 1.13 | 0.83 | 1.11 | 58.33 | G157 | 0.79 | 0.45 | 0.74 | 1.00 | 57.66 |
| G73 | 1.53 | 1.07 | 0.68 | 0.92 | 113.34 | G158 | 1.03 | 0.74 | 0.74 | 0.99 | 75.67 |
| G74 | 0.82 | 0.34 | 0.70 | 0.94 | 60.00 | G159 | 1.12 | 1.74 | 0.80 | 1.08 | 82.67 |
| G75 | 0.52 | 0.58 | 0.84 | 1.13 | 37.66 | G160 | 1.11 | 0.43 | 0.65 | 0.87 | 81.67 |
| G76 | 0.15 | 0.42 | 0.95 | 1.28 | 10.00 | G161 | 1.04 | 1.04 | 0.77 | 1.04 | 76.34 |
| G77 | 0.52 | 0.92 | 0.87 | 1.17 | 37.66 | G162 | 0.49 | 0.60 | 0.85 | 1.15 | 35.67 |
| G78 | 0.86 | 0.36 | 0.69 | 0.93 | 63.00 | G163 | 0.67 | 0.73 | 0.82 | 1.10 | 48.67 |
| G79 | 0.82 | 0.32 | 0.69 | 0.93 | 60.00 | G164 | 0.80 | 0.40 | 0.72 | 0.98 | 58.33 |
| G80 | 0.94 | 0.27 | 0.63 | 0.85 | 69.33 | G165 | 0.85 | 1.17 | 0.82 | 1.10 | 62.66 |
| G81 | 0.63 | 1.03 | 0.85 | 1.15 | 46.00 | G166 | 1.00 | 0.66 | 0.73 | 0.98 | 73.67 |
| G82 | 0.48 | 0.75 | 0.87 | 1.17 | 34.67 | G167 | 0.77 | 0.46 | 0.75 | 1.01 | 56.33 |
| G83 | 0.86 | 0.45 | 0.72 | 0.97 | 63.00 | G168 | 1.20 | 0.89 | 0.72 | 0.97 | 88.34 |
| G84 | 0.71 | 0.88 | 0.83 | 1.11 | 51.66 | G169 | 1.26 | 1.14 | 0.74 | 0.99 | 93.00 |
| G85 | 1.28 | 0.56 | 0.64 | 0.87 | 94.67 |  |  |  |  |  |  |

SSI, STI, YSI, RSI, and TOL were the abbreviations of stress susceptibility index, stress tolerance index, yield stability index, relative stress index, tolerance index respectively.

| TABLE S13. Calculated drought tolerance and susceptibility indices in 169 wheat accessions based on the chlorophyll estimated using SPAD indices in 2021-2022. | | | | | | | | | | | |
| --- | --- | --- | --- | --- | --- | --- | --- | --- | --- | --- | --- |
| Code | SSI | STI | YSI | RSI | TOL | Code | SSI | STI | YSI | RSI | TOL |
| G1 | 0.39 | 0.65 | 0.86 | 1.32 | 5.26 | G86 | 1.02 | 0.86 | 0.68 | 1.05 | 15.46 |
| G2 | 1.21 | 0.66 | 0.59 | 0.91 | 18.56 | G87 | 0.80 | 0.80 | 0.74 | 1.13 | 11.93 |
| G3 | 1.48 | 0.58 | 0.51 | 0.78 | 22.86 | G88 | 1.73 | 0.50 | 0.43 | 0.66 | 26.83 |
| G4 | 0.99 | 0.71 | 0.67 | 1.02 | 15.03 | G89 | 0.68 | 0.77 | 0.77 | 1.19 | 9.90 |
| G5 | 1.95 | 0.34 | 0.32 | 0.49 | 30.50 | G90 | 1.25 | 0.42 | 0.51 | 0.79 | 19.14 |
| G6 | 1.48 | 0.59 | 0.51 | 0.78 | 22.83 | G91 | 0.72 | 0.72 | 0.75 | 1.15 | 10.60 |
| G7 | 0.32 | 0.55 | 0.88 | 1.35 | 4.20 | G92 | 0.03 | 0.68 | 1.01 | 1.56 | -0.53 |
| G8 | 0.66 | 0.49 | 0.73 | 1.12 | 9.60 | G93 | 0.69 | 0.61 | 0.74 | 1.14 | 10.16 |
| G9 | 0.52 | 0.71 | 0.82 | 1.26 | 7.33 | G94 | 1.44 | 0.43 | 0.46 | 0.71 | 22.30 |
| G10 | 1.24 | 1.13 | 0.67 | 1.02 | 18.93 | G95 | 0.42 | 0.77 | 0.86 | 1.32 | 5.73 |
| G11 | 0.23 | 1.04 | 0.94 | 1.44 | 2.70 | G96 | 0.73 | 0.65 | 0.73 | 1.13 | 10.83 |
| G12 | 0.51 | 0.77 | 0.83 | 1.27 | 7.20 | G97 | 0.07 | 0.57 | 1.00 | 1.53 | 0.13 |
| G13 | 1.42 | 0.61 | 0.53 | 0.81 | 21.96 | G98 | 1.20 | 0.77 | 0.62 | 0.95 | 18.33 |
| G14 | 1.40 | 0.63 | 0.54 | 0.83 | 21.63 | G99 | 1.03 | 0.69 | 0.65 | 1.00 | 15.57 |
| G15 | 1.27 | 0.65 | 0.58 | 0.89 | 19.56 | G100 | 0.07 | 1.03 | 1.00 | 1.53 | 0.13 |
| G16 | 0.86 | 0.93 | 0.74 | 1.13 | 12.83 | G101 | 0.13 | 1.15 | 0.98 | 1.50 | 1.07 |
| G17 | -0.58 | 0.87 | 1.29 | 1.98 | -10.30 | G102 | -0.16 | 0.92 | 1.09 | 1.67 | -3.57 |
| G18 | 0.63 | 0.49 | 0.74 | 1.13 | 9.23 | G103 | 1.42 | 0.68 | 0.55 | 0.84 | 21.86 |
| G19 | 1.30 | 0.60 | 0.55 | 0.85 | 20.03 | G104 | 0.31 | 0.78 | 0.90 | 1.38 | 4.06 |
| G20 | 1.13 | 0.46 | 0.56 | 0.86 | 17.30 | G105 | 1.66 | 0.64 | 0.48 | 0.74 | 25.70 |
| G21 | 0.99 | 0.59 | 0.64 | 0.98 | 15.03 | G106 | 0.55 | 0.48 | 0.77 | 1.18 | 7.92 |
| G22 | 1.25 | 0.46 | 0.53 | 0.81 | 19.16 | G107 | -0.07 | 0.92 | 1.05 | 1.62 | -2.17 |
| G23 | 1.66 | 0.44 | 0.42 | 0.65 | 25.76 | G108 | 0.75 | 0.86 | 0.76 | 1.17 | 11.10 |
| G24 | 1.61 | 0.45 | 0.44 | 0.67 | 24.90 | G109 | 0.88 | 0.66 | 0.69 | 1.06 | 13.17 |
| G25 | -0.05 | 0.76 | 1.05 | 1.61 | -1.74 | G110 | 1.32 | 0.58 | 0.54 | 0.84 | 20.30 |
| G26 | 0.46 | 0.72 | 0.84 | 1.29 | 6.50 | G111 | 1.65 | 0.67 | 0.49 | 0.76 | 25.56 |
| G27 | 1.51 | 0.69 | 0.53 | 0.81 | 23.33 | G112 | 0.87 | 1.12 | 0.75 | 1.16 | 13.07 |
| G28 | 0.47 | 0.71 | 0.83 | 1.28 | 6.63 | G113 | 1.19 | 0.53 | 0.57 | 0.87 | 18.26 |
| G29 | 0.90 | 0.72 | 0.69 | 1.07 | 13.53 | G114 | -0.30 | 0.75 | 1.17 | 1.79 | -5.83 |
| G30 | 1.69 | 0.50 | 0.44 | 0.67 | 26.33 | G115 | 1.27 | 0.47 | 0.53 | 0.81 | 19.53 |
| G31 | 1.39 | 0.71 | 0.56 | 0.86 | 21.43 | G116 | 0.81 | 0.62 | 0.71 | 1.08 | 12.00 |
| G32 | 0.63 | 0.67 | 0.77 | 1.18 | 9.23 | G117 | 0.38 | 0.72 | 0.87 | 1.33 | 5.20 |
| G33 | 0.69 | 0.57 | 0.73 | 1.13 | 10.20 | G118 | 0.82 | 0.47 | 0.66 | 1.02 | 12.27 |
| G34 | 1.32 | 0.51 | 0.53 | 0.81 | 20.23 | G119 | 0.44 | 0.48 | 0.82 | 1.25 | 6.13 |
| G35 | 0.76 | 0.87 | 0.76 | 1.16 | 11.33 | G120 | 1.30 | 0.81 | 0.60 | 0.92 | 20.00 |
| G36 | 1.24 | 0.28 | 0.45 | 0.69 | 18.97 | G121 | 1.92 | 0.72 | 0.45 | 0.69 | 29.93 |
| G37 | 1.41 | 0.64 | 0.54 | 0.83 | 21.80 | G122 | 1.71 | 0.70 | 0.49 | 0.75 | 26.66 |
| G38 | 0.14 | 0.93 | 0.97 | 1.49 | 1.33 | G123 | -0.02 | 0.76 | 1.04 | 1.59 | -1.37 |
| G39 | 0.31 | 0.67 | 0.89 | 1.37 | 4.06 | G124 | 0.48 | 0.61 | 0.82 | 1.26 | 6.80 |
| G40 | 2.06 | 0.46 | 0.35 | 0.54 | 32.26 | G125 | 1.24 | 0.65 | 0.59 | 0.90 | 18.97 |
| G41 | 0.82 | 0.49 | 0.67 | 1.03 | 12.20 | G126 | 0.94 | 0.73 | 0.68 | 1.05 | 14.13 |
| G42 | 0.76 | 0.92 | 0.76 | 1.17 | 11.23 | G127 | 1.34 | 0.51 | 0.52 | 0.80 | 20.57 |
| G43 | 1.77 | 0.37 | 0.37 | 0.57 | 27.63 | G128 | 0.21 | 0.78 | 0.94 | 1.44 | 2.40 |
| G44 | 0.75 | 0.61 | 0.72 | 1.11 | 11.03 | G129 | 0.54 | 0.71 | 0.81 | 1.25 | 7.63 |
| G45 | 1.68 | 0.74 | 0.50 | 0.77 | 26.17 | G130 | 1.17 | 0.74 | 0.62 | 0.95 | 17.93 |
| G46 | 1.06 | 0.52 | 0.60 | 0.92 | 16.10 | G131 | 0.67 | 0.74 | 0.77 | 1.18 | 9.80 |
| G47 | 2.25 | 0.43 | 0.31 | 0.48 | 35.27 | G132 | 0.92 | 0.45 | 0.62 | 0.96 | 13.77 |
| G48 | 2.22 | 0.51 | 0.34 | 0.53 | 34.73 | G133 | 0.72 | 0.49 | 0.71 | 1.08 | 10.60 |
| G49 | 1.85 | 0.62 | 0.44 | 0.68 | 28.77 | G134 | 1.02 | 1.05 | 0.71 | 1.08 | 15.53 |
| G50 | 1.48 | 0.44 | 0.46 | 0.71 | 22.80 | G135 | 1.00 | 0.57 | 0.63 | 0.97 | 15.20 |
| G51 | 0.58 | 0.83 | 0.81 | 1.24 | 8.36 | G136 | 1.19 | 0.60 | 0.59 | 0.90 | 18.13 |
| G52 | 1.02 | 0.75 | 0.66 | 1.02 | 15.46 | G137 | 1.63 | 0.71 | 0.51 | 0.78 | 25.33 |
| G53 | 1.66 | 0.42 | 0.41 | 0.63 | 25.80 | G138 | 0.93 | 0.88 | 0.71 | 1.09 | 13.93 |
| G54 | 0.56 | 0.47 | 0.77 | 1.18 | 7.96 | G139 | 1.60 | 0.52 | 0.46 | 0.71 | 24.76 |
| G55 | 0.43 | 0.80 | 0.86 | 1.32 | 6.00 | G140 | 0.03 | 0.67 | 1.02 | 1.56 | -0.57 |
| G56 | 1.50 | 0.40 | 0.44 | 0.68 | 23.13 | G141 | 0.95 | 0.77 | 0.69 | 1.06 | 14.30 |
| G57 | 1.61 | 0.52 | 0.46 | 0.71 | 25.03 | G142 | 0.91 | 0.47 | 0.64 | 0.98 | 13.67 |
| G58 | 0.92 | 0.72 | 0.69 | 1.06 | 13.86 | G143 | 0.87 | 0.63 | 0.69 | 1.05 | 13.07 |
| G59 | 0.62 | 0.64 | 0.77 | 1.18 | 9.06 | G144 | 1.07 | 0.80 | 0.66 | 1.01 | 16.30 |
| G60 | 0.85 | 0.80 | 0.72 | 1.11 | 12.63 | G145 | 1.12 | 0.74 | 0.63 | 0.97 | 17.13 |
| G61 | 0.54 | 0.57 | 0.79 | 1.22 | 7.63 | G146 | 0.94 | 0.46 | 0.62 | 0.95 | 14.13 |
| G62 | -0.22 | 0.93 | 1.12 | 1.71 | -4.57 | G147 | 0.59 | 0.75 | 0.80 | 1.22 | 8.57 |
| G63 | 1.28 | 0.60 | 0.56 | 0.86 | 19.67 | G148 | 2.26 | 0.40 | 0.30 | 0.45 | 35.43 |
| G64 | 1.36 | 0.78 | 0.58 | 0.90 | 20.87 | G149 | 0.99 | 0.62 | 0.65 | 0.99 | 15.00 |
| G65 | 0.89 | 0.63 | 0.68 | 1.04 | 13.43 | G150 | 1.20 | 0.57 | 0.58 | 0.88 | 18.30 |
| G66 | 0.55 | 0.71 | 0.81 | 1.24 | 7.80 | G151 | 1.90 | 0.69 | 0.45 | 0.69 | 29.60 |
| G67 | 1.68 | 0.43 | 0.41 | 0.63 | 26.07 | G152 | 0.82 | 0.88 | 0.74 | 1.14 | 12.26 |
| G68 | 0.77 | 0.86 | 0.75 | 1.15 | 11.50 | G153 | 1.58 | 0.49 | 0.45 | 0.70 | 24.53 |
| G69 | 0.62 | 0.67 | 0.77 | 1.19 | 9.06 | G154 | 0.89 | 0.60 | 0.67 | 1.03 | 13.33 |
| G70 | 1.51 | 0.46 | 0.46 | 0.71 | 23.36 | G155 | 1.76 | 0.49 | 0.42 | 0.64 | 27.36 |
| G71 | 1.18 | 0.90 | 0.65 | 1.00 | 18.00 | G156 | 1.36 | 0.57 | 0.53 | 0.82 | 20.90 |
| G72 | 0.35 | 0.78 | 0.89 | 1.36 | 4.60 | G157 | 0.93 | 0.46 | 0.62 | 0.96 | 13.93 |
| G73 | 0.20 | 0.73 | 0.94 | 1.45 | 2.17 | G158 | 0.92 | 0.67 | 0.68 | 1.04 | 13.84 |
| G74 | -0.11 | 0.74 | 1.08 | 1.65 | -2.77 | G159 | 1.42 | 0.35 | 0.43 | 0.67 | 21.97 |
| G75 | 1.45 | 0.55 | 0.50 | 0.77 | 22.40 | G160 | 1.12 | 0.75 | 0.64 | 0.98 | 17.13 |
| G76 | 1.15 | 0.43 | 0.54 | 0.83 | 17.57 | G161 | -0.05 | 0.61 | 1.05 | 1.62 | -1.77 |
| G77 | -0.04 | 0.74 | 1.04 | 1.60 | -1.60 | G162 | 1.93 | 0.32 | 0.32 | 0.48 | 30.06 |
| G78 | 1.84 | 0.41 | 0.37 | 0.57 | 28.73 | G163 | 0.97 | 0.74 | 0.68 | 1.04 | 14.70 |
| G79 | 1.76 | 0.49 | 0.42 | 0.64 | 27.40 | G164 | 1.58 | 0.62 | 0.50 | 0.76 | 24.53 |
| G80 | 1.37 | 0.56 | 0.53 | 0.81 | 21.03 | G165 | 1.48 | 0.80 | 0.56 | 0.86 | 22.90 |
| G81 | 0.76 | 0.87 | 0.76 | 1.16 | 11.33 | G166 | 1.65 | 0.55 | 0.46 | 0.70 | 25.67 |
| G82 | 2.12 | 0.26 | 0.25 | 0.38 | 33.26 | G167 | 0.73 | 0.77 | 0.75 | 1.16 | 10.80 |
| G83 | 1.51 | 0.52 | 0.48 | 0.74 | 23.30 | G168 | 1.83 | 0.30 | 0.32 | 0.49 | 28.56 |
| G84 | 1.38 | 0.74 | 0.57 | 0.88 | 21.33 | G169 | 0.47 | 0.76 | 0.84 | 1.29 | 6.53 |
| G85 | 1.22 | 0.73 | 0.61 | 0.93 | 18.63 |  |  |  |  |  |  |

SSI, STI, YSI, RSI, and TOL were the abbreviations of stress susceptibility index, stress tolerance index, yield stability index, relative stress index, tolerance index respectively.

| TABLE S14. Calculated drought tolerance and susceptibility indices in 169 wheat accessions based on the relative water content (RWC) indices in 2021-2022. | | | | | | | | | | | |
| --- | --- | --- | --- | --- | --- | --- | --- | --- | --- | --- | --- |
| Code | SSI | STI | YSI | RSI | TOL | Code | SSI | STI | YSI | RSI | TOL |
| G1 | 1.03 | 0.90 | 0.73 | 0.98 | 0.22 | G86 | 0.89 | 0.63 | 0.90 | 1.22 | 0.06 |
| G2 | 1.00 | 0.69 | 0.73 | 0.99 | 0.19 | G87 | 1.08 | 0.75 | 0.64 | 0.87 | 0.28 |
| G3 | 1.10 | 0.61 | 0.58 | 0.79 | 0.31 | G88 | 1.00 | 0.89 | 0.76 | 1.03 | 0.19 |
| G4 | 1.06 | 0.76 | 0.66 | 0.90 | 0.26 | G89 | 1.03 | 0.40 | 0.62 | 0.84 | 0.22 |
| G5 | 0.96 | 0.99 | 0.82 | 1.12 | 0.14 | G90 | 1.01 | 0.52 | 0.68 | 0.93 | 0.20 |
| G6 | 0.98 | 0.92 | 0.78 | 1.06 | 0.17 | G91 | 0.95 | 0.79 | 0.82 | 1.11 | 0.13 |
| G7 | 1.03 | 0.83 | 0.71 | 0.96 | 0.23 | G92 | 0.89 | 0.84 | 0.91 | 1.24 | 0.06 |
| G8 | 0.98 | 0.92 | 0.78 | 1.06 | 0.17 | G93 | 0.90 | 0.80 | 0.90 | 1.22 | 0.07 |
| G9 | 0.98 | 0.46 | 0.71 | 0.96 | 0.17 | G94 | 1.03 | 0.84 | 0.72 | 0.98 | 0.22 |
| G10 | 0.90 | 0.96 | 0.91 | 1.23 | 0.07 | G95 | 0.97 | 0.88 | 0.79 | 1.07 | 0.16 |
| G11 | 1.08 | 0.79 | 0.64 | 0.87 | 0.29 | G96 | 1.05 | 0.72 | 0.67 | 0.91 | 0.25 |
| G12 | 1.18 | 1.25 | 0.61 | 0.83 | 0.40 | G97 | 0.99 | 1.04 | 0.78 | 1.06 | 0.18 |
| G13 | 1.08 | 0.37 | 0.53 | 0.72 | 0.28 | G98 | 0.96 | 0.76 | 0.80 | 1.09 | 0.14 |
| G14 | 1.06 | 0.45 | 0.59 | 0.80 | 0.26 | G99 | 1.05 | 0.65 | 0.65 | 0.89 | 0.25 |
| G15 | 1.08 | 0.78 | 0.65 | 0.88 | 0.28 | G100 | 0.89 | 0.63 | 0.90 | 1.22 | 0.06 |
| G16 | 0.97 | 0.64 | 0.76 | 1.03 | 0.16 | G101 | 0.95 | 1.21 | 0.85 | 1.15 | 0.13 |
| G17 | 0.97 | 0.68 | 0.76 | 1.04 | 0.16 | G102 | 1.08 | 0.68 | 0.63 | 0.85 | 0.28 |
| G18 | 1.09 | 0.77 | 0.63 | 0.85 | 0.30 | G103 | 1.08 | 0.68 | 0.63 | 0.85 | 0.28 |
| G19 | 1.10 | 0.71 | 0.60 | 0.82 | 0.31 | G104 | 0.90 | 0.66 | 0.89 | 1.21 | 0.07 |
| G20 | 1.08 | 0.41 | 0.54 | 0.73 | 0.29 | G105 | 0.92 | 0.57 | 0.85 | 1.15 | 0.09 |
| G21 | 0.99 | 0.85 | 0.76 | 1.04 | 0.18 | G106 | 0.98 | 0.30 | 0.65 | 0.89 | 0.17 |
| G22 | 0.99 | 0.88 | 0.77 | 1.04 | 0.18 | G107 | 1.04 | 0.69 | 0.67 | 0.91 | 0.24 |
| G23 | 1.08 | 0.80 | 0.65 | 0.88 | 0.28 | G108 | 1.05 | 0.77 | 0.68 | 0.92 | 0.25 |
| G24 | 1.12 | 0.75 | 0.59 | 0.81 | 0.33 | G109 | 0.99 | 0.73 | 0.75 | 1.01 | 0.18 |
| G25 | 1.03 | 0.91 | 0.72 | 0.97 | 0.23 | G110 | 1.10 | 0.66 | 0.59 | 0.80 | 0.31 |
| G26 | 1.04 | 0.51 | 0.63 | 0.86 | 0.24 | G111 | 1.03 | 0.70 | 0.68 | 0.93 | 0.23 |
| G27 | 0.95 | 0.92 | 0.83 | 1.13 | 0.13 | G112 | 1.10 | 0.68 | 0.60 | 0.81 | 0.31 |
| G28 | 1.12 | 0.75 | 0.59 | 0.81 | 0.33 | G113 | 0.89 | 0.74 | 0.91 | 1.23 | 0.06 |
| G29 | 0.91 | 0.62 | 0.87 | 1.18 | 0.08 | G114 | 0.99 | 0.93 | 0.77 | 1.05 | 0.18 |
| G30 | 0.98 | 1.21 | 0.81 | 1.10 | 0.17 | G115 | 1.04 | 0.81 | 0.69 | 0.94 | 0.24 |
| G31 | 0.98 | 0.48 | 0.71 | 0.97 | 0.17 | G116 | 1.05 | 0.65 | 0.65 | 0.89 | 0.25 |
| G32 | 0.97 | 0.67 | 0.78 | 1.05 | 0.15 | G117 | 0.95 | 0.67 | 0.80 | 1.09 | 0.13 |
| G33 | 0.97 | 1.00 | 0.81 | 1.10 | 0.15 | G118 | 0.91 | 0.76 | 0.88 | 1.20 | 0.08 |
| G34 | 0.97 | 0.85 | 0.79 | 1.07 | 0.16 | G119 | 1.06 | 0.66 | 0.64 | 0.87 | 0.26 |
| G35 | 1.01 | 0.87 | 0.74 | 1.01 | 0.20 | G120 | 1.03 | 0.56 | 0.67 | 0.91 | 0.22 |
| G36 | 0.97 | 0.59 | 0.75 | 1.02 | 0.16 | G121 | 0.97 | 0.61 | 0.75 | 1.02 | 0.16 |
| G37 | 1.01 | 0.68 | 0.71 | 0.97 | 0.20 | G122 | 1.08 | 0.63 | 0.62 | 0.84 | 0.28 |
| G38 | 1.04 | 0.51 | 0.63 | 0.86 | 0.24 | G123 | 0.92 | 0.67 | 0.84 | 1.15 | 0.10 |
| G39 | 0.87 | 1.01 | 0.96 | 1.30 | 0.03 | G124 | 0.96 | 0.88 | 0.81 | 1.10 | 0.14 |
| G40 | 0.98 | 0.81 | 0.77 | 1.05 | 0.17 | G125 | 0.94 | 0.99 | 0.85 | 1.15 | 0.12 |
| G41 | 0.91 | 1.08 | 0.90 | 1.22 | 0.08 | G126 | 0.98 | 0.74 | 0.76 | 1.03 | 0.17 |
| G42 | 0.97 | 0.74 | 0.79 | 1.07 | 0.15 | G127 | 0.98 | 0.89 | 0.78 | 1.06 | 0.17 |
| G43 | 0.97 | 0.93 | 0.79 | 1.08 | 0.16 | G128 | 1.00 | 0.81 | 0.75 | 1.01 | 0.19 |
| G44 | 1.00 | 0.30 | 0.62 | 0.84 | 0.19 | G129 | 1.01 | 0.93 | 0.75 | 1.02 | 0.20 |
| G45 | 1.12 | 0.78 | 0.60 | 0.81 | 0.33 | G130 | 1.00 | 0.74 | 0.74 | 1.00 | 0.19 |
| G46 | 1.03 | 0.69 | 0.69 | 0.94 | 0.22 | G131 | 0.94 | 0.97 | 0.84 | 1.15 | 0.12 |
| G47 | 0.92 | 0.33 | 0.80 | 1.09 | 0.09 | G132 | 1.13 | 0.87 | 0.60 | 0.81 | 0.35 |
| G48 | 1.07 | 0.82 | 0.66 | 0.90 | 0.27 | G133 | 0.81 | 0.87 | 1.06 | 1.44 | -0.04 |
| G49 | 1.01 | 1.01 | 0.76 | 1.03 | 0.20 | G134 | 0.87 | 1.01 | 0.96 | 1.30 | 0.03 |
| G50 | 1.06 | 0.97 | 0.69 | 0.94 | 0.26 | G135 | 0.94 | 0.60 | 0.81 | 1.10 | 0.12 |
| G51 | 0.91 | 0.74 | 0.88 | 1.19 | 0.08 | G136 | 1.05 | 0.93 | 0.70 | 0.95 | 0.25 |
| G52 | 1.04 | 0.67 | 0.67 | 0.91 | 0.24 | G137 | 0.87 | 0.68 | 0.95 | 1.29 | 0.03 |
| G53 | 1.05 | 0.41 | 0.58 | 0.79 | 0.25 | G138 | 0.99 | 0.61 | 0.73 | 0.99 | 0.18 |
| G54 | 1.03 | 0.85 | 0.71 | 0.96 | 0.23 | G139 | 0.97 | 0.87 | 0.80 | 1.09 | 0.15 |
| G55 | 1.10 | 0.76 | 0.61 | 0.83 | 0.31 | G140 | 0.96 | 0.96 | 0.82 | 1.11 | 0.14 |
| G56 | 0.93 | 1.12 | 0.87 | 1.18 | 0.11 | G141 | 1.04 | 0.79 | 0.69 | 0.94 | 0.24 |
| G57 | 1.02 | 0.33 | 0.60 | 0.82 | 0.21 | G142 | 0.98 | 1.00 | 0.79 | 1.07 | 0.17 |
| G58 | 0.85 | 0.71 | 0.98 | 1.34 | 0.01 | G143 | 0.96 | 0.71 | 0.79 | 1.08 | 0.14 |
| G59 | 1.09 | 0.70 | 0.61 | 0.83 | 0.30 | G144 | 0.97 | 0.89 | 0.80 | 1.09 | 0.15 |
| G60 | 1.03 | 0.88 | 0.71 | 0.97 | 0.23 | G145 | 1.08 | 0.66 | 0.62 | 0.84 | 0.28 |
| G61 | 1.00 | 0.28 | 0.61 | 0.83 | 0.19 | G146 | 1.04 | 0.92 | 0.71 | 0.96 | 0.24 |
| G62 | 1.02 | 1.08 | 0.76 | 1.03 | 0.21 | G147 | 0.92 | 1.20 | 0.88 | 1.20 | 0.10 |
| G63 | 0.89 | 0.89 | 0.92 | 1.24 | 0.06 | G148 | 0.98 | 0.33 | 0.67 | 0.91 | 0.17 |
| G64 | 1.05 | 0.68 | 0.66 | 0.89 | 0.25 | G149 | 1.07 | 0.79 | 0.66 | 0.89 | 0.27 |
| G65 | 1.00 | 0.35 | 0.64 | 0.87 | 0.19 | G150 | 1.00 | 0.91 | 0.76 | 1.03 | 0.19 |
| G66 | 1.00 | 1.00 | 0.77 | 1.04 | 0.19 | G151 | 1.01 | 0.80 | 0.73 | 1.00 | 0.20 |
| G67 | 0.98 | 0.84 | 0.77 | 1.05 | 0.17 | G152 | 1.03 | 0.91 | 0.72 | 0.97 | 0.23 |
| G68 | 0.99 | 0.70 | 0.74 | 1.01 | 0.18 | G153 | 1.01 | 0.43 | 0.66 | 0.89 | 0.20 |
| G69 | 1.02 | 0.81 | 0.72 | 0.98 | 0.21 | G154 | 0.94 | 0.71 | 0.82 | 1.12 | 0.12 |
| G70 | 1.04 | 0.92 | 0.71 | 0.96 | 0.24 | G155 | 0.99 | 0.88 | 0.77 | 1.04 | 0.18 |
| G71 | 1.00 | 0.42 | 0.67 | 0.91 | 0.19 | G156 | 0.99 | 0.98 | 0.78 | 1.06 | 0.18 |
| G72 | 0.97 | 0.98 | 0.81 | 1.10 | 0.15 | G157 | 0.98 | 0.44 | 0.70 | 0.95 | 0.17 |
| G73 | 0.98 | 0.84 | 0.77 | 1.05 | 0.17 | G158 | 1.06 | 0.78 | 0.67 | 0.91 | 0.26 |
| G74 | 0.98 | 0.42 | 0.70 | 0.95 | 0.17 | G159 | 0.97 | 1.06 | 0.82 | 1.11 | 0.15 |
| G75 | 0.92 | 0.75 | 0.87 | 1.18 | 0.09 | G160 | 1.10 | 0.43 | 0.52 | 0.71 | 0.31 |
| G76 | 0.99 | 0.45 | 0.69 | 0.94 | 0.18 | G161 | 0.99 | 0.98 | 0.78 | 1.06 | 0.18 |
| G77 | 0.96 | 1.05 | 0.83 | 1.12 | 0.14 | G162 | 0.99 | 0.75 | 0.75 | 1.02 | 0.18 |
| G78 | 1.08 | 0.49 | 0.57 | 0.77 | 0.29 | G163 | 1.02 | 0.78 | 0.72 | 0.98 | 0.21 |
| G79 | 0.98 | 0.37 | 0.68 | 0.92 | 0.17 | G164 | 1.08 | 0.43 | 0.55 | 0.74 | 0.29 |
| G80 | 1.06 | 0.41 | 0.57 | 0.78 | 0.26 | G165 | 0.87 | 1.03 | 0.95 | 1.29 | 0.04 |
| G81 | 0.99 | 0.80 | 0.76 | 1.03 | 0.18 | G166 | 0.95 | 0.90 | 0.83 | 1.12 | 0.13 |
| G82 | 0.96 | 0.71 | 0.79 | 1.08 | 0.14 | G167 | 0.90 | 0.55 | 0.88 | 1.19 | 0.07 |
| G83 | 0.97 | 0.50 | 0.75 | 1.01 | 0.15 | G168 | 1.08 | 0.81 | 0.64 | 0.87 | 0.29 |
| G84 | 0.93 | 0.98 | 0.86 | 1.16 | 0.11 | G169 | 1.04 | 0.84 | 0.70 | 0.95 | 0.24 |
| G85 | 1.02 | 0.71 | 0.71 | 0.96 | 0.21 |  |  |  |  |  |  |

SSI, STI, YSI, RSI, and TOL were the abbreviations of stress susceptibility index, stress tolerance index, yield stability index, relative stress index, tolerance index respectively.

| TABLE S15. Calculated drought tolerance and susceptibility indices in 169 wheat accessions based on the plant height (PH) indices in 2021-2022. | | | | | | | | | | | |
| --- | --- | --- | --- | --- | --- | --- | --- | --- | --- | --- | --- |
| Code | SSI | STI | YSI | RSI | TOL | Code | SSI | STI | YSI | RSI | TOL |
| G1 | 0.79 | 0.91 | 0.93 | 1.02 | 7.06 | G86 | 0.58 | 0.58 | 0.94 | 1.03 | 4.93 |
| G2 | 1.15 | 1.26 | 0.91 | 1.00 | 10.67 | G87 | 1.63 | 0.87 | 0.84 | 0.93 | 15.53 |
| G3 | 0.78 | 0.67 | 0.92 | 1.01 | 6.93 | G88 | 0.49 | 0.97 | 0.96 | 1.06 | 4.00 |
| G4 | 2.14 | 1.12 | 0.82 | 0.90 | 20.73 | G89 | 0.51 | 0.79 | 0.95 | 1.05 | 4.13 |
| G5 | 2.26 | 1.07 | 0.81 | 0.89 | 21.93 | G90 | 1.02 | 0.78 | 0.90 | 0.99 | 9.40 |
| G6 | 1.40 | 0.70 | 0.85 | 0.94 | 13.20 | G91 | 1.04 | 0.78 | 0.90 | 0.99 | 9.53 |
| G7 | 1.45 | 0.93 | 0.87 | 0.96 | 13.67 | G92 | 0.72 | 0.69 | 0.93 | 1.02 | 6.27 |
| G8 | 1.26 | 1.10 | 0.89 | 0.98 | 11.80 | G93 | 0.55 | 0.84 | 0.95 | 1.05 | 4.60 |
| G9 | 1.13 | 1.45 | 0.92 | 1.01 | 10.47 | G94 | 0.89 | 1.03 | 0.92 | 1.02 | 8.00 |
| G10 | 0.60 | 0.45 | 0.93 | 1.02 | 5.06 | G95 | 0.97 | 0.98 | 0.91 | 1.01 | 8.80 |
| G11 | 1.07 | 1.21 | 0.91 | 1.01 | 9.86 | G96 | 1.02 | 1.17 | 0.92 | 1.01 | 9.34 |
| G12 | 0.52 | 0.53 | 0.94 | 1.04 | 4.27 | G97 | 0.98 | 1.31 | 0.92 | 1.02 | 8.94 |
| G13 | 0.65 | 0.90 | 0.94 | 1.04 | 5.60 | G98 | 0.58 | 0.57 | 0.94 | 1.03 | 4.93 |
| G14 | 2.64 | 0.93 | 0.76 | 0.84 | 25.80 | G99 | 1.04 | 1.28 | 0.92 | 1.01 | 9.53 |
| G15 | 2.12 | 1.15 | 0.82 | 0.91 | 20.54 | G100 | 1.28 | 1.04 | 0.89 | 0.98 | 12.00 |
| G16 | 1.29 | 1.09 | 0.89 | 0.98 | 12.07 | G101 | 0.72 | 1.41 | 0.95 | 1.04 | 6.33 |
| G17 | 0.85 | 0.45 | 0.89 | 0.98 | 7.67 | G102 | 1.00 | 0.88 | 0.91 | 1.00 | 9.20 |
| G18 | 1.34 | 0.90 | 0.87 | 0.96 | 12.60 | G103 | 1.05 | 1.12 | 0.91 | 1.00 | 9.66 |
| G19 | 1.49 | 0.84 | 0.85 | 0.94 | 14.13 | G104 | 1.00 | 1.27 | 0.92 | 1.02 | 9.14 |
| G20 | 0.55 | 1.01 | 0.95 | 1.05 | 4.60 | G105 | 0.97 | 0.76 | 0.90 | 1.00 | 8.80 |
| G21 | 2.23 | 1.28 | 0.82 | 0.91 | 21.60 | G106 | 0.87 | 0.92 | 0.92 | 1.01 | 7.86 |
| G22 | 1.69 | 1.23 | 0.86 | 0.95 | 16.20 | G107 | 0.50 | 0.92 | 0.96 | 1.06 | 4.07 |
| G23 | 1.53 | 0.90 | 0.86 | 0.94 | 14.53 | G108 | 0.83 | 1.23 | 0.93 | 1.03 | 7.40 |
| G24 | 1.60 | 1.01 | 0.86 | 0.95 | 15.20 | G109 | 0.99 | 1.06 | 0.91 | 1.01 | 9.07 |
| G25 | 0.81 | 1.28 | 0.94 | 1.03 | 7.26 | G110 | 1.18 | 1.16 | 0.90 | 0.99 | 10.93 |
| G26 | 0.77 | 0.80 | 0.93 | 1.02 | 6.80 | G111 | 1.10 | 0.65 | 0.88 | 0.97 | 10.14 |
| G27 | 0.99 | 0.94 | 0.91 | 1.00 | 9.00 | G112 | 1.11 | 0.61 | 0.87 | 0.96 | 10.27 |
| G28 | 1.01 | 1.11 | 0.91 | 1.01 | 9.27 | G113 | 0.72 | 0.60 | 0.92 | 1.01 | 6.34 |
| G29 | 1.33 | 1.09 | 0.89 | 0.98 | 12.47 | G114 | 0.62 | 0.96 | 0.95 | 1.04 | 5.27 |
| G30 | 1.05 | 1.04 | 0.91 | 1.00 | 9.66 | G115 | 1.73 | 0.82 | 0.83 | 0.92 | 16.53 |
| G31 | 1.02 | 1.04 | 0.91 | 1.00 | 9.34 | G116 | 0.70 | 1.31 | 0.95 | 1.04 | 6.07 |
| G32 | 1.09 | 1.31 | 0.91 | 1.01 | 10.07 | G117 | 0.78 | 0.76 | 0.92 | 1.02 | 6.93 |
| G33 | 0.58 | 1.42 | 0.96 | 1.06 | 4.93 | G118 | 0.85 | 0.70 | 0.91 | 1.01 | 7.60 |
| G34 | 1.10 | 0.77 | 0.89 | 0.98 | 10.20 | G119 | 0.96 | 1.30 | 0.92 | 1.02 | 8.74 |
| G35 | 0.89 | 0.79 | 0.91 | 1.01 | 8.00 | G120 | 0.49 | 0.54 | 0.95 | 1.04 | 4.00 |
| G36 | 1.25 | 0.73 | 0.87 | 0.96 | 11.73 | G121 | 0.47 | 1.05 | 0.96 | 1.06 | 3.73 |
| G37 | 0.66 | 0.81 | 0.94 | 1.03 | 5.73 | G122 | 0.93 | 1.07 | 0.92 | 1.01 | 8.47 |
| G38 | 0.83 | 0.89 | 0.92 | 1.02 | 7.47 | G123 | 0.95 | 0.58 | 0.89 | 0.98 | 8.60 |
| G39 | 1.54 | 1.06 | 0.87 | 0.95 | 14.60 | G124 | 0.98 | 1.32 | 0.92 | 1.02 | 8.93 |
| G40 | 1.27 | 0.99 | 0.88 | 0.98 | 11.93 | G125 | 0.73 | 0.82 | 0.93 | 1.03 | 6.40 |
| G41 | 1.58 | 0.93 | 0.85 | 0.94 | 15.00 | G126 | 1.30 | 1.24 | 0.89 | 0.99 | 12.20 |
| G42 | 1.67 | 1.17 | 0.86 | 0.95 | 16.00 | G127 | 0.62 | 0.59 | 0.93 | 1.03 | 5.34 |
| G43 | 1.82 | 1.24 | 0.85 | 0.94 | 17.46 | G128 | 0.93 | 1.08 | 0.92 | 1.02 | 8.40 |
| G44 | 1.45 | 0.68 | 0.84 | 0.93 | 13.73 | G129 | 0.58 | 0.63 | 0.94 | 1.04 | 4.86 |
| G45 | 0.80 | 0.93 | 0.93 | 1.02 | 7.13 | G130 | 0.63 | 0.53 | 0.93 | 1.02 | 5.40 |
| G46 | 0.81 | 1.20 | 0.93 | 1.03 | 7.26 | G131 | 0.83 | 0.96 | 0.93 | 1.02 | 7.47 |
| G47 | 1.06 | 0.74 | 0.89 | 0.98 | 9.80 | G132 | 1.33 | 0.70 | 0.86 | 0.95 | 12.54 |
| G48 | 0.81 | 1.40 | 0.94 | 1.04 | 7.26 | G133 | 0.93 | 0.74 | 0.90 | 1.00 | 8.47 |
| G49 | 1.27 | 1.16 | 0.89 | 0.99 | 11.93 | G134 | 0.95 | 1.00 | 0.92 | 1.01 | 8.60 |
| G50 | 1.12 | 1.41 | 0.91 | 1.01 | 10.40 | G135 | 0.98 | 0.52 | 0.88 | 0.97 | 8.93 |
| G51 | 1.09 | 0.55 | 0.87 | 0.96 | 10.03 | G136 | 0.87 | 0.88 | 0.92 | 1.01 | 7.86 |
| G52 | 0.69 | 0.92 | 0.94 | 1.03 | 6.00 | G137 | 0.73 | 0.56 | 0.92 | 1.01 | 6.40 |
| G53 | 0.51 | 0.70 | 0.95 | 1.05 | 4.13 | G138 | 0.83 | 0.49 | 0.90 | 0.99 | 7.47 |
| G54 | 0.87 | 1.15 | 0.93 | 1.02 | 7.80 | G139 | 0.97 | 1.12 | 0.92 | 1.01 | 8.80 |
| G55 | 0.64 | 1.43 | 0.95 | 1.05 | 5.54 | G140 | 0.93 | 1.04 | 0.92 | 1.01 | 8.46 |
| G56 | 0.87 | 1.16 | 0.93 | 1.02 | 7.87 | G141 | 1.03 | 1.46 | 0.92 | 1.02 | 9.47 |
| G57 | 0.59 | 0.58 | 0.94 | 1.03 | 4.94 | G142 | 0.87 | 0.49 | 0.89 | 0.98 | 7.80 |
| G58 | 2.25 | 0.97 | 0.80 | 0.88 | 21.87 | G143 | 0.78 | 0.77 | 0.92 | 1.02 | 6.94 |
| G59 | 0.83 | 0.74 | 0.92 | 1.01 | 7.40 | G144 | 0.67 | 0.89 | 0.94 | 1.04 | 5.80 |
| G60 | 1.02 | 1.19 | 0.92 | 1.01 | 9.40 | G145 | 1.59 | 0.96 | 0.85 | 0.94 | 15.13 |
| G61 | 0.68 | 0.70 | 0.93 | 1.03 | 5.93 | G146 | 0.79 | 0.74 | 0.92 | 1.02 | 7.00 |
| G62 | 0.85 | 0.57 | 0.90 | 0.99 | 7.67 | G147 | 0.81 | 1.01 | 0.93 | 1.03 | 7.20 |
| G63 | 1.32 | 0.87 | 0.87 | 0.96 | 12.40 | G148 | 0.89 | 0.91 | 0.92 | 1.01 | 8.07 |
| G64 | 1.03 | 1.19 | 0.92 | 1.01 | 9.46 | G149 | 1.81 | 1.18 | 0.85 | 0.94 | 17.34 |
| G65 | 0.18 | 0.87 | 0.99 | 1.09 | 0.87 | G150 | 1.31 | 1.12 | 0.89 | 0.98 | 12.34 |
| G66 | 0.34 | 0.83 | 0.97 | 1.07 | 2.46 | G151 | 0.70 | 0.80 | 0.93 | 1.03 | 6.13 |
| G67 | 0.45 | 0.45 | 0.95 | 1.04 | 3.60 | G152 | 1.08 | 0.68 | 0.88 | 0.98 | 9.93 |
| G68 | 0.95 | 0.44 | 0.88 | 0.97 | 8.67 | G153 | 1.37 | 1.08 | 0.88 | 0.97 | 12.93 |
| G69 | 0.93 | 1.33 | 0.93 | 1.02 | 8.40 | G154 | 0.70 | 0.61 | 0.92 | 1.02 | 6.13 |
| G70 | 0.88 | 1.09 | 0.93 | 1.02 | 7.93 | G155 | 0.91 | 0.88 | 0.91 | 1.01 | 8.20 |
| G71 | 0.79 | 0.43 | 0.90 | 0.99 | 7.00 | G156 | 1.10 | 0.81 | 0.89 | 0.98 | 10.13 |
| G72 | 0.85 | 1.38 | 0.94 | 1.03 | 7.60 | G157 | 1.48 | 0.82 | 0.85 | 0.94 | 14.07 |
| G73 | 0.75 | 1.05 | 0.94 | 1.03 | 6.60 | G158 | 1.29 | 0.71 | 0.86 | 0.95 | 12.07 |
| G74 | 0.60 | 0.87 | 0.95 | 1.04 | 5.07 | G159 | 1.01 | 0.66 | 0.89 | 0.98 | 9.26 |
| G75 | 0.77 | 0.75 | 0.92 | 1.02 | 6.80 | G160 | 1.00 | 0.92 | 0.91 | 1.00 | 9.20 |
| G76 | 0.35 | 0.63 | 0.97 | 1.07 | 2.54 | G161 | 1.30 | 0.88 | 0.88 | 0.97 | 12.20 |
| G77 | 0.81 | 1.19 | 0.93 | 1.03 | 7.27 | G162 | 1.12 | 1.13 | 0.91 | 1.00 | 10.33 |
| G78 | 0.78 | 0.52 | 0.91 | 1.00 | 6.93 | G163 | 0.77 | 0.76 | 0.92 | 1.02 | 6.80 |
| G79 | 0.65 | 0.76 | 0.94 | 1.03 | 5.60 | G164 | 0.85 | 0.72 | 0.91 | 1.01 | 7.60 |
| G80 | 1.15 | 0.95 | 0.89 | 0.99 | 10.66 | G165 | 1.04 | 0.98 | 0.91 | 1.00 | 9.53 |
| G81 | 0.84 | 0.99 | 0.93 | 1.02 | 7.53 | G166 | 1.08 | 0.75 | 0.89 | 0.98 | 10.00 |
| G82 | 0.72 | 0.98 | 0.94 | 1.03 | 6.26 | G167 | 1.02 | 1.05 | 0.91 | 1.01 | 9.33 |
| G83 | 0.65 | 0.79 | 0.94 | 1.03 | 5.60 | G168 | 1.12 | 1.16 | 0.91 | 1.00 | 10.40 |
| G84 | 0.55 | 0.83 | 0.95 | 1.05 | 4.60 | G169 | 1.46 | 1.19 | 0.88 | 0.97 | 13.80 |
| G85 | 0.60 | 0.68 | 0.94 | 1.04 | 5.14 |  |  |  |  |  |  |

SSI, STI, YSI, RSI, and TOL were the abbreviations of stress susceptibility index, stress tolerance index, yield stability index, relative stress index, tolerance index respectively.

| TABLE S16. Calculated drought tolerance and susceptibility indices in 169 wheat accessions based on the thousand grain weight (TGW) indices in 2021-2022. | | | | | | | | | | | |
| --- | --- | --- | --- | --- | --- | --- | --- | --- | --- | --- | --- |
| Code | SSI | STI | YSI | RSI | TOL | Code | SSI | STI | YSI | RSI | TOL |
| G1 | 1.26 | 0.81 | 0.73 | 0.94 | 10.75 | G86 | 1.13 | 0.86 | 0.76 | 0.98 | 9.57 |
| G2 | 0.81 | 0.74 | 0.82 | 1.05 | 6.54 | G87 | 0.94 | 0.76 | 0.79 | 1.01 | 7.80 |
| G3 | 1.30 | 0.72 | 0.70 | 0.91 | 11.16 | G88 | 1.11 | 0.67 | 0.74 | 0.95 | 9.36 |
| G4 | 0.83 | 0.65 | 0.80 | 1.03 | 6.73 | G89 | 0.67 | 0.81 | 0.85 | 1.10 | 5.31 |
| G5 | 1.17 | 0.80 | 0.74 | 0.96 | 9.93 | G90 | 0.87 | 0.94 | 0.82 | 1.06 | 7.16 |
| G6 | 0.59 | 0.71 | 0.87 | 1.12 | 4.51 | G91 | 1.12 | 0.61 | 0.72 | 0.93 | 9.49 |
| G7 | 0.58 | 0.90 | 0.88 | 1.14 | 4.45 | G92 | 0.75 | 0.86 | 0.84 | 1.08 | 6.05 |
| G8 | 1.01 | 0.78 | 0.78 | 1.00 | 8.42 | G93 | 1.33 | 0.63 | 0.68 | 0.88 | 11.43 |
| G9 | 0.62 | 0.75 | 0.86 | 1.11 | 4.77 | G94 | 1.33 | 0.74 | 0.70 | 0.90 | 11.48 |
| G10 | 1.22 | 0.80 | 0.73 | 0.94 | 10.44 | G95 | 1.39 | 0.75 | 0.69 | 0.89 | 12.03 |
| G11 | 1.06 | 0.88 | 0.78 | 1.00 | 8.95 | G96 | 0.86 | 0.83 | 0.81 | 1.05 | 7.08 |
| G12 | 1.36 | 0.76 | 0.70 | 0.90 | 11.73 | G97 | 0.77 | 0.81 | 0.83 | 1.07 | 6.22 |
| G13 | 0.72 | 0.64 | 0.83 | 1.06 | 5.75 | G98 | 1.05 | 0.91 | 0.78 | 1.01 | 8.82 |
| G14 | 1.34 | 0.87 | 0.72 | 0.93 | 11.54 | G99 | 0.79 | 0.76 | 0.82 | 1.06 | 6.37 |
| G15 | 0.42 | 0.92 | 0.92 | 1.19 | 2.90 | G100 | 1.25 | 0.88 | 0.74 | 0.95 | 10.73 |
| G16 | 1.00 | 0.94 | 0.80 | 1.02 | 8.35 | G101 | 1.09 | 0.80 | 0.76 | 0.98 | 9.19 |
| G17 | 1.62 | 0.65 | 0.63 | 0.81 | 14.19 | G102 | 0.76 | 0.93 | 0.85 | 1.09 | 6.06 |
| G18 | 1.51 | 0.73 | 0.67 | 0.86 | 13.11 | G103 | 1.01 | 0.90 | 0.79 | 1.02 | 8.40 |
| G19 | 1.12 | 0.74 | 0.75 | 0.96 | 9.45 | G104 | 0.61 | 0.88 | 0.87 | 1.12 | 4.73 |
| G20 | 0.93 | 0.63 | 0.77 | 1.00 | 7.66 | G105 | 0.90 | 0.74 | 0.80 | 1.02 | 7.39 |
| G21 | 0.98 | 0.92 | 0.80 | 1.03 | 8.14 | G106 | 0.88 | 0.77 | 0.80 | 1.03 | 7.24 |
| G22 | 0.51 | 0.82 | 0.90 | 1.15 | 3.74 | G107 | 0.71 | 0.76 | 0.84 | 1.08 | 5.66 |
| G23 | 1.29 | 0.91 | 0.73 | 0.94 | 11.08 | G108 | 1.20 | 0.79 | 0.74 | 0.95 | 10.25 |
| G24 | 1.10 | 0.74 | 0.75 | 0.96 | 9.31 | G109 | 0.97 | 0.74 | 0.78 | 1.00 | 8.03 |
| G25 | 1.10 | 0.78 | 0.76 | 0.97 | 9.29 | G110 | 1.15 | 0.64 | 0.72 | 0.93 | 9.79 |
| G26 | 1.64 | 0.65 | 0.63 | 0.80 | 14.33 | G111 | 1.09 | 0.93 | 0.78 | 1.00 | 9.18 |
| G27 | 1.47 | 0.75 | 0.68 | 0.87 | 12.78 | G112 | 1.01 | 0.78 | 0.78 | 1.00 | 8.45 |
| G28 | 0.90 | 0.85 | 0.81 | 1.04 | 7.46 | G113 | 1.02 | 0.80 | 0.78 | 1.00 | 8.51 |
| G29 | 0.55 | 0.77 | 0.88 | 1.14 | 4.10 | G114 | 0.52 | 0.65 | 0.88 | 1.13 | 3.87 |
| G30 | 1.00 | 0.69 | 0.77 | 0.99 | 8.31 | G115 | 0.96 | 0.64 | 0.77 | 0.99 | 7.96 |
| G31 | 1.32 | 0.87 | 0.72 | 0.93 | 11.31 | G116 | 0.65 | 0.73 | 0.85 | 1.10 | 5.07 |
| G32 | 1.49 | 0.85 | 0.69 | 0.89 | 12.97 | G117 | 1.35 | 0.67 | 0.69 | 0.88 | 11.61 |
| G33 | -0.29 | 0.56 | 1.14 | 1.47 | -3.72 | G118 | 0.45 | 0.81 | 0.91 | 1.17 | 3.18 |
| G34 | 0.93 | 0.68 | 0.78 | 1.00 | 7.69 | G119 | 0.82 | 0.79 | 0.82 | 1.05 | 6.69 |
| G35 | 0.83 | 0.75 | 0.81 | 1.05 | 6.74 | G120 | 1.21 | 0.73 | 0.72 | 0.93 | 10.35 |
| G36 | 0.68 | 0.73 | 0.85 | 1.09 | 5.32 | G121 | 0.81 | 0.68 | 0.81 | 1.04 | 6.54 |
| G37 | 1.12 | 0.80 | 0.75 | 0.97 | 9.49 | G122 | 0.78 | 0.66 | 0.82 | 1.05 | 6.25 |
| G38 | 0.24 | 0.91 | 0.96 | 1.24 | 1.28 | G123 | 1.12 | 0.88 | 0.76 | 0.98 | 9.46 |
| G39 | 1.28 | 0.71 | 0.71 | 0.91 | 10.95 | G124 | 0.94 | 0.76 | 0.79 | 1.01 | 7.78 |
| G40 | 0.84 | 0.86 | 0.82 | 1.06 | 6.88 | G125 | 1.38 | 0.73 | 0.69 | 0.89 | 11.94 |
| G41 | 0.79 | 0.83 | 0.83 | 1.07 | 6.39 | G126 | 0.73 | 0.81 | 0.84 | 1.08 | 5.79 |
| G42 | 0.93 | 0.89 | 0.80 | 1.03 | 7.74 | G127 | 0.96 | 0.87 | 0.80 | 1.02 | 7.96 |
| G43 | 1.29 | 0.67 | 0.70 | 0.90 | 11.03 | G128 | 1.05 | 0.71 | 0.76 | 0.97 | 8.83 |
| G44 | 0.87 | 0.66 | 0.79 | 1.02 | 7.09 | G129 | 1.07 | 0.83 | 0.77 | 0.99 | 8.96 |
| G45 | 0.92 | 0.72 | 0.79 | 1.02 | 7.56 | G130 | 0.92 | 0.97 | 0.81 | 1.05 | 7.62 |
| G46 | 0.93 | 0.97 | 0.81 | 1.04 | 7.73 | G131 | 0.56 | 0.88 | 0.89 | 1.14 | 4.22 |
| G47 | 1.13 | 0.78 | 0.75 | 0.97 | 9.55 | G132 | 1.09 | 0.69 | 0.74 | 0.96 | 9.21 |
| G48 | 0.79 | 0.78 | 0.83 | 1.06 | 6.36 | G133 | 1.07 | 0.71 | 0.75 | 0.97 | 9.05 |
| G49 | 1.29 | 0.90 | 0.73 | 0.94 | 11.07 | G134 | 1.06 | 0.71 | 0.76 | 0.97 | 8.89 |
| G50 | 1.47 | 0.83 | 0.69 | 0.89 | 12.71 | G135 | 0.81 | 0.82 | 0.82 | 1.06 | 6.55 |
| G51 | 1.17 | 0.91 | 0.76 | 0.98 | 9.95 | G136 | 1.19 | 0.83 | 0.74 | 0.96 | 10.12 |
| G52 | 0.97 | 0.72 | 0.78 | 1.00 | 8.11 | G137 | 1.15 | 0.84 | 0.75 | 0.97 | 9.78 |
| G53 | 0.80 | 1.07 | 0.85 | 1.09 | 6.44 | G138 | 0.31 | 0.64 | 0.94 | 1.21 | 1.86 |
| G54 | 1.65 | 0.90 | 0.67 | 0.86 | 14.44 | G139 | 0.94 | 0.92 | 0.81 | 1.04 | 7.77 |
| G55 | 0.72 | 0.72 | 0.83 | 1.07 | 5.77 | G140 | 0.93 | 0.83 | 0.80 | 1.03 | 7.70 |
| G56 | 1.02 | 0.78 | 0.77 | 1.00 | 8.52 | G141 | 1.42 | 0.98 | 0.72 | 0.93 | 12.24 |
| G57 | 1.14 | 0.78 | 0.75 | 0.96 | 9.63 | G142 | 1.20 | 0.68 | 0.72 | 0.93 | 10.25 |
| G58 | 1.14 | 0.82 | 0.75 | 0.97 | 9.66 | G143 | 0.63 | 0.66 | 0.85 | 1.10 | 4.91 |
| G59 | 0.87 | 0.64 | 0.79 | 1.02 | 7.12 | G144 | 0.20 | 0.68 | 0.97 | 1.25 | 0.84 |
| G60 | 1.10 | 0.76 | 0.75 | 0.97 | 9.26 | G145 | 1.13 | 0.83 | 0.76 | 0.97 | 9.52 |
| G61 | 0.57 | 0.72 | 0.87 | 1.12 | 4.29 | G146 | 1.72 | 0.88 | 0.65 | 0.84 | 15.04 |
| G62 | 0.46 | 0.61 | 0.89 | 1.15 | 3.27 | G147 | 1.46 | 0.68 | 0.67 | 0.86 | 12.61 |
| G63 | 1.26 | 0.84 | 0.73 | 0.94 | 10.74 | G148 | 1.24 | 0.78 | 0.73 | 0.93 | 10.63 |
| G64 | 1.58 | 1.01 | 0.69 | 0.89 | 13.80 | G149 | 1.08 | 0.86 | 0.77 | 0.99 | 9.09 |
| G65 | 0.99 | 0.72 | 0.77 | 0.99 | 8.23 | G150 | 1.17 | 0.90 | 0.76 | 0.97 | 9.93 |
| G66 | 0.85 | 0.84 | 0.82 | 1.05 | 6.93 | G151 | 0.99 | 0.72 | 0.77 | 0.99 | 8.23 |
| G67 | 0.73 | 0.83 | 0.84 | 1.08 | 5.86 | G152 | 1.06 | 0.59 | 0.73 | 0.95 | 8.95 |
| G68 | 0.89 | 0.65 | 0.79 | 1.01 | 7.32 | G153 | 1.51 | 0.81 | 0.68 | 0.87 | 13.13 |
| G69 | 0.36 | 0.65 | 0.93 | 1.19 | 2.33 | G154 | 0.87 | 0.74 | 0.80 | 1.03 | 7.16 |
| G70 | 1.16 | 0.84 | 0.75 | 0.97 | 9.81 | G155 | 1.55 | 0.84 | 0.68 | 0.87 | 13.53 |
| G71 | 0.99 | 0.54 | 0.74 | 0.96 | 8.25 | G156 | 0.98 | 0.77 | 0.78 | 1.00 | 8.15 |
| G72 | 0.99 | 0.71 | 0.77 | 0.99 | 8.22 | G157 | 1.21 | 0.68 | 0.72 | 0.92 | 10.34 |
| G73 | 1.31 | 0.81 | 0.72 | 0.92 | 11.26 | G158 | 0.41 | 0.82 | 0.92 | 1.18 | 2.80 |
| G74 | 0.99 | 0.91 | 0.79 | 1.02 | 8.29 | G159 | 1.30 | 0.69 | 0.70 | 0.90 | 11.11 |
| G75 | 0.67 | 0.70 | 0.85 | 1.09 | 5.22 | G160 | 1.23 | 0.67 | 0.71 | 0.92 | 10.52 |
| G76 | 1.23 | 0.85 | 0.74 | 0.95 | 10.49 | G161 | 0.99 | 0.80 | 0.78 | 1.01 | 8.24 |
| G77 | 0.83 | 0.73 | 0.81 | 1.04 | 6.75 | G162 | 0.88 | 0.95 | 0.82 | 1.06 | 7.25 |
| G78 | 1.28 | 0.86 | 0.73 | 0.94 | 10.98 | G163 | 1.52 | 0.95 | 0.70 | 0.90 | 13.24 |
| G79 | 1.18 | 0.93 | 0.76 | 0.98 | 9.99 | G164 | 1.38 | 0.75 | 0.69 | 0.89 | 11.93 |
| G80 | 0.92 | 0.69 | 0.78 | 1.01 | 7.57 | G165 | 1.06 | 0.68 | 0.75 | 0.97 | 8.88 |
| G81 | 0.63 | 0.72 | 0.86 | 1.10 | 4.88 | G166 | 0.55 | 0.71 | 0.88 | 1.13 | 4.10 |
| G82 | 0.96 | 0.55 | 0.75 | 0.97 | 8.00 | G167 | 0.86 | 0.57 | 0.78 | 1.00 | 7.02 |
| G83 | 0.93 | 0.72 | 0.78 | 1.01 | 7.72 | G168 | 1.21 | 0.90 | 0.75 | 0.96 | 10.31 |
| G84 | 1.49 | 0.89 | 0.69 | 0.89 | 12.92 | G169 | 0.96 | 0.71 | 0.78 | 1.00 | 7.96 |
| G85 | 1.08 | 0.83 | 0.77 | 0.99 | 9.12 |  |  |  |  |  |  |

SSI, STI, YSI, RSI, and TOL were the abbreviations of stress susceptibility index, stress tolerance index, yield stability index, relative stress index, tolerance index respectively.

| TABLE S17. Calculated drought tolerance and susceptibility indices in 169 wheat accessions based on the harvest index (HI) indices in 2021-2022. | | | | | | | | | | | |
| --- | --- | --- | --- | --- | --- | --- | --- | --- | --- | --- | --- |
| Code | SSI | STI | YSI | RSI | TOL | Code | SSI | STI | YSI | RSI | TOL |
| G1 | 0.50 | 0.79 | 0.92 | 1.12 | 2.79 | G86 | 0.90 | 0.89 | 0.84 | 1.03 | 5.83 |
| G2 | 0.17 | 0.63 | 0.99 | 1.21 | 0.29 | G87 | 0.71 | 0.92 | 0.88 | 1.08 | 4.33 |
| G3 | 0.92 | 0.94 | 0.84 | 1.03 | 5.98 | G88 | 1.21 | 0.80 | 0.78 | 0.95 | 8.11 |
| G4 | 0.69 | 0.83 | 0.88 | 1.08 | 4.20 | G89 | 1.33 | 0.85 | 0.76 | 0.93 | 9.06 |
| G5 | 1.06 | 0.93 | 0.82 | 1.00 | 7.04 | G90 | 1.21 | 1.01 | 0.80 | 0.98 | 8.11 |
| G6 | 1.21 | 0.80 | 0.78 | 0.95 | 8.11 | G91 | 1.50 | 0.72 | 0.71 | 0.87 | 10.36 |
| G7 | 0.78 | 0.91 | 0.87 | 1.06 | 4.91 | G92 | 1.99 | 0.66 | 0.62 | 0.76 | 14.05 |
| G8 | 1.04 | 0.91 | 0.82 | 1.00 | 6.84 | G93 | 1.06 | 0.93 | 0.82 | 1.00 | 7.01 |
| G9 | 0.53 | 0.68 | 0.90 | 1.11 | 3.02 | G94 | 1.09 | 0.89 | 0.81 | 0.99 | 7.24 |
| G10 | 1.67 | 0.71 | 0.68 | 0.83 | 11.62 | G95 | 0.54 | 1.18 | 0.92 | 1.13 | 3.05 |
| G11 | 0.52 | 0.89 | 0.92 | 1.12 | 2.90 | G96 | 1.53 | 0.65 | 0.70 | 0.85 | 10.56 |
| G12 | 0.84 | 0.76 | 0.84 | 1.03 | 5.33 | G97 | 1.80 | 0.54 | 0.62 | 0.76 | 12.61 |
| G13 | 0.84 | 0.70 | 0.84 | 1.02 | 5.36 | G98 | 1.24 | 0.90 | 0.78 | 0.96 | 8.34 |
| G14 | 0.57 | 0.84 | 0.90 | 1.11 | 3.31 | G99 | 1.36 | 0.79 | 0.75 | 0.91 | 9.27 |
| G15 | -0.10 | 0.75 | 1.06 | 1.30 | -1.76 | G100 | 2.01 | 0.68 | 0.62 | 0.76 | 14.16 |
| G16 | 0.96 | 0.75 | 0.82 | 1.00 | 6.29 | G101 | 0.54 | 0.76 | 0.91 | 1.11 | 3.10 |
| G17 | 1.23 | 0.77 | 0.77 | 0.94 | 8.32 | G102 | 0.80 | 0.71 | 0.85 | 1.04 | 5.08 |
| G18 | 1.19 | 0.81 | 0.78 | 0.96 | 8.02 | G103 | 1.46 | 0.97 | 0.75 | 0.92 | 10.00 |
| G19 | 0.92 | 0.73 | 0.82 | 1.01 | 5.97 | G104 | 0.84 | 0.77 | 0.84 | 1.03 | 5.34 |
| G20 | 0.28 | 0.64 | 0.96 | 1.18 | 1.14 | G105 | 0.66 | 0.87 | 0.89 | 1.09 | 3.96 |
| G21 | 0.73 | 0.82 | 0.87 | 1.07 | 4.52 | G106 | 1.08 | 0.61 | 0.77 | 0.95 | 7.15 |
| G22 | 0.67 | 0.89 | 0.89 | 1.09 | 4.05 | G107 | 0.44 | 0.72 | 0.93 | 1.13 | 2.34 |
| G23 | 0.45 | 0.86 | 0.93 | 1.14 | 2.40 | G108 | 0.89 | 1.06 | 0.86 | 1.05 | 5.75 |
| G24 | 1.04 | 0.70 | 0.79 | 0.97 | 6.90 | G109 | 1.21 | 0.77 | 0.77 | 0.95 | 8.12 |
| G25 | 1.35 | 0.94 | 0.77 | 0.94 | 9.19 | G110 | 0.44 | 0.70 | 0.93 | 1.13 | 2.29 |
| G26 | 0.81 | 0.71 | 0.84 | 1.03 | 5.13 | G111 | 1.42 | 0.91 | 0.75 | 0.92 | 9.77 |
| G27 | 1.24 | 0.91 | 0.78 | 0.96 | 8.37 | G112 | 0.61 | 0.81 | 0.89 | 1.09 | 3.62 |
| G28 | 0.48 | 0.78 | 0.92 | 1.13 | 2.61 | G113 | 1.08 | 0.90 | 0.81 | 0.99 | 7.16 |
| G29 | 0.56 | 0.71 | 0.90 | 1.10 | 3.25 | G114 | 0.92 | 0.88 | 0.84 | 1.03 | 5.95 |
| G30 | 0.67 | 0.81 | 0.88 | 1.08 | 4.05 | G115 | 1.49 | 0.79 | 0.72 | 0.89 | 10.29 |
| G31 | 0.60 | 0.72 | 0.89 | 1.09 | 3.54 | G116 | 1.28 | 0.85 | 0.77 | 0.94 | 8.67 |
| G32 | 0.99 | 0.75 | 0.81 | 0.99 | 6.46 | G117 | 0.83 | 1.00 | 0.86 | 1.06 | 5.29 |
| G33 | 0.60 | 0.98 | 0.90 | 1.11 | 3.56 | G118 | 0.68 | 0.88 | 0.88 | 1.08 | 4.14 |
| G34 | 0.98 | 0.70 | 0.81 | 0.99 | 6.41 | G119 | 1.67 | 0.73 | 0.68 | 0.84 | 11.65 |
| G35 | 0.36 | 0.69 | 0.94 | 1.15 | 1.75 | G120 | 1.99 | 0.57 | 0.60 | 0.73 | 14.01 |
| G36 | 0.41 | 1.01 | 0.94 | 1.16 | 2.11 | G121 | 1.26 | 0.62 | 0.74 | 0.91 | 8.53 |
| G37 | 0.24 | 0.82 | 0.97 | 1.19 | 0.84 | G122 | 1.66 | 0.76 | 0.69 | 0.85 | 11.53 |
| G38 | 0.23 | 0.82 | 0.98 | 1.20 | 0.76 | G123 | 0.95 | 0.87 | 0.83 | 1.02 | 6.17 |
| G39 | 1.46 | 0.75 | 0.72 | 0.89 | 10.07 | G124 | 0.66 | 0.71 | 0.88 | 1.07 | 3.98 |
| G40 | 0.33 | 0.62 | 0.95 | 1.16 | 1.52 | G125 | 0.97 | 0.83 | 0.82 | 1.01 | 6.37 |
| G41 | 0.63 | 0.94 | 0.90 | 1.10 | 3.73 | G126 | 1.19 | 0.78 | 0.78 | 0.95 | 8.03 |
| G42 | 0.87 | 0.94 | 0.85 | 1.04 | 5.57 | G127 | 1.16 | 0.77 | 0.78 | 0.96 | 7.75 |
| G43 | 0.42 | 0.90 | 0.94 | 1.15 | 2.19 | G128 | 1.38 | 0.71 | 0.73 | 0.90 | 9.41 |
| G44 | 1.10 | 0.74 | 0.79 | 0.96 | 7.33 | G129 | 1.79 | 0.83 | 0.68 | 0.83 | 12.56 |
| G45 | 1.15 | 0.72 | 0.78 | 0.95 | 7.66 | G130 | 0.52 | 0.81 | 0.91 | 1.12 | 2.90 |
| G46 | 0.58 | 0.78 | 0.90 | 1.10 | 3.41 | G131 | 0.61 | 0.74 | 0.89 | 1.09 | 3.62 |
| G47 | -0.16 | 0.81 | 1.07 | 1.31 | -2.24 | G132 | 1.78 | 0.83 | 0.68 | 0.84 | 12.49 |
| G48 | 0.90 | 0.78 | 0.83 | 1.02 | 5.82 | G133 | 1.24 | 0.78 | 0.77 | 0.94 | 8.39 |
| G49 | 0.22 | 0.81 | 0.98 | 1.20 | 0.66 | G134 | 0.86 | 0.77 | 0.84 | 1.03 | 5.53 |
| G50 | 0.80 | 0.82 | 0.86 | 1.05 | 5.02 | G135 | 0.77 | 0.64 | 0.84 | 1.03 | 4.84 |
| G51 | 0.29 | 0.78 | 0.96 | 1.18 | 1.22 | G136 | 0.74 | 1.03 | 0.88 | 1.08 | 4.62 |
| G52 | 0.65 | 0.67 | 0.87 | 1.07 | 3.95 | G137 | 1.39 | 0.85 | 0.75 | 0.92 | 9.48 |
| G53 | 0.52 | 0.89 | 0.92 | 1.12 | 2.91 | G138 | 1.45 | 0.77 | 0.73 | 0.89 | 9.98 |
| G54 | 1.04 | 0.91 | 0.82 | 1.00 | 6.88 | G139 | 1.51 | 0.89 | 0.73 | 0.90 | 10.43 |
| G55 | 0.38 | 0.91 | 0.95 | 1.16 | 1.84 | G140 | 1.46 | 0.92 | 0.75 | 0.92 | 10.01 |
| G56 | 1.26 | 0.77 | 0.76 | 0.94 | 8.51 | G141 | 0.98 | 0.82 | 0.82 | 1.01 | 6.39 |
| G57 | 1.10 | 0.85 | 0.80 | 0.98 | 7.31 | G142 | 1.14 | 0.96 | 0.81 | 0.99 | 7.61 |
| G58 | 1.33 | 0.96 | 0.77 | 0.95 | 9.04 | G143 | 1.58 | 0.85 | 0.72 | 0.88 | 10.93 |
| G59 | 0.80 | 0.91 | 0.86 | 1.06 | 5.04 | G144 | 0.86 | 0.89 | 0.85 | 1.04 | 5.50 |
| G60 | 0.38 | 0.72 | 0.94 | 1.15 | 1.88 | G145 | 0.68 | 0.99 | 0.89 | 1.09 | 4.12 |
| G61 | 0.94 | 0.90 | 0.84 | 1.02 | 6.10 | G146 | 1.03 | 0.89 | 0.82 | 1.00 | 6.80 |
| G62 | 0.16 | 0.61 | 0.99 | 1.22 | 0.21 | G147 | 1.52 | 0.89 | 0.73 | 0.90 | 10.52 |
| G63 | 0.92 | 0.91 | 0.84 | 1.03 | 5.98 | G148 | 1.07 | 0.74 | 0.79 | 0.97 | 7.08 |
| G64 | 1.53 | 0.75 | 0.71 | 0.87 | 10.53 | G149 | -0.14 | 1.22 | 1.05 | 1.29 | -2.09 |
| G65 | 0.94 | 0.85 | 0.83 | 1.02 | 6.11 | G150 | 1.25 | 0.78 | 0.77 | 0.94 | 8.46 |
| G66 | 1.27 | 0.77 | 0.76 | 0.93 | 8.61 | G151 | 1.06 | 0.80 | 0.80 | 0.98 | 7.04 |
| G67 | 1.81 | 0.85 | 0.68 | 0.84 | 12.69 | G152 | 1.15 | 0.91 | 0.80 | 0.98 | 7.72 |
| G68 | 0.99 | 0.90 | 0.83 | 1.01 | 6.51 | G153 | 1.65 | 0.68 | 0.68 | 0.83 | 11.46 |
| G69 | 1.49 | 0.94 | 0.74 | 0.91 | 10.30 | G154 | 0.65 | 0.69 | 0.88 | 1.07 | 3.93 |
| G70 | 0.44 | 0.92 | 0.93 | 1.14 | 2.34 | G155 | 1.42 | 0.75 | 0.73 | 0.89 | 9.76 |
| G71 | 0.83 | 0.84 | 0.85 | 1.04 | 5.27 | G156 | 1.78 | 0.81 | 0.68 | 0.83 | 12.48 |
| G72 | 1.17 | 0.95 | 0.80 | 0.98 | 7.85 | G157 | 0.91 | 0.80 | 0.83 | 1.02 | 5.85 |
| G73 | 1.32 | 0.90 | 0.77 | 0.94 | 8.98 | G158 | 0.47 | 0.94 | 0.93 | 1.14 | 2.55 |
| G74 | 0.77 | 0.84 | 0.86 | 1.06 | 4.80 | G159 | 1.25 | 0.72 | 0.76 | 0.93 | 8.46 |
| G75 | 1.37 | 0.67 | 0.73 | 0.89 | 9.35 | G160 | 2.15 | 0.77 | 0.62 | 0.76 | 15.22 |
| G76 | 1.84 | 0.76 | 0.66 | 0.81 | 12.88 | G161 | 1.45 | 0.84 | 0.74 | 0.91 | 9.97 |
| G77 | 0.92 | 0.81 | 0.83 | 1.02 | 5.92 | G162 | 1.52 | 0.83 | 0.73 | 0.89 | 10.47 |
| G78 | 0.50 | 0.92 | 0.92 | 1.13 | 2.80 | G163 | 1.55 | 0.74 | 0.71 | 0.86 | 10.72 |
| G79 | 1.78 | 0.74 | 0.67 | 0.82 | 12.48 | G164 | 0.63 | 0.69 | 0.88 | 1.08 | 3.79 |
| G80 | 0.45 | 0.94 | 0.93 | 1.14 | 2.37 | G165 | 0.79 | 0.93 | 0.87 | 1.06 | 4.97 |
| G81 | 1.37 | 0.83 | 0.75 | 0.92 | 9.36 | G166 | 1.67 | 0.88 | 0.71 | 0.87 | 11.60 |
| G82 | 1.33 | 0.95 | 0.77 | 0.95 | 9.04 | G167 | 1.01 | 0.87 | 0.82 | 1.00 | 6.66 |
| G83 | 1.47 | 0.75 | 0.72 | 0.88 | 10.09 | G168 | 1.78 | 0.73 | 0.67 | 0.82 | 12.43 |
| G84 | 0.87 | 0.83 | 0.84 | 1.03 | 5.59 | G169 | 0.99 | 0.73 | 0.81 | 0.99 | 6.52 |
| G85 | 1.17 | 0.91 | 0.79 | 0.97 | 7.86 |  |  |  |  |  |  |

SSI, STI, YSI, RSI, and TOL were the abbreviations of stress susceptibility index, stress tolerance index, yield stability index, relative stress index, tolerance index respectively.

| TABLE S18. Calculated drought tolerance and susceptibility indices in 169 wheat accessions based on the biological yield (BY) indices in 2021-2022. | | | | | | | | | | | |
| --- | --- | --- | --- | --- | --- | --- | --- | --- | --- | --- | --- |
| Code | SSI | STI | YSI | RSI | TOL | Code | SSI | STI | YSI | RSI | TOL |
| G1 | 0.78 | 0.99 | 0.92 | 1.03 | 88.67 | G86 | 1.57 | 0.82 | 0.84 | 0.93 | 178.00 |
| G2 | 0.42 | 0.69 | 0.95 | 1.06 | 46.67 | G87 | 3.66 | 0.95 | 0.69 | 0.76 | 417.34 |
| G3 | -0.69 | 1.35 | 1.06 | 1.18 | -80.00 | G88 | -0.66 | 0.84 | 1.08 | 1.20 | -76.66 |
| G4 | 0.85 | 0.93 | 0.91 | 1.02 | 96.66 | G89 | 0.78 | 0.93 | 0.92 | 1.03 | 88.00 |
| G5 | 0.70 | 1.16 | 0.94 | 1.04 | 78.67 | G90 | 2.36 | 0.90 | 0.78 | 0.87 | 269.33 |
| G6 | 0.11 | 1.02 | 0.99 | 1.10 | 11.33 | G91 | 0.93 | 0.86 | 0.90 | 1.01 | 105.33 |
| G7 | 0.63 | 0.78 | 0.93 | 1.04 | 71.00 | G92 | 1.94 | 1.02 | 0.82 | 0.92 | 221.34 |
| G8 | 0.52 | 0.76 | 0.94 | 1.05 | 58.00 | G93 | 0.17 | 1.23 | 0.99 | 1.10 | 18.00 |
| G9 | 0.10 | 0.94 | 0.99 | 1.10 | 10.67 | G94 | -0.18 | 0.84 | 1.02 | 1.13 | -21.33 |
| G10 | 1.40 | 1.08 | 0.87 | 0.97 | 158.67 | G95 | 3.23 | 1.29 | 0.75 | 0.83 | 368.00 |
| G11 | 0.17 | 0.99 | 0.98 | 1.09 | 18.00 | G96 | -0.83 | 0.81 | 1.10 | 1.22 | -95.33 |
| G12 | 1.73 | 0.94 | 0.84 | 0.93 | 196.66 | G97 | -0.37 | 1.01 | 1.04 | 1.15 | -43.33 |
| G13 | -0.94 | 0.57 | 1.14 | 1.26 | -108.67 | G98 | 2.39 | 0.76 | 0.76 | 0.84 | 272.67 |
| G14 | 0.41 | 0.59 | 0.95 | 1.05 | 45.34 | G99 | 1.83 | 0.98 | 0.83 | 0.92 | 208.00 |
| G15 | 1.19 | 0.80 | 0.87 | 0.97 | 135.00 | G100 | -0.71 | 0.71 | 1.09 | 1.21 | -82.67 |
| G16 | 0.90 | 0.84 | 0.91 | 1.01 | 102.00 | G101 | 3.22 | 1.29 | 0.75 | 0.84 | 366.66 |
| G17 | 3.71 | 0.84 | 0.67 | 0.74 | 423.33 | G102 | 0.35 | 0.95 | 0.97 | 1.07 | 39.00 |
| G18 | 1.36 | 1.01 | 0.87 | 0.97 | 155.00 | G103 | 0.01 | 0.67 | 1.00 | 1.11 | 0.00 |
| G19 | -0.87 | 0.82 | 1.10 | 1.23 | -100.00 | G104 | 0.52 | 0.93 | 0.95 | 1.05 | 58.34 |
| G20 | 0.93 | 0.75 | 0.90 | 1.00 | 105.33 | G105 | 1.64 | 0.86 | 0.84 | 0.93 | 186.00 |
| G21 | 1.30 | 0.80 | 0.86 | 0.96 | 147.34 | G106 | -1.01 | 0.92 | 1.11 | 1.24 | -116.67 |
| G22 | 3.64 | 1.08 | 0.70 | 0.78 | 415.34 | G107 | 1.52 | 0.99 | 0.86 | 0.95 | 172.67 |
| G23 | 2.07 | 0.65 | 0.77 | 0.86 | 236.00 | G108 | 0.44 | 0.96 | 0.96 | 1.06 | 49.34 |
| G24 | 0.24 | 0.76 | 0.97 | 1.08 | 26.00 | G109 | 1.56 | 0.81 | 0.84 | 0.93 | 177.33 |
| G25 | 0.50 | 1.03 | 0.95 | 1.06 | 56.00 | G110 | 2.83 | 1.01 | 0.75 | 0.84 | 322.00 |
| G26 | 2.89 | 1.09 | 0.76 | 0.84 | 329.33 | G111 | 0.66 | 1.00 | 0.94 | 1.04 | 74.00 |
| G27 | 1.22 | 0.97 | 0.88 | 0.98 | 138.00 | G112 | 1.98 | 0.81 | 0.80 | 0.89 | 225.34 |
| G28 | -0.43 | 0.77 | 1.05 | 1.17 | -50.00 | G113 | 1.17 | 0.80 | 0.88 | 0.97 | 133.33 |
| G29 | 0.94 | 1.01 | 0.91 | 1.01 | 106.66 | G114 | 1.88 | 0.99 | 0.83 | 0.92 | 214.00 |
| G30 | 0.20 | 0.84 | 0.98 | 1.09 | 21.33 | G115 | 0.98 | 0.75 | 0.89 | 0.99 | 111.33 |
| G31 | 1.58 | 1.11 | 0.86 | 0.96 | 180.00 | G116 | 0.11 | 0.88 | 0.99 | 1.10 | 12.00 |
| G32 | -0.35 | 0.75 | 1.04 | 1.16 | -41.33 | G117 | 2.17 | 0.99 | 0.80 | 0.89 | 247.33 |
| G33 | -0.01 | 0.81 | 1.00 | 1.11 | -2.00 | G118 | 2.58 | 0.89 | 0.76 | 0.84 | 294.00 |
| G34 | -0.41 | 0.76 | 1.05 | 1.17 | -48.00 | G119 | -1.23 | 0.81 | 1.15 | 1.28 | -141.33 |
| G35 | 0.45 | 1.16 | 0.96 | 1.07 | 50.66 | G120 | 1.15 | 0.67 | 0.87 | 0.97 | 130.67 |
| G36 | 2.28 | 1.14 | 0.81 | 0.90 | 260.00 | G121 | 2.55 | 0.73 | 0.74 | 0.82 | 290.33 |
| G37 | 0.65 | 0.86 | 0.93 | 1.04 | 73.33 | G122 | -0.65 | 1.23 | 1.06 | 1.18 | -75.34 |
| G38 | 0.72 | 0.75 | 0.92 | 1.02 | 81.33 | G123 | 0.22 | 0.85 | 0.98 | 1.09 | 24.00 |
| G39 | 0.94 | 0.95 | 0.91 | 1.01 | 107.00 | G124 | 0.33 | 0.92 | 0.97 | 1.07 | 36.67 |
| G40 | 0.16 | 0.88 | 0.98 | 1.09 | 17.34 | G125 | 1.72 | 1.05 | 0.84 | 0.94 | 195.33 |
| G41 | 0.71 | 1.24 | 0.94 | 1.04 | 80.00 | G126 | -0.04 | 0.73 | 1.01 | 1.12 | -6.00 |
| G42 | 0.53 | 0.63 | 0.94 | 1.04 | 60.00 | G127 | 1.02 | 0.81 | 0.89 | 0.99 | 116.00 |
| G43 | 0.72 | 1.01 | 0.93 | 1.03 | 81.33 | G128 | -1.58 | 0.89 | 1.19 | 1.32 | -182.00 |
| G44 | 0.35 | 0.95 | 0.97 | 1.07 | 38.67 | G129 | 0.24 | 1.10 | 0.98 | 1.09 | 26.00 |
| G45 | -0.65 | 0.79 | 1.08 | 1.20 | -75.34 | G130 | 1.72 | 0.65 | 0.81 | 0.90 | 196.00 |
| G46 | 0.58 | 0.95 | 0.94 | 1.05 | 65.33 | G131 | 1.51 | 0.72 | 0.84 | 0.93 | 172.00 |
| G47 | 1.30 | 0.78 | 0.86 | 0.96 | 147.33 | G132 | 0.39 | 0.65 | 0.95 | 1.06 | 44.00 |
| G48 | 1.26 | 0.77 | 0.87 | 0.96 | 143.00 | G133 | 0.24 | 0.77 | 0.97 | 1.08 | 26.00 |
| G49 | 1.30 | 0.96 | 0.88 | 0.97 | 147.33 | G134 | 2.28 | 0.89 | 0.78 | 0.87 | 259.34 |
| G50 | 0.66 | 0.87 | 0.93 | 1.04 | 74.67 | G135 | -0.02 | 0.58 | 1.00 | 1.12 | -3.33 |
| G51 | 2.45 | 0.87 | 0.77 | 0.85 | 279.33 | G136 | 0.50 | 0.68 | 0.94 | 1.05 | 56.67 |
| G52 | -0.90 | 0.94 | 1.10 | 1.22 | -104.00 | G137 | 2.42 | 0.83 | 0.77 | 0.85 | 275.33 |
| G53 | 1.85 | 0.81 | 0.81 | 0.90 | 210.00 | G138 | 0.27 | 0.77 | 0.97 | 1.08 | 30.00 |
| G54 | 0.33 | 0.91 | 0.97 | 1.07 | 36.66 | G139 | 0.69 | 0.97 | 0.93 | 1.04 | 77.34 |
| G55 | 1.44 | 0.80 | 0.85 | 0.95 | 164.00 | G140 | 1.29 | 0.83 | 0.87 | 0.96 | 146.00 |
| G56 | 1.91 | 0.76 | 0.80 | 0.89 | 217.33 | G141 | 1.02 | 1.00 | 0.90 | 1.00 | 115.34 |
| G57 | 0.81 | 0.82 | 0.91 | 1.02 | 91.33 | G142 | 1.68 | 0.86 | 0.83 | 0.93 | 190.66 |
| G58 | 2.60 | 0.84 | 0.75 | 0.84 | 296.67 | G143 | -0.10 | 0.80 | 1.01 | 1.12 | -12.00 |
| G59 | 0.56 | 0.95 | 0.94 | 1.05 | 62.67 | G144 | 0.94 | 0.71 | 0.89 | 0.99 | 106.66 |
| G60 | 2.00 | 1.01 | 0.82 | 0.91 | 228.00 | G145 | 0.03 | 0.78 | 1.00 | 1.11 | 2.67 |
| G61 | 2.70 | 1.09 | 0.77 | 0.86 | 307.33 | G146 | -0.30 | 0.84 | 1.03 | 1.15 | -35.34 |
| G62 | 1.60 | 0.85 | 0.84 | 0.93 | 182.00 | G147 | 2.79 | 0.95 | 0.75 | 0.83 | 318.00 |
| G63 | 1.77 | 0.92 | 0.83 | 0.92 | 201.34 | G148 | 0.22 | 0.71 | 0.98 | 1.08 | 24.00 |
| G64 | 2.00 | 0.77 | 0.80 | 0.88 | 228.00 | G149 | 4.95 | 1.09 | 0.62 | 0.69 | 565.33 |
| G65 | -0.62 | 0.92 | 1.07 | 1.19 | -72.00 | G150 | 1.47 | 0.97 | 0.86 | 0.96 | 166.67 |
| G66 | -0.15 | 1.02 | 1.02 | 1.13 | -18.67 | G151 | -0.56 | 0.92 | 1.06 | 1.18 | -64.67 |
| G67 | 1.93 | 1.06 | 0.83 | 0.92 | 220.00 | G152 | 0.97 | 1.08 | 0.91 | 1.01 | 110.00 |
| G68 | 0.57 | 0.84 | 0.94 | 1.04 | 64.66 | G153 | 2.11 | 0.92 | 0.80 | 0.89 | 240.66 |
| G69 | 0.40 | 0.84 | 0.96 | 1.06 | 44.66 | G154 | 2.69 | 0.83 | 0.74 | 0.83 | 306.00 |
| G70 | 2.56 | 1.04 | 0.78 | 0.86 | 291.33 | G155 | -1.46 | 0.82 | 1.18 | 1.31 | -168.00 |
| G71 | 3.13 | 0.91 | 0.72 | 0.80 | 356.66 | G156 | 3.03 | 0.85 | 0.72 | 0.80 | 345.34 |
| G72 | 0.80 | 0.74 | 0.91 | 1.01 | 90.66 | G157 | 3.62 | 0.96 | 0.69 | 0.77 | 413.33 |
| G73 | 0.69 | 0.70 | 0.92 | 1.02 | 78.00 | G158 | 2.08 | 0.82 | 0.79 | 0.88 | 236.66 |
| G74 | 0.01 | 0.72 | 1.00 | 1.11 | 0.33 | G159 | -0.21 | 0.94 | 1.02 | 1.14 | -25.33 |
| G75 | -0.76 | 0.95 | 1.08 | 1.20 | -87.33 | G160 | 1.04 | 0.94 | 0.90 | 1.00 | 118.00 |
| G76 | -0.97 | 0.79 | 1.12 | 1.24 | -111.33 | G161 | 0.99 | 1.11 | 0.91 | 1.01 | 112.67 |
| G77 | 1.45 | 1.26 | 0.88 | 0.98 | 164.67 | G162 | -0.48 | 0.78 | 1.06 | 1.18 | -56.00 |
| G78 | 2.11 | 1.11 | 0.82 | 0.91 | 240.00 | G163 | 0.59 | 1.05 | 0.94 | 1.05 | 66.00 |
| G79 | -0.30 | 0.97 | 1.03 | 1.15 | -35.34 | G164 | 1.02 | 1.09 | 0.91 | 1.01 | 115.33 |
| G80 | 3.99 | 0.92 | 0.66 | 0.73 | 454.66 | G165 | 2.41 | 1.06 | 0.79 | 0.88 | 274.00 |
| G81 | 0.41 | 1.00 | 0.96 | 1.07 | 46.00 | G166 | 1.45 | 1.13 | 0.87 | 0.97 | 165.33 |
| G82 | 1.73 | 1.49 | 0.87 | 0.96 | 196.66 | G167 | 1.78 | 1.07 | 0.84 | 0.94 | 202.00 |
| G83 | -0.42 | 0.82 | 1.05 | 1.17 | -48.67 | G168 | 0.61 | 0.78 | 0.93 | 1.04 | 68.67 |
| G84 | 0.12 | 0.95 | 0.99 | 1.10 | 12.67 | G169 | 1.78 | 0.91 | 0.83 | 0.92 | 202.00 |
| G85 | 2.58 | 0.91 | 0.76 | 0.85 | 294.00 |  |  |  |  |  |  |

SSI, STI, YSI, RSI, and TOL were the abbreviations of stress susceptibility index, stress tolerance index, yield stability index, relative stress index, tolerance index respectively.

| TABLE S19. Calculated drought tolerance and susceptibility indices in 169 wheat accessions based on the grain yield (GY) indices in 2021-2022. | | | | | | | | | | | |
| --- | --- | --- | --- | --- | --- | --- | --- | --- | --- | --- | --- |
| Code | SSI | STI | YSI | RSI | TOL | Code | SSI | STI | YSI | RSI | TOL |
| G1 | 0.53 | 0.77 | 0.85 | 1.17 | 58.00 | G86 | 1.10 | 0.74 | 0.71 | 0.97 | 120.00 |
| G2 | 0.17 | 0.43 | 0.93 | 1.28 | 18.00 | G87 | 1.75 | 0.88 | 0.60 | 0.83 | 192.66 |
| G3 | 0.46 | 1.27 | 0.90 | 1.23 | 49.33 | G88 | 0.64 | 0.65 | 0.81 | 1.11 | 69.33 |
| G4 | 0.71 | 0.77 | 0.80 | 1.10 | 77.34 | G89 | 1.14 | 0.79 | 0.71 | 0.97 | 124.34 |
| G5 | 1.07 | 1.07 | 0.76 | 1.04 | 116.67 | G90 | 1.59 | 0.88 | 0.63 | 0.87 | 174.67 |
| G6 | 0.87 | 0.83 | 0.77 | 1.06 | 94.66 | G91 | 1.15 | 0.58 | 0.66 | 0.91 | 126.00 |
| G7 | 0.70 | 0.71 | 0.80 | 1.10 | 76.67 | G92 | 2.10 | 0.66 | 0.50 | 0.69 | 230.67 |
| G8 | 0.78 | 0.70 | 0.78 | 1.07 | 85.34 | G93 | 0.87 | 1.15 | 0.80 | 1.10 | 94.67 |
| G9 | 0.32 | 0.63 | 0.90 | 1.24 | 34.00 | G94 | 0.62 | 0.74 | 0.82 | 1.13 | 67.33 |
| G10 | 1.69 | 0.77 | 0.59 | 0.82 | 185.33 | G95 | 1.66 | 1.51 | 0.69 | 0.95 | 182.00 |
| G11 | 0.34 | 0.88 | 0.91 | 1.25 | 36.00 | G96 | 0.72 | 0.53 | 0.77 | 1.05 | 78.00 |
| G12 | 1.05 | 0.72 | 0.72 | 0.98 | 114.66 | G97 | 1.19 | 0.55 | 0.65 | 0.89 | 130.66 |
| G13 | 0.29 | 0.37 | 0.88 | 1.21 | 31.34 | G98 | 1.59 | 0.68 | 0.59 | 0.82 | 175.00 |
| G14 | 0.40 | 0.50 | 0.86 | 1.18 | 43.33 | G99 | 1.55 | 0.76 | 0.62 | 0.85 | 170.00 |
| G15 | 0.27 | 0.59 | 0.91 | 1.25 | 28.67 | G100 | 1.01 | 0.47 | 0.67 | 0.92 | 110.66 |
| G16 | 0.85 | 0.63 | 0.75 | 1.03 | 92.66 | G101 | 1.45 | 0.97 | 0.67 | 0.92 | 158.67 |
| G17 | 1.99 | 0.64 | 0.51 | 0.71 | 218.66 | G102 | 0.63 | 0.67 | 0.81 | 1.12 | 68.67 |
| G18 | 1.24 | 0.84 | 0.69 | 0.95 | 136.00 | G103 | 0.83 | 0.64 | 0.75 | 1.04 | 90.67 |
| G19 | 0.45 | 0.56 | 0.85 | 1.17 | 48.66 | G104 | 0.70 | 0.71 | 0.80 | 1.10 | 76.34 |
| G20 | 0.47 | 0.47 | 0.83 | 1.14 | 50.66 | G105 | 0.98 | 0.73 | 0.73 | 1.01 | 107.34 |
| G21 | 0.90 | 0.65 | 0.74 | 1.02 | 98.00 | G106 | 0.43 | 0.55 | 0.86 | 1.18 | 46.00 |
| G22 | 1.71 | 0.95 | 0.62 | 0.85 | 188.00 | G107 | 0.82 | 0.69 | 0.77 | 1.05 | 89.34 |
| G23 | 0.93 | 0.56 | 0.71 | 0.98 | 101.34 | G108 | 0.76 | 1.01 | 0.82 | 1.12 | 82.33 |
| G24 | 0.69 | 0.53 | 0.77 | 1.06 | 75.33 | G109 | 1.29 | 0.62 | 0.64 | 0.88 | 141.67 |
| G25 | 1.13 | 0.97 | 0.73 | 1.01 | 124.00 | G110 | 1.15 | 0.71 | 0.69 | 0.95 | 126.00 |
| G26 | 1.44 | 0.77 | 0.64 | 0.88 | 158.00 | G111 | 1.22 | 0.91 | 0.71 | 0.97 | 133.33 |
| G27 | 1.21 | 0.90 | 0.71 | 0.97 | 132.00 | G112 | 0.99 | 0.65 | 0.72 | 0.98 | 108.66 |
| G28 | 0.10 | 0.60 | 0.97 | 1.33 | 10.00 | G113 | 1.18 | 0.70 | 0.68 | 0.94 | 128.67 |
| G29 | 0.53 | 0.70 | 0.84 | 1.16 | 58.00 | G114 | 1.24 | 0.88 | 0.70 | 0.96 | 136.00 |
| G30 | 0.43 | 0.67 | 0.87 | 1.19 | 46.67 | G115 | 1.27 | 0.58 | 0.64 | 0.88 | 139.34 |
| G31 | 0.79 | 0.77 | 0.78 | 1.08 | 86.67 | G116 | 0.87 | 0.76 | 0.76 | 1.05 | 94.67 |
| G32 | 0.52 | 0.56 | 0.83 | 1.14 | 56.67 | G117 | 1.43 | 0.97 | 0.67 | 0.93 | 156.66 |
| G33 | 0.38 | 0.77 | 0.89 | 1.22 | 40.67 | G118 | 1.34 | 0.78 | 0.66 | 0.91 | 146.66 |
| G34 | 0.45 | 0.54 | 0.85 | 1.17 | 48.67 | G119 | 0.78 | 0.58 | 0.76 | 1.04 | 85.34 |
| G35 | 0.36 | 0.80 | 0.90 | 1.23 | 38.67 | G120 | 1.50 | 0.39 | 0.52 | 0.72 | 164.00 |
| G36 | 1.07 | 1.14 | 0.76 | 1.05 | 117.33 | G121 | 1.53 | 0.46 | 0.55 | 0.75 | 167.34 |
| G37 | 0.31 | 0.69 | 0.91 | 1.24 | 33.33 | G122 | 1.10 | 0.94 | 0.73 | 1.01 | 120.67 |
| G38 | 0.32 | 0.62 | 0.90 | 1.23 | 34.00 | G123 | 0.64 | 0.72 | 0.81 | 1.12 | 70.00 |
| G39 | 1.43 | 0.69 | 0.63 | 0.86 | 157.34 | G124 | 0.53 | 0.65 | 0.84 | 1.15 | 58.00 |
| G40 | 0.21 | 0.54 | 0.93 | 1.28 | 22.00 | G125 | 1.31 | 0.83 | 0.68 | 0.93 | 143.33 |
| G41 | 0.66 | 1.16 | 0.85 | 1.17 | 71.33 | G126 | 0.68 | 0.57 | 0.78 | 1.08 | 74.00 |
| G42 | 0.64 | 0.59 | 0.80 | 1.10 | 70.00 | G127 | 1.03 | 0.61 | 0.70 | 0.96 | 112.67 |
| G43 | 0.43 | 0.91 | 0.89 | 1.22 | 46.67 | G128 | 0.47 | 0.63 | 0.85 | 1.17 | 50.66 |
| G44 | 0.84 | 0.69 | 0.76 | 1.05 | 91.34 | G129 | 1.41 | 0.88 | 0.67 | 0.91 | 154.67 |
| G45 | 0.44 | 0.57 | 0.86 | 1.17 | 47.34 | G130 | 0.80 | 0.52 | 0.74 | 1.02 | 87.34 |
| G46 | 0.55 | 0.75 | 0.84 | 1.16 | 60.00 | G131 | 0.85 | 0.53 | 0.73 | 1.00 | 93.34 |
| G47 | 0.34 | 0.61 | 0.89 | 1.22 | 36.66 | G132 | 1.23 | 0.53 | 0.63 | 0.87 | 134.66 |
| G48 | 0.96 | 0.60 | 0.71 | 0.98 | 105.34 | G133 | 0.84 | 0.60 | 0.75 | 1.03 | 91.33 |
| G49 | 0.52 | 0.78 | 0.85 | 1.17 | 56.00 | G134 | 1.27 | 0.67 | 0.66 | 0.90 | 139.34 |
| G50 | 0.72 | 0.72 | 0.80 | 1.09 | 78.00 | G135 | 0.41 | 0.35 | 0.83 | 1.14 | 44.66 |
| G51 | 0.93 | 0.67 | 0.73 | 1.01 | 102.00 | G136 | 0.62 | 0.69 | 0.82 | 1.12 | 67.34 |
| G52 | 0.21 | 0.61 | 0.93 | 1.28 | 22.66 | G137 | 1.65 | 0.69 | 0.59 | 0.80 | 181.33 |
| G53 | 0.92 | 0.73 | 0.75 | 1.03 | 100.00 | G138 | 0.98 | 0.60 | 0.71 | 0.97 | 107.33 |
| G54 | 0.83 | 0.81 | 0.78 | 1.07 | 90.67 | G139 | 1.28 | 0.86 | 0.69 | 0.94 | 140.67 |
| G55 | 0.70 | 0.74 | 0.80 | 1.10 | 76.00 | G140 | 1.39 | 0.77 | 0.65 | 0.89 | 152.67 |
| G56 | 1.42 | 0.59 | 0.61 | 0.84 | 155.34 | G141 | 1.06 | 0.82 | 0.73 | 1.00 | 116.00 |
| G57 | 0.87 | 0.68 | 0.75 | 1.03 | 95.34 | G142 | 1.33 | 0.83 | 0.67 | 0.92 | 146.00 |
| G58 | 1.81 | 0.80 | 0.58 | 0.80 | 198.67 | G143 | 0.97 | 0.68 | 0.73 | 1.00 | 106.00 |
| G59 | 0.68 | 0.87 | 0.82 | 1.13 | 74.00 | G144 | 0.89 | 0.61 | 0.73 | 1.01 | 97.34 |
| G60 | 0.90 | 0.71 | 0.75 | 1.03 | 98.00 | G145 | 0.33 | 0.77 | 0.90 | 1.24 | 35.33 |
| G61 | 1.58 | 0.98 | 0.65 | 0.89 | 173.33 | G146 | 0.53 | 0.74 | 0.85 | 1.16 | 58.00 |
| G62 | 0.51 | 0.51 | 0.83 | 1.13 | 55.34 | G147 | 2.13 | 0.82 | 0.53 | 0.73 | 234.00 |
| G63 | 1.25 | 0.83 | 0.69 | 0.95 | 137.33 | G148 | 0.77 | 0.51 | 0.75 | 1.03 | 83.67 |
| G64 | 1.60 | 0.58 | 0.57 | 0.78 | 176.00 | G149 | 1.82 | 1.31 | 0.65 | 0.89 | 199.34 |
| G65 | 0.41 | 0.76 | 0.88 | 1.21 | 44.66 | G150 | 1.41 | 0.73 | 0.64 | 0.88 | 154.67 |
| G66 | 0.87 | 0.80 | 0.77 | 1.06 | 94.67 | G151 | 0.54 | 0.73 | 0.84 | 1.16 | 58.67 |
| G67 | 2.03 | 0.90 | 0.56 | 0.77 | 222.66 | G152 | 1.16 | 0.97 | 0.73 | 1.00 | 127.33 |
| G68 | 0.96 | 0.68 | 0.73 | 1.00 | 104.67 | G153 | 1.89 | 0.59 | 0.51 | 0.71 | 208.00 |
| G69 | 1.19 | 0.78 | 0.69 | 0.95 | 130.00 | G154 | 1.22 | 0.57 | 0.65 | 0.89 | 134.00 |
| G70 | 1.11 | 0.95 | 0.73 | 1.01 | 122.00 | G155 | 0.49 | 0.60 | 0.84 | 1.16 | 53.00 |
| G71 | 1.57 | 0.76 | 0.61 | 0.84 | 172.67 | G156 | 2.23 | 0.69 | 0.49 | 0.67 | 245.33 |
| G72 | 1.15 | 0.67 | 0.68 | 0.94 | 126.00 | G157 | 1.83 | 0.77 | 0.57 | 0.78 | 201.34 |
| G73 | 1.06 | 0.62 | 0.69 | 0.95 | 116.00 | G158 | 1.02 | 0.77 | 0.73 | 1.00 | 112.00 |
| G74 | 0.46 | 0.60 | 0.85 | 1.17 | 49.33 | G159 | 0.79 | 0.69 | 0.77 | 1.06 | 86.00 |
| G75 | 0.72 | 0.64 | 0.78 | 1.08 | 78.00 | G160 | 1.95 | 0.70 | 0.53 | 0.73 | 214.67 |
| G76 | 0.85 | 0.59 | 0.74 | 1.02 | 92.33 | G161 | 1.47 | 0.94 | 0.66 | 0.91 | 161.33 |
| G77 | 1.16 | 1.03 | 0.73 | 1.01 | 127.33 | G162 | 0.78 | 0.66 | 0.77 | 1.06 | 85.34 |
| G78 | 1.08 | 1.01 | 0.75 | 1.02 | 118.67 | G163 | 1.33 | 0.77 | 0.66 | 0.91 | 145.33 |
| G79 | 1.16 | 0.72 | 0.69 | 0.95 | 126.67 | G164 | 0.72 | 0.75 | 0.80 | 1.10 | 78.00 |
| G80 | 1.66 | 0.87 | 0.62 | 0.85 | 182.67 | G165 | 1.39 | 0.98 | 0.68 | 0.94 | 152.66 |
| G81 | 1.09 | 0.84 | 0.72 | 1.00 | 119.34 | G166 | 2.09 | 0.89 | 0.55 | 0.76 | 229.33 |
| G82 | 1.76 | 1.42 | 0.67 | 0.92 | 193.33 | G167 | 1.42 | 0.90 | 0.67 | 0.92 | 156.00 |
| G83 | 0.80 | 0.62 | 0.76 | 1.04 | 87.34 | G168 | 1.34 | 0.55 | 0.61 | 0.84 | 147.00 |
| G84 | 0.60 | 0.78 | 0.83 | 1.14 | 65.33 | G169 | 1.23 | 0.66 | 0.66 | 0.91 | 134.67 |
| G85 | 1.72 | 0.84 | 0.60 | 0.83 | 188.66 |  |  |  |  |  |  |

SSI, STI, YSI, RSI, and TOL were the abbreviations of stress susceptibility index, stress tolerance index, yield stability index, relative stress index, tolerance index respectively.
